# SYNERGY-VAE: An explainable generative deep learning framework for discovering depression subgroups from multimodal population health data

**DOI:** 10.1371/journal.pdig.0001719

**Published:** 2026-09-24

**Authors:** Divya Sharma, Alice Rueda, Qiaowei Lin, Shakila Meshkat, Argyrios Perivolaris, Venkat Bhat

**Affiliations:** 1 Department of Mathematics and Statistics, York University, Toronto, Ontario, Canada; 2 Biostatistics Department, Princess Margaret Cancer Centre, University Health Network, Toronto, Ontario, Canada; 3 Biostatistics Department, Dalla Lana School of Public Health, University of Toronto, Toronto, Ontario, Canada; 4 AI for Mental Health Program, St. Michael’s Hospital, Unity Health Toronto, Toronto, Ontario, Canada; 5 Department of Electrical and Computer Engineering, Toronto Metropolitan University, Toronto, Ontario, Canada; 6 Department of Psychiatry, University of Toronto, Toronto, Ontario, Canada; National University of Singapore, SINGAPORE

## Abstract

Depression represents a complex, multifactorial disorder influenced by the interplay of biological, behavioral, and social determinants. While extensive health surveys, including the National Health and Nutrition Examination Survey (NHANES), gather comprehensive multidomain data, existing studies tend to analyze these domains independently, overlooking the potential to identify integrated, data-driven patterns of depression risk. We have developed SYNERGY-VAE, an interpretable and multimodal deep learning framework that is designed to identify latent health subgroups and facilitate the transparent prediction of depression risk, utilizing NHANES-derived multimodal data from a large analytic cohort that spans five distinct domains from 2005 to 2018. SYNERGY-VAE utilizes a variational autoencoder to learn a shared latent representation derived from five modalities of NHANES, specifically demographic, dietary, examination, laboratory, and questionnaire data. Clustering within this latent space revealed subpopulations exhibiting distinct health signatures. To enhance interpretability, we triangulated insights utilizing encoder weights, standardized mean differences, and permutation feature importance (PFI). Machine learning classifiers were trained within each cluster employing the top 30 PFI-ranked features, with performance evaluated through the receiver operating characteristic area under the curve (AUC) in a 70/30 train-test split. Three latent clusters emerged, each demonstrating markedly different observed prevalence rates of depression within the analytic sample, ranging from 6.8% to 10.9%. Cluster-specific models consistently surpassed pooled models in performance, with the highest predictive accuracy identified in Cluster 2 utilizing XGBoost (AUC = 0.839, 95% CI:0.804–0.874). The importance of features varied across clusters, indicating unique depression risk profiles specific to each subgroup. SYNERGY-VAE demonstrates the power of generative, explainable deep learning for the identification of latent health phenotypes, thereby furthering the objectives of precision mental health. By integrating high-dimensional, multimodal data and facilitating transparent subgroup discovery, our model may inform future stratified and context-aware screening research and contribute to precision psychiatry-oriented research in large health survey datasets.

## Author summary

Depression rarely has a single cause. It reflects a mix of biology, behavior, and social context that varies from person to person. We asked whether large, routinely collected health surveys could reveal hidden subgroups of people who share meaningful patterns linked to depression. Using a NHANES-derived health survey data, we combined five kinds of information: demographic details, diet, physical measures, laboratory tests, and questionnaire responses. We then used a generative deep learning approach to learn a shared, low-dimensional picture of each person’s overall health profile. Within this shared space, we found three subgroups with clearly different rates of depression and distinct health signatures. When we trained simple prediction models inside each subgroup, performance improved compared with using a single model for everyone. To keep our approach transparent, we used multiple methods to show which features shaped the subgroups and which features were most important for prediction. Our results suggest that multimodal health survey data can uncover useful depression phenotypes and may inform more tailored screening and prevention strategies. All data are public, and our code is openly available for reuse and extension.

## 1. Introduction

Depression constitutes a significant contributor to the global burden of disease and ranks as a primary cause of disability worldwide, affecting approximately 280 million individuals and substantially impairing physical, social, and occupational functioning [1,2]. Despite the widespread use of standardized screening instruments, such as the Patient Health Questionnaire (PHQ-9), for clinical assessment and diagnosis [3], the complexity and multifactorial nature of depression remain significant challenges for effective prevention and treatment. Recent literature highlights that depression emerges from dynamic interactions among biological, psychological, behavioral, and socioeconomic factors [4,5]; however, the precise interplay and relative significance of these factors are inadequately characterized.

A considerable methodological challenge arises from conventional analytical frameworks that generally assess depression risk factors in isolation or through narrowly defined, hypothesis-driven analyses. Epidemiological and clinical studies often focus separately on biological markers such as inflammation or metabolic dysregulation [6,7], psychological factors including anxiety and stress [8], or social determinants like socioeconomic status and educational attainment [9]. This fragmented analytical approach limits comprehensive understanding of depression’s multidimensional nature, potentially constraining effective intervention and personalized prevention strategies [10].

Deep learning models have shown notable efficacy in psychiatric research, particularly in predicting mental health outcomes from structured clinical data, brain imaging, or digital phenotyping data streams [11–13]. In particular, several studies have leveraged National Health and Nutrition Examination Survey (NHANES) data [14] to develop interpretable and high-performing machine learning models for depression risk assessment. For example, Vu *et al.* applied multiple supervised learning algorithms to NHANES 2013–2014 data and demonstrated that XGBoost yielded the highest AUC for depression prediction, also utilizing SHAP values for feature attribution [15]. Huang *et al.* used NHANES 2017–2020 to examine dietary predictors of depression using XGBoost, identifying specific nutritional covariates linked to depressive symptoms [16]. Wang *et al.* built models from NHANES 2017–2018 to associate heavy metal exposure with depression among older adults, achieving AUCs above 0.88 [17]. Similarly, Smith *et al.* combined genetic algorithms with interpretable ML models on NHANES 2013–2020 to analyze environmental exposure risk [18]. However, most deep learning applications in depression research have primarily remained unimodal, typically analyzing single data types independently, such as neuroimaging, text, or clinical assessments.

**Fig 1:**
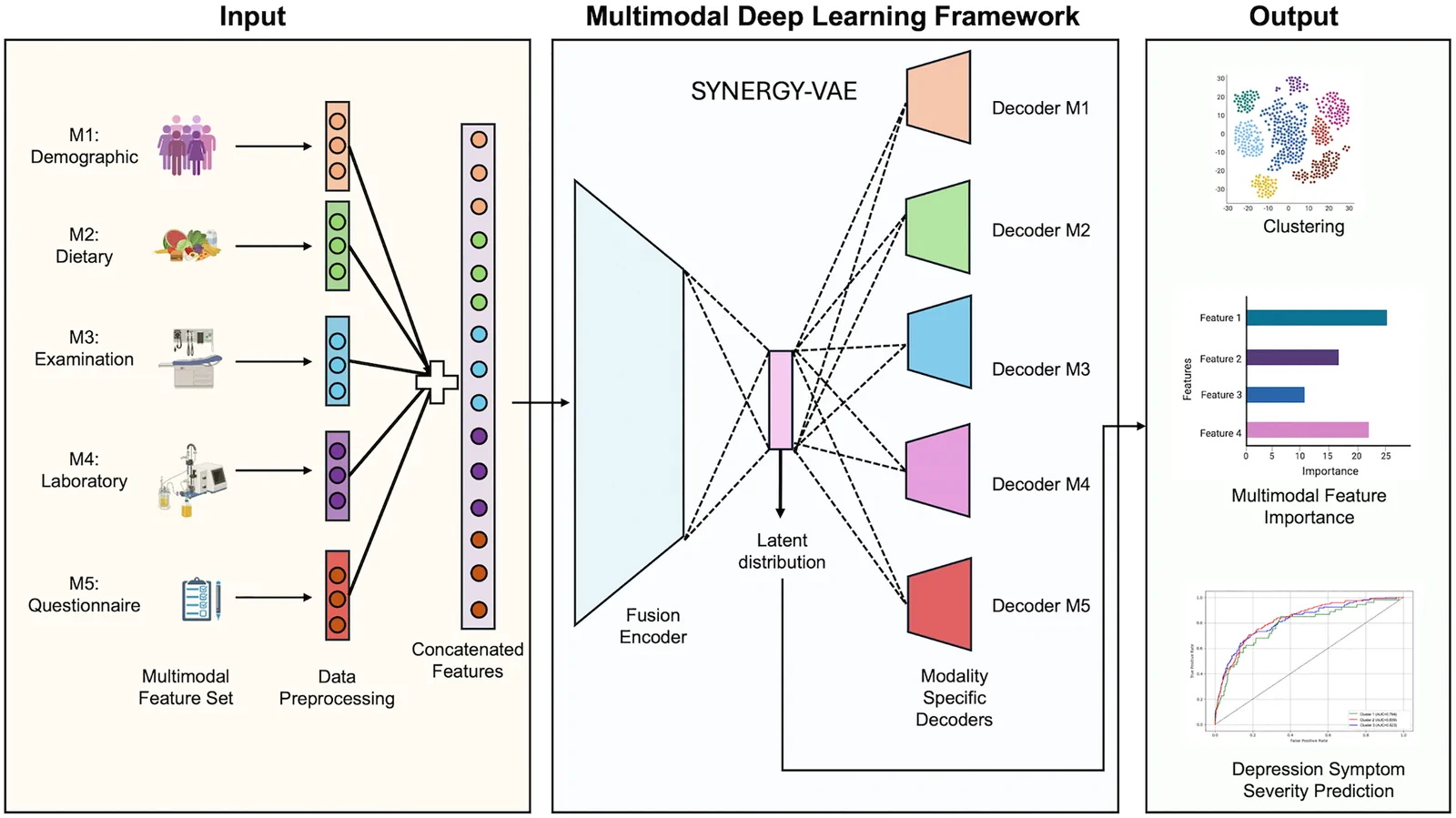
Overview of the SYNERGY-VAE multimodal deep learning framework. Five distinct data modalities from the National Health and Nutrition Examination Survey (NHANES), namely demographic, dietary, examination, laboratory, and questionnaire data, are initially preprocessed and subsequently concatenated into a unified feature set. This input is then transmitted through a shared fusion encoder that maps the multimodal data into a common latent representation. Modality-specific decoders are employed to reconstruct each domain from the latent space, thereby facilitating domain-aware reconstruction and representation learning. The resulting latent distribution is utilized for unsupervised clustering, aimed at identifying health subgroups. Feature interpretability is achieved through the analysis of encoder weights and the assessment of permutation feature importance, while cluster-specific classifiers are employed to predict the risk of depression. Created in BioRender. S, **D.** (2026) https://BioRender.com/mh97ry6.

In response to this critical gap, we present an innovative multimodal, generative deep learning framework denominated as SYNERGY-VAE (Systematic Integration of Multimodal Representations via Generative Learning) (Fig 1). SYNERGY-VAE integrates five distinct data modalities: demographic, laboratory, dietary, physical examination, and questionnaire data into a unified latent representation. SYNERGY-VAE leverages a variational autoencoder (VAE) architecture [19] with a novel single-encoder, multi-decoder design to integrate five distinct data modalities demographic, laboratory, dietary, physical examination, and questionnaire data into a unified latent representation. VAEs are generative models that learn a low-dimensional, continuous latent space by simultaneously optimizing reconstruction fidelity and distributional regularity, making them well-suited for capturing complex, nonlinear relationships across heterogeneous health domains. By implementing unsupervised clustering within this integrated latent space, we seek to identify latent health subpopulations and characterize their correlations with depression risk. Furthermore, we place a specific emphasis on interpretability, addressing the frequently cited limitation of “black-box” deep learning models [20,21].

To achieve this objective, we adopt a multi-faceted interpretability strategy that encompasses (1) encoder weight analysis to quantify the importance of features in shaping the latent space, (2) standardized mean differences (SMD) to statistically underscore distinguishing features across clusters, and (3) permutation feature importance (PFI) analysis to rigorously assess predictive relevance within cluster-specific depression classifiers.

This study contributes to the existing literature through three primary objectives: (1) developing SYNERGY-VAE to execute integrative clustering of multimodal health data acquired from NHANES, thereby uncovering latent subgroups characterized by cross-domain health profiles; (2) delineating these subgroups through a combination of statistical and deep-learning derived interpretability methodologies; and (3) assessing the association of these multimodal clusters with depression risk outcomes. To the best of our knowledge, this initiative represents the first application of a multimodal, generative deep learning architecture specifically engineered to concurrently integrate and analyze multiple NHANES domains for the purpose of identifying subpopulations associated with depression, thereby offering a novel methodological and clinical contribution to both multimodal learning and mental health research.

## 2. Methods

### 2.1. Study population

Ethics Statement: This study used publicly available, de-identified data from the National Health and Nutrition Examination Survey (NHANES), conducted by the National Center for Health Statistics (NCHS), U.S. Centers for Disease Control and Prevention (CDC). All NHANES protocols were approved by the NCHS Research Ethics Review Board (ERB) (e.g., Protocol #98–12, #2005–06, #2011–17), and written informed consent was obtained from all participants. Because the present analysis used only de-identified, publicly available data, additional Institutional Review Board approval was not required.

**Fig 2:**
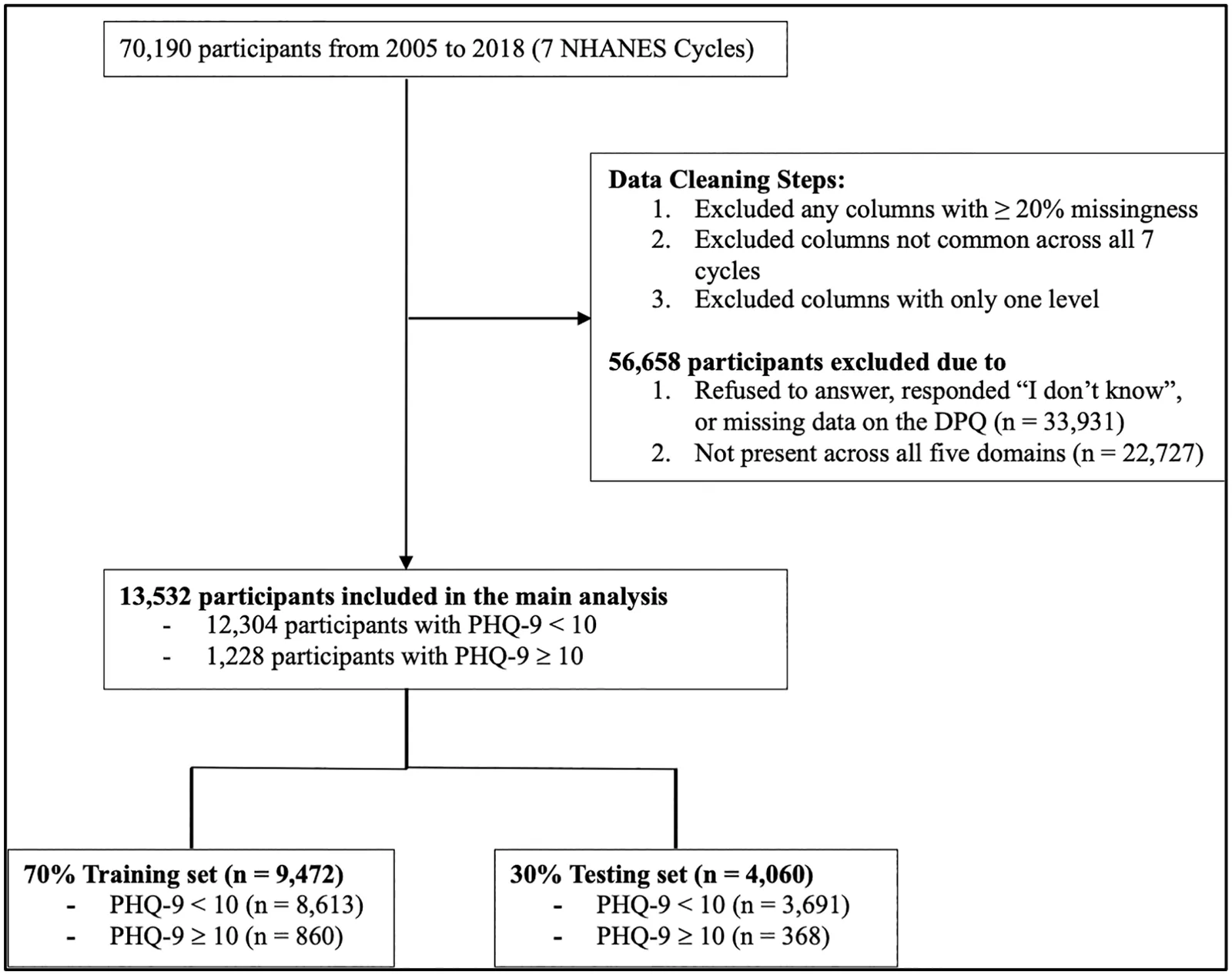
Flowchart representing study design.

The analysis utilized data from the National Health and Nutrition Examination Survey (NHANES) data, which spanned seven 2-year cycles from 2005 to 2018, encompassing a total of 13,532 individuals possessing complete multimodal data (Flowchart in Fig 2). The assessment of depression risk was derived from PHQ-9 scores, which were subsequently binarized (PHQ-9 ≥ 10 = depressed [22,23]), thereby identifying 1,228 individuals exhibiting clinically relevant depressive symptoms. In order to ensure harmonization across all cycles, a curated set of features that were consistently available throughout the cycles was developed (Fig A in S1 Appendix). This resulted in a multimodal dataset comprising five domains: demographic (19 features), dietary (10 features), examination (13 features), laboratory (63 features), and questionnaire (48 features). Each feature was meticulously mapped and validated utilizing NHANES data dictionaries. The final dataset comprised 153 variables across all domains, thereby ensuring robust cross-domain integration suitable for clustering and classification analyses and PHQ-9 items and the derived binary depression label were only used as outcome variables.

### 2.2. SYNERGY-VAE: A variational autoencoder for multimodal health data clustering

A Variational Autoencoder (VAE) is a generative model intended to learn meaningful, low-dimensional representations of high-dimensional data while preserving its underlying structure. Unlike traditional autoencoders, VAEs incorporate a probabilistic structure in the latent space which ensures a smooth, continuous, and interpretable latent representation, which makes VAEs particularly effective for modelling complex, heterogeneous datasets such as multimodal health data. By encoding diverse data sources into a shared latent space, VAEs enable downstream tasks such as clustering and prediction while maintaining the integrity of individual data modalities.

We propose SYNERGY-VAE (Systematic Integration of Multimodal Representations via Generative Learning), a novel VAE-based architecture specifically designed for clustering multimodal health data from the National Health and Nutrition Examination Survey (NHANES). Unlike standard VAEs, which are typically intended for single-domain data, SYNERGY-VAE is tailored to integrate five distinct NHANES data domains-questionnaire, laboratory, demographic, dietary, and examination data within a unified latent space. The model features a single encoder network that learns a shared latent representation, supported by five domain-specific decoder networks each tasked with reconstructing its respective input modality (Fig 1). This architectural choice constitutes a key innovation of SYNERGY-VAE, enabling integrated cross-modal representation learning while maintaining the unique statistical properties of each data domain (see Table A in S1 Appendix for architectural details).

The model is trained by optimizing the Evidence Lower Bound (ELBO) [24], which consists of two key terms: the reconstruction loss $(L_{\text{rec}})$, which measures the fidelity of reconstructed data, and the KL divergence $(L_{\text{KL}})$, which regularizes the latent space [25]. Each decoder reconstructs its respective modality from the latent space, ensuring that the generated output $(\hat{X_{m}})$ approximates the original input $(X_{m})$ for each domain ( m ).The overall loss function $(L_{\text{VAE}})$ is defined as:

$\begin{array}{c} L_{\text{VAE}}=\sum_{i=\text{1}}^{N}\sum_{m=\text{1}}^{M}\;|X_{im}-{\text{Decoder}}_{m}\left(z_{i}\right)\;{|}^{\text{2}}+\lambda \sum_{i=\text{1}}^{N}D_{\text{KL}}\left(q\left(z|X_{i}\right)|p(z)\right) \end{array}$

where  i  indexes individual samples, m  represents NHANES domains, ${\text{Decoder}}_{m}(z)$ reconstructs each domain, and λ is a regularization coefficient controlling the balance between reconstruction fidelity and latent space regularization. The KL term enforces that the learned latent distribution remains close to a standard Gaussian prior: [p(z)=N(0,I)], promoting disentanglement and improving generalizability.

Once the SYNERGY-VAE is trained, K-means clustering [19,26] on the learned latent space is used to identify distinct subpopulations within the multimodal NHANES data.

### 2.3. Depression distribution and visualization within latent clusters

In order to evaluate the risk of depression within each latent subgroup, we initially assessed the cluster-wise distribution of depression prevalence by utilizing binary labels derived from the PHQ-9. For each cluster, we calculated the proportion of individuals who met the threshold for depression and employed Fisher’s exact test with Bonferroni correction to ascertain whether any clusters demonstrated significant enrichment for depression in comparison to the broader population. To further investigate the relationship between latent structure and depression status, we generated a two-dimensional projection of the learned latent space using Uniform Manifold Approximation and Projection (UMAP), wherein we visually overlapped both cluster membership and depression labels to reveal distinct groupings displaying enrichment for individuals with depression, thereby suggesting meaningful clinical implications stratification.

### 2.4. Cluster-specific depression classification

In order to evaluate the capability to predict the risk of depression within each distinct cluster, we used supervised classification models that were trained separately for each latent subgroup. A variety of machine learning algorithms were explored, including logistic regression, random forests, gradient boosting, and XGBoost. For each cluster, the final classifier was trained utilizing the top features identified through permutation feature importance. The training and evaluation processes employed stratified 70/30 train-test splits (subsection A in S1 Appendix), while SMOTE [27] was implemented within the training data to address class imbalance, as it provides a consistent classifier-independent rebalancing strategy and may reduce overfitting relative to simple oversampling. For robustness, we performed 5-fold cross-validation during model training and applied bootstrapping (1000 resamples) on the held-out test set to estimate 95% confidence intervals around performance metrics. Model performance was assessed using Area Under the Receiver Operating Curve (AUC) along with 95% confidence intervals (CI). Additional metrics included precision, recall, sensitivity, and specificity. For comparative evaluation, we also trained pooled baseline models using the same classifiers on the full dataset without clustering. The pooled model was also evaluated within each cluster-specific held-out test subset, and ΔAUC between the cluster-specific and pooled models was estimated using 1,000 bootstrap resamples of paired held-out predictions (Table R in S1 Appendix). Reporting of the prediction modeling components was guided by the TRIPOD-AI checklist.

### 2.5. Explainability Analysis of Multimodal Clusters

In order to enhance the interpretability of the latent subgroups and to comprehend the drivers of depression risk within each cluster, we undertook a multimodal explainability analysis employing three complementary strategies: an unsupervised representation-based method, a statistical group difference analysis, and a model-based predictive feature importance strategy. Collectively, these methods provide a robust, multi-perspective understanding of the multimodal signals contributing to depression risk among patient subgroups.

#### 2.5.1. Encoder-Based Feature Contribution (Unsupervised Representation Analysis).

In order to comprehend the influence of input features on the latent representation acquired by our joint autoencoder, we conducted an analysis of the encoder’s weight matrix [28]. Specifically, we extracted the weights from the dense layer that connect each input feature to the latent space and calculated the average absolute contribution for each feature. This projection-based analysis is aimed to rank features that exert the most significant influence on the latent space, independent of any outcome label. This aims to provide insights into the manner in which various modalities contribute to the model’s compressed representation of multimodal input data and serve as a foundation for subsequent clustering activities.

#### 2.5.2. Standardized Mean difference analysis (Statistical Feature Separation).

In order to identify features that statistically differentiated the clusters, a standardized mean differences (SMD) analysis was conducted [29], comparing each cluster against all others. This analysis was performed separately within each NHANES modality. Features exhibiting SMD ≥ 0.2 were deemed meaningfully different, while thresholds of ≥ 0.5 and ≥ 0.8 indicated moderate and large effects, respectively. To account for multiple comparisons, the Benjamini-Hochberg correction was applied, resulting in the retention of features with q < 0.05. This methodology facilitated the interpretable identification of domain-specific features that were most responsible for distinguishing between the clusters.

#### 2.5.3. Permutation feature importance (Model-Based Predictive Relevance).

In order to assess the predictive relevance of features within each cluster, we employed permutation feature importance (PFI) within the framework of supervised depression classification [30]. For each cluster, features derived from all five NHANES modalities were utilized to train an initial classifier, following class rebalancing through SMOTE to mitigate label imbalance. The computation of PFI involved the iterative permutation of each feature and the subsequent measurement of the resultant decrease in model performance, quantified by the area under the ROC curve (AUC). Features that yielded greater average reductions in AUC were regarded as more significant. To ensure methodological rigor, PFI was calculated exclusively within the training folds of a stratified 5-fold cross-validation framework to prevent data leakage. The top 30 features, based on this hierarchy, were subsequently employed to retrain a final cluster-specific model, which was then evaluated on a held-out test set.

## 3. Results

### 3.1. Baseline characteristics of the study population

We analyzed data from 13,532 participants in the NHANES dataset, encompassing five domains: demographic, dietary, laboratory, examination, and questionnaire responses. The mean age of participants was 45.95 years (SD = 18.18), and 50.1% identified as female. The prevalence of depression, defined by a PHQ-9 score ≥10, was 9.1% (n = 1,228). Table 1 presents the key baseline characteristics of the study cohort stratified by depression status. Participants with depression were significantly more likely to be female (62.8% vs. 48.9%) and differed significantly in several sociodemographic and health-related variables compared to those without depression (Detailed baseline characteristics provided in Table B in S1 Appendix).

**Table 1:** Baseline characteristics of the NHANES 2005–2018 study population stratified by depressive symptoms. Participants were categorized based on PHQ-9 scores into those with (≥10) and without (<10) clinically significant depressive symptoms. Variables are presented across five NHANES domains: demographic, dietary, examination, laboratory, and questionnaire. Continuous variables are reported as mean (standard deviation), and categorical variables as counts (percentages). p-values indicate differences between groups using t-tests or chi-square tests as appropriate.

Characteristic	Total	Depressive Symptoms	Depressive Symptoms	*p*-value
		No	Yes	
Sample Size	13532	12304	1228	-
Total PHQ-9 Scores (mean (SD))	3.28 (4.38)	2.17 (2.44)	14.39 (3.99)	< 0.001
1. Demographic				
Age in years at screening (mean (SD))	45.95 (18.18)	45.93 (18.35)	46.15 (16.35)	0.686
Total # of people in the Household (mean (SD))	3.28 (1.72)	3.28 (1.72)	3.23 (1.77)	0.311
Total # of people in the Family (mean (SD))	3.06 (1.77)	3.07 (1.77)	2.96 (1.83)	0.057
Ratio of family income to poverty (mean (SD))	2.44 (1.65)	2.52 (1.65)	1.60 (1.33)	< 0.001
Sex (%)				< 0.001
Female	6784 (50.1)	6013 (48.9)	771 (62.8)	
Male	6748 (49.9)	6291 (51.1)	457 (37.2)	
Race/Hispanic origin (%)				< 0.001
Mexican American	2139 (15.8)	1945 (15.8)	194 (15.8)	
Non-Hispanic Black	2981 (22.0)	2706 (22.0)	275 (22.4)	
Non-Hispanic White	5597 (41.4)	5098 (41.4)	499 (40.6)	
Other Hispanic	1351 (10.0)	1184 (9.6)	167 (13.6)	
Other Race - Including Multi-Racial	1464 (10.8)	1371 (11.1)	93 (7.6)	
Citizenship status (%)				0.279
Citizen by birth or naturalization	11511 (85.1)	10442 (84.9)	1069 (87.1)	
Not a citizen of the US	1990 (14.7)	1835 (14.9)	155 (12.6)	
Don’t know	4 (0.0)	4 (0.0)	0 (0.0)	
Refused	22 (0.2)	19 (0.2)	3 (0.2)	
Language of SP Interview (%)				0.006
English	11862 (87.7)	10816 (87.9)	1046 (85.2)	
Spanish	1670 (12.3)	1488 (12.1)	182 (14.8)	
Proxy used in SP Interview (%)				0.706
No	13480 (99.6)	12258 (99.6)	1222 (99.5)	
Yes	52 (0.4)	46 (0.4)	6 (0.5)	
Interpreter used in SP Interview (%)				0.179
No	13221 (97.7)	12014 (97.6)	1207 (98.3)	
Yes	311 (2.3)	290 (2.4)	21 (1.7)	
2. Dietary				
Total # of Dietary Supplements taken (mean (SD))	1.12 (2.18)	1.13 (2.11)	1.05 (2.74)	0.225
Total # of Antacids Taken (mean (SD))	0.14 (1.56)	0.13 (1.48)	0.22 (2.23)	0.08
Dietary Supplements taken (%)				0.123
No	7095 (52.4)	6423 (52.2)	672 (54.7)	
Yes	6432 (47.5)	5877 (47.8)	555 (45.2)	
Refused	2 (0.0)	1 (0.0)	1 (0.0)	
Don’t know	1 (0.0)	1 (0.0)	0 (0.0)	
Antacids taken (%)				<0.001
No	12002 (88.7)	10956 (89.0)	1046 (85.2)	
Yes	1525 (11.3)	1344 (10.9)	181 (14.7)	
Refused	2 (0.0)	1 (0.0)	1 (0.1)	
Don’t know	2 (0.0)	2 (0.0)	0 (0.0)	
Number of days of intake (%)				0.552
Day 1 only	1535 (11.3)	1386 (11.3)	149 (12.1)	
Day 1 and day 2	11579 (85.6)	10541 (85.7)	41 (3.3)	
Intake day of the week (%)				0.22
Monday	1058 (7.8)	941 (7.6)	117 (9.5)	
Tuesday	1243 (9.2)	1128 (9.2)	115 (9.4)	
Wednesday	1222 (9.0)	1109 (9.0)	113 (9.2)	
Thursday	1460 (10.8)	1323 (10.8)	137 (11.2)	
Friday	3066 (22.7)	2809 (22.8)	257 (20.9)	
Saturday	2870 (21.2)	2629 (21.4)	241 (19.6)	
Sunday	2250 (16.6)	2039 (16.6)	211 (17.2)	
Language respondent used mostly - day one (%)				0.129
English	11385 (84.1)	10378 (84.3)	1007 (82.0)	
Spanish	1517 (11.2)	1352 (11.0)	165 (13.4)	
English and Spanish	149 (1.1)	136 (1.1)	13 (1.1)	
Asian Languages	50 (0.4)	47 (0.4)	3 (0.2)	
Asian Languages and English	30 (0.2)	29 (0.2)	1 (0.1)	
Other	40 (0.3)	38 (0.3)	2 (0.2)	
Language respondent used mostly- two-day (%)				0.101
English	10193 (75.3)	9295 (75.5)	898 (73.1)	
Spanish	1302 (9.6)	1163 (9.5)	139 (11.3)	
English and Spanish	86 (0.6)	78 (0.6)	8 (0.7)	
Asian Languages	62 (0.5)	61 (0.5)	1 (0.1)	
Asian Languages and English	2 (0.0)	2 (0.0)	0 (0.0)	
Other	5 (0.0)	5 (0.0)	0 (0.0)	
3. Examination				
Blood pressure: 60 sec. pulse (mean (SD))	72.73 (11.96)	72.41 (11.82)	75.91 (12.80)	<0.001
Systolic: Blood pres (1^st^ rdg) (mm Hg) (mean (SD))	122.89 (18.33)	122.95 (18.18)	122.30 (19.77)	0.255
Diastolic: Blood pres (1^st^ rdg) (mm Hg) (mean (SD))	70.11 (13.09)	70.03 (13.08)	70.95 (13.10)	0.023
Systolic: Blood pres (2^nd^ rdg) (mm Hg) (mean (SD))	122.22 (18.12)	122.25 (17.93)	121.94 (19.96)	0.566
Diastolic: Blood pres (2^nd^ rdg) (mm Hg) (mean (SD))	69.77 (13.12)	69.68 (13.06)	70.66 (13.66)	0.015
Body Measures: Standing Height (cm) (mean (SD))	167.63 (10.13)	167.84 (10.13)	165.49 (9.90)	<0.001
Body Mass Index (kg/m^2^) (mean (SD))	28.96 (7.00)	28.79 (6.80)	30.68 (8.54)	<0.001
Arm Circumference (cm) (mean (SD))	33.10 (5.14)	33.03 (5.07)	33.73 (5.75)	<0.001
Waist Circumference (cm) (mean (SD))	98.29 (16.63)	97.97 (16.40)	101.54 (18.47)	<0.001
Blood pressure: Pulse regular or irregular (%)				0.68
Regular	12943 (95.6)	11772 (95.7)	1171 (95.4)	
Irregular	325 (2.4)	296 (2.4)	29 (2.4)	
4. Laboratory				
Direct HDL-Cholesterol (mmol/L) (mean (SD))	1.36 (0.41)	1.37 (0.41)	1.31 (0.40)	<0.001
White blood cell count (1000 cells/uL) (mean (SD))	7.23 (4.16)	7.18 (4.30)	7.64 (2.36)	<0.001
Red blood cell count (million cells/uL) (mean (SD))	4.65 (0.50)	4.66 (0.50)	4.57 (0.49)	<0.001
Hemoglobin (g/dL) (mean (SD))	14.07 (1.50)	14.09 (1.50)	13.85 (1.58)	<0.001
Cotinine (ng/mL) (mean (SD))	55.54 (124.55)	51.38 (120.72)	97.62 (151.97)	<0.001
Glycohemoglobin (%) (mean (SD))	5.71 (1.06)	5.69 (1.01)	5.91 (1.44)	<0.001
Alanine aminotransferase ALT (U/L) (mean (SD))	25.62 (22.51)	25.50 (19.09)	26.84 (44.11)	0.054
Aspartate aminotransferase AST (U/L) (mean (SD))	25.94 (17.89)	25.79 (15.54)	27.48 (33.37)	0.002
Glucose, serum (mmol/L) (mean (SD))	5.61 (2.12)	5.56 (1.98)	6.05 (3.17)	<0.001
Triglycerides (mmol/L) (mean (SD))	1.71 (1.54)	1.69 (1.49)	1.90 (1.94)	<0.001
5. Questionnaire				
Ever told you had high blood pressure (%)				<0.001
No	9181 (67.8)	8505 (69.1)	676 (55.0)	
Yes	4331 (32.0)	3779 (30.7)	552 (45.0)	
Don’t know	20 (0.1)	20 (0.2)	0 (0.0)	
Current Health Status: General health condition (%)				<0.001
Excellent	1305 (9.7)	1278 (10.4)	27 (2.2)	
Very good	3645 (26.9)	3533 (28.7)	112 (9.1)	
Good	5441 (40.2)	5061 (41.1)	380 (30.9)	
Fair	2666 (19.7)	2184 (17.8)	482 (39.3)	
Poor	475 (3.5)	248 (2.0)	227 (18.5)	
Doctor told you have diabetes (%)				<0.001
No	11710 (86.5)	10754 (87.4)	956 (77.9)	
Yes	1528 (11.3)	1289 (10.5)	239 (19.5)	
Borderline	287 (2.1)	257 (2.1)	30 (2.4)	
Don’t know	7 (0.1)	4 (0.0)	3 (0.2)	
Taking insulin now (%)				<0.001
No	13096 (96.8)	11936 (97.0)	1160 (94.5)	
Yes	434 (3.2)	366 (3.0)	68 (5.5)	
Don’t know	1 (0.0)	1 (0.0)	0 (0.0)	
Seen mental health professional/past yr (%)				<0.001
No	12416 (91.8)	11509 (93.5)	907 (73.9)	
Yes	1115 (8.2)	794 (6.5)	321 (26.1)	
Don’t know	1 (0.0)	1 (0.0)	0 (0.0)	
Taken prescription medicine, past month (%)				0.207
No	6227 (46.0)	5856 (47.6)	371 (30.2)	
Yes	7302 (54.0)	6445 (52.4)	857 (69.8)	
Refused	2 (0.0)	2 (0.0)	0 (0.0)	
Ever told doctor had trouble sleeping (%)				<0.001
No	10226 (75.6)	9699 (78.8)	527 (42.9)	
Yes	3304 (24.4)	2603 (21.2)	701 (57.1)	
Don’t know	2 (0.0)	2 (0.0)	0 (0.0)	
How do you consider your weight (%)				<0.001
About the right weight	5723 (42.3)	5361 (43.6)	362 (29.5)	
Overweight	7039 (52.0)	6289 (51.1)	750 (61.1)	
Underweight	739 (5.5)	626 (5.1)	113 (9.2)	
Refused	3 (0.0)	3 (0.0)	0 (0.0)	
Don’t know	27 (0.2)	24 (0.2)	3 (0.2)	
Tried to lose weight in past year (%)				0.168
No	7645 (56.5)	6980 (56.7)	665 (54.2)	
Yes	4224 (31.2)	3826 (31.1)	398 (32.4)	
Refused	1 (0.0)	1 (0.0)	0 (0.0)	
Don’t know Smoke (%)	7 (0.1)	5 (0.0)	2 (0.2)	
Non-smoker	258 (1.9)	237 (1.9)	21 (1.7)	
Smoker	13274 (98.1)	12067 (98.1)	1207 (98.3)	

Note: # - number, SP - Sample Person, pres - pressure, rdg - reading, HDL - high-density lipoprotein, HH - Household

### 3.2. Clustering results using SYNERGY-VAE

**Fig 3:**
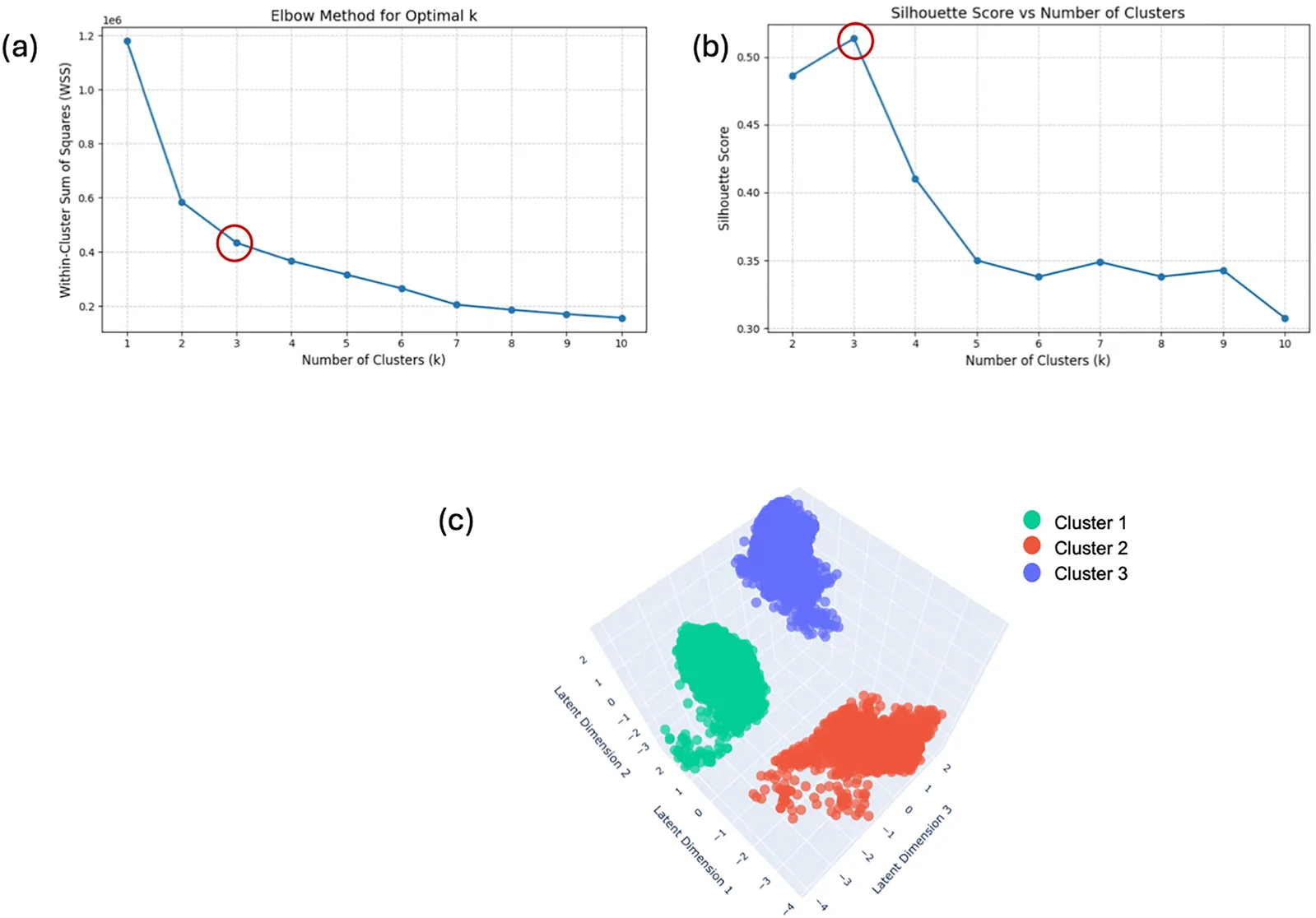
Unsupervised clustering of multimodal latent representations. **(a)** Elbow plot showing within-cluster sum of squares (WSS) across k = 1 to 10 clusters; a notable inflection is observed at k = 3, suggesting optimal compactness. **(b)** Silhouette scores for varying numbers of clusters, peaking at k = 3, indicating well-separated clusters. **(c)** 3D visualization of cluster assignments (k = 3) in the latent space, revealing three distinct and well-separated subgroups.

To uncover latent subgroups based on holistic health profiles, we applied SYNERGY-VAE, a variational autoencoder model tailored for integrating multimodal NHANES data. The encoder network learned a shared latent representation from the five data domains, and clustering was performed in this latent space using K-means. KL Regularization coefficient (λ)=1.0 provided the best overall balance of latent space structure and downstream clustering performance and was therefore retained in the final model (Table H in S1 Appendix). After evaluating latent dimensionalities ranging from 2 to 10, we found that dimensions greater than 3 (particularly 4 and 5) produced cluster structures that were highly similar to those at dimension 3, without yielding additional stratification benefits (Table N in S1 Appendix). Therefore, we selected a 3-dimensional latent space to balance model complexity with representational efficiency. All reported clustering results are based on seeded initialization to ensure replicability. Based on the elbow method and silhouette score, we selected a three-cluster solution (Fig 3(a-b)) (Table I (a-c) in S1 Appendix, Table J (a-b) in S1 Appendix). These clusters varied in size: Cluster 1 (n = 1656), Cluster 2 (n = 5970), and Cluster 3 (n = 5906). A three-dimensional visualization of the learned latent space revealed three well-separated clusters, corresponding to distinct subpopulations identified by the multimodal variational autoencoder. The clusters showed clear spatial separation, suggesting that the latent embeddings effectively captured meaningful cross-domain structure in the data (Fig 3(c)). Cluster-specific distributions of baseline characteristics are detailed in Table C in S1 Appendix. For comparison, we also experimented with standard PCA followed by K-means clustering and a simplified single-decoder VAE architecture (Tables F, G and Fig B in S1 Appendix). However, both approaches yielded less coherent cluster separation (silhouette score < 0.18) and poorer depression signal enrichment (p > 0.05), highlighting the importance of modality-specific reconstruction and non-linear integration in our SYNERGY-VAE framework.

### 3.3. Encoder-derived feature contributions

**Fig 4:**
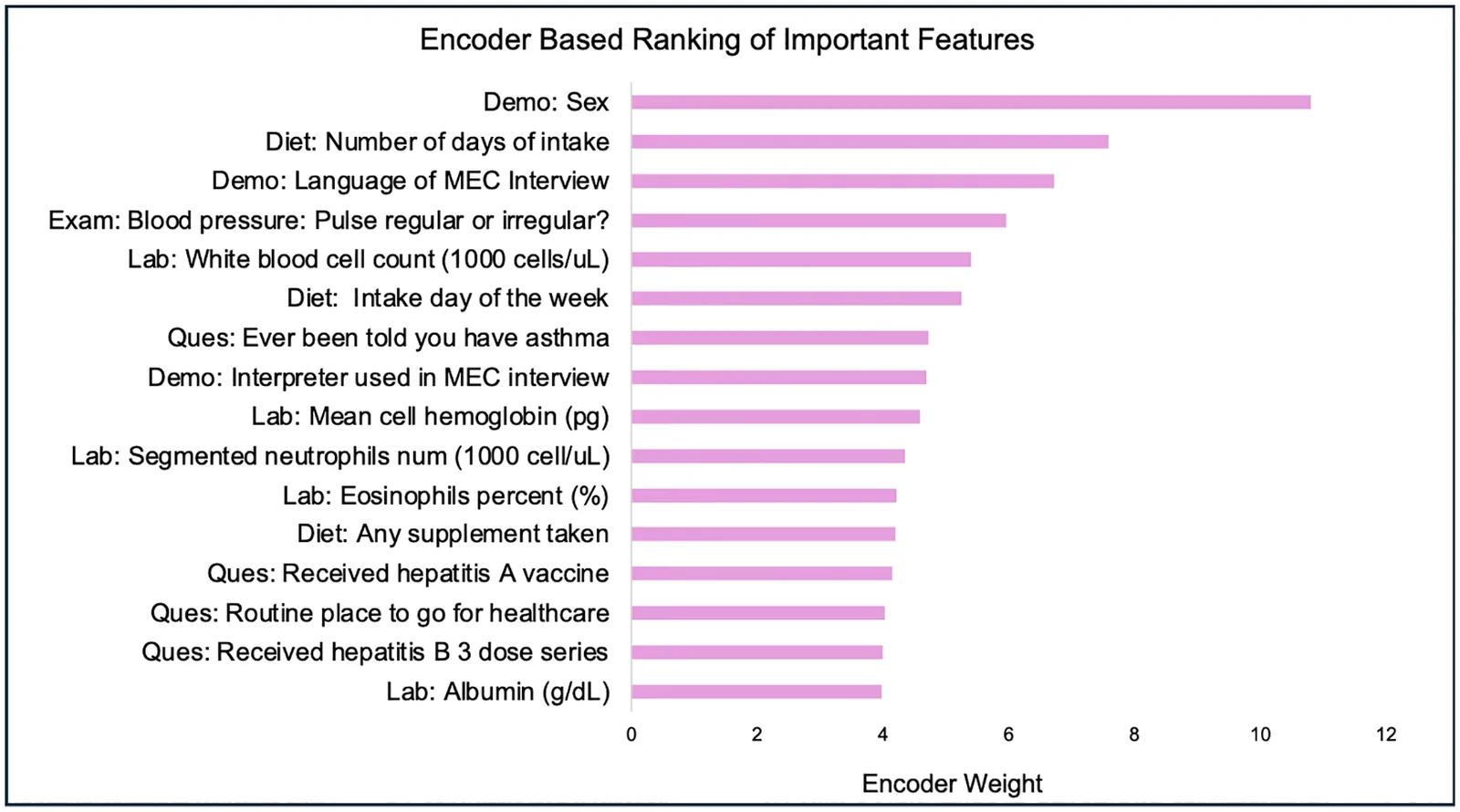
Top 20 features ranked by encoder weight contribution within the multimodal VAE architecture. These features exhibit the highest absolute weight magnitudes from the encoder layer, indicating their influence in shaping the latent representation across all modalities. To note, encoder weight magnitudes should be interpreted as a heuristic representation-level diagnostic, not as formal feature-importance estimates or evidence of causal relevance.

Analysis of the encoder weights revealed several features that contributed most strongly to the construction of the latent space (Fig 4). These included variables from all five NHANES domains, highlighting the multimodal nature of the representation. Notably influential features included sex, language of MEC interview, blood pressure pulse irregularity, and white blood cell count. Other top-ranking contributors included dietary intake timing, use of an interpreter, and various laboratory markers such as mean cell hemoglobin, segmented neutrophil count, and albumin. Several questionnaire variables such as asthma diagnosis, hepatitis vaccination status, and routine healthcare access also showed high encoder influence. These features reflect the autoencoder’s emphasis on diverse structural signals when constructing its latent representation, independent of supervised outcomes. It should be noted that encoder weight magnitudes serve as a heuristic indicator of feature influence rather than a principled importance measure, and should be interpreted alongside the more rigorous PFI and SHAP analyses rather than treated as equivalent evidence. To further interpret model behavior, we also performed SHAP analysis for the final XGBoost models (Table M and Fig C in S1 Appendix).

### 3.4. Cluster signature features based on standardized mean differences

**Fig 5:**
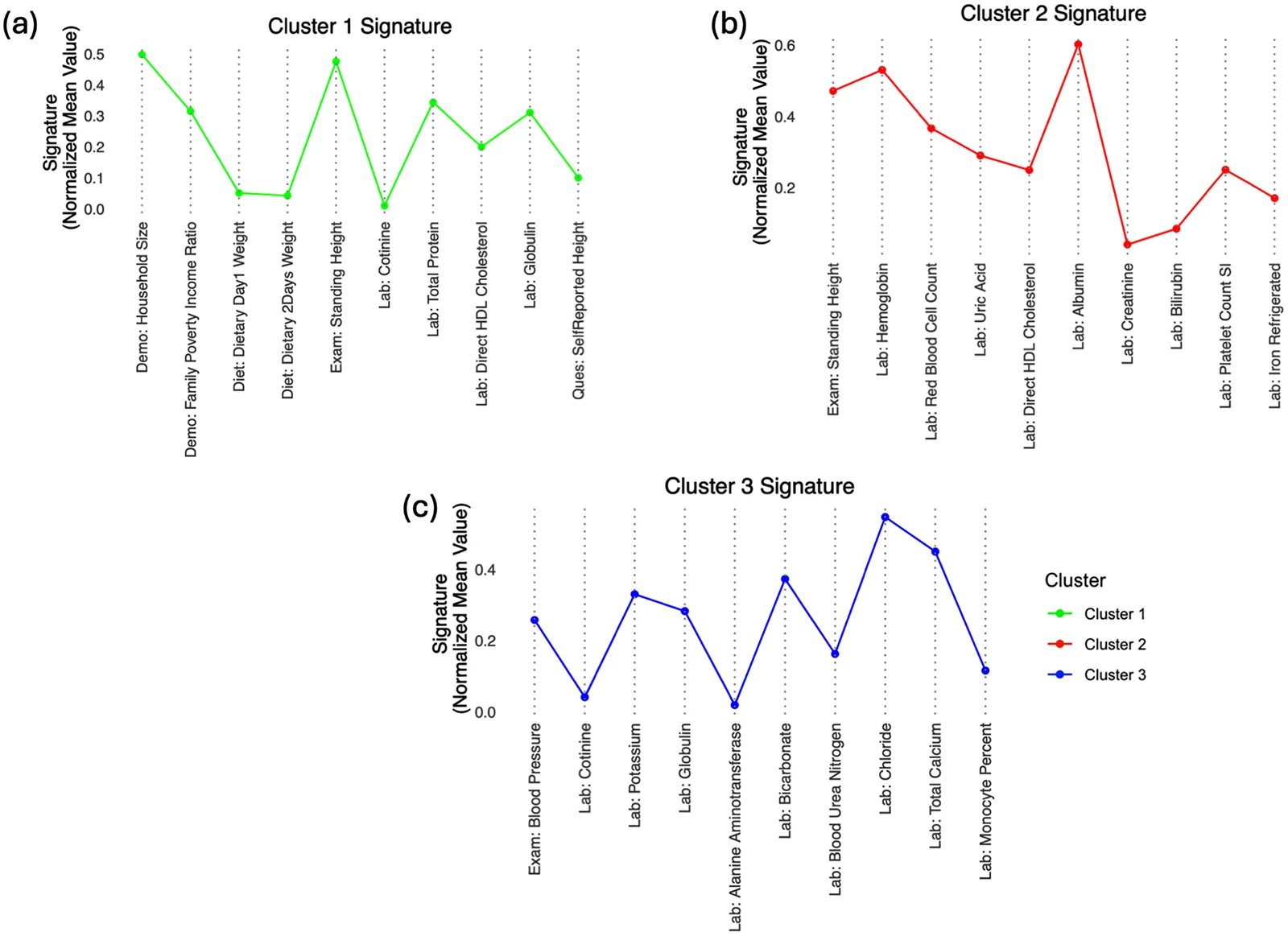
Cluster-specific signature features based on standardized mean differences (SMD). Top-ranked features contributing to the separation of each cluster, identified using standardized mean differences (SMD), and normalized across modalities. **(a)** Signature features for Cluster 1, highlighting demographic and examination variables. **(b)** Signature features for Cluster 2, characterized by hematological and metabolic markers including hemoglobin, uric acid, and direct HDL cholesterol. **(c)** Signature features for Cluster 3, dominated by laboratory and renal function indicators.

To identify statistically relevant features associated with each cluster, we calculated standardized mean differences (SMD) between each cluster and the remainder of the population across all five data domains. Features with SMD ≥ 0.5 were considered moderate to large contributors to cluster separation. As shown in Fig 5, Cluster 1 was characterized by differences in sociodemographic and anthropometric features, including family income-to-poverty ratio, standing height (cm), and dietary weight perception. Cluster 2 was distinguished by variations in hematologic and metabolic markers, such as hemoglobin (g/dL), uric acid (mg/dL), creatinine (mg/dL) and HDL cholesterol (mg/dL). Cluster 3 exhibited distinct laboratory profiles associated with renal and liver function, including blood urea nitrogen (mg/dL) and bicarbonate levels (mmol/L). These domain-specific features represent statistically meaningful, univariate contrasts that reflect the unique health signatures captured within each SYNERGY-VAE-derived cluster. These features represent statistically meaningful contrasts rather than complex multivariate patterns and are summarized in Fig 5. Benjamini-Hochberg adjusted q-values for the top 10 SMD-ranked features in each cluster are provided in Table Q in S1 Appendix.

### 3.5. Associations between clusters and depression outcomes

**Fig 6:**
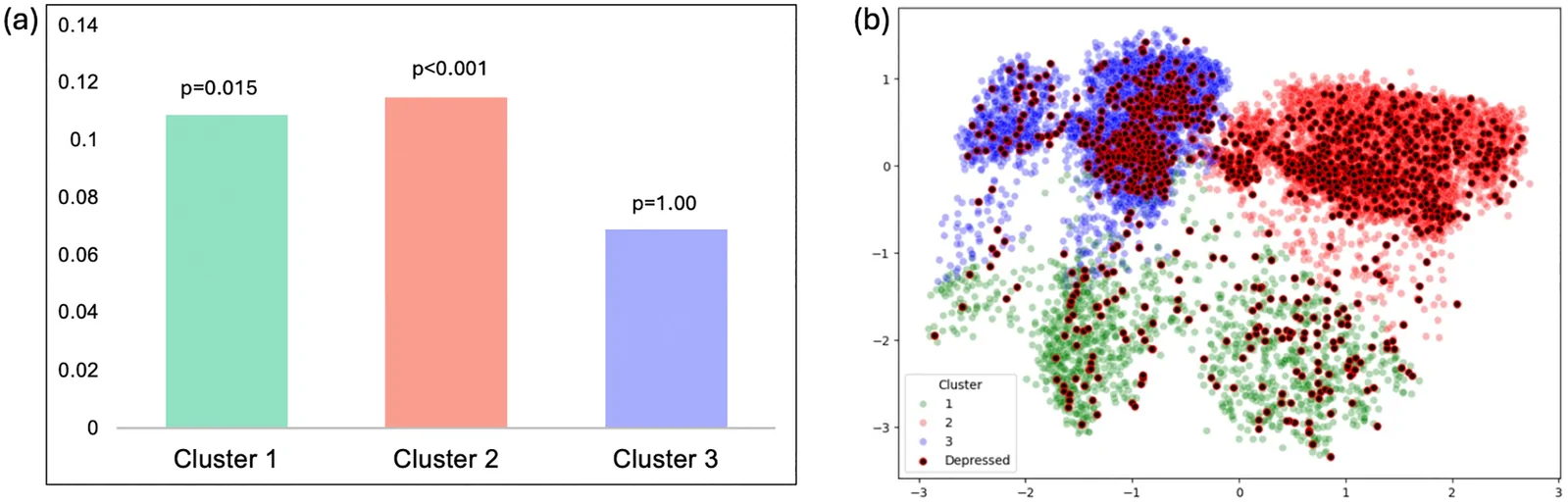
Depression prevalence and latent space visualization across multimodal clusters. **(a)** Bar plot depicting depression prevalence (%) in each of the three clusters identified by SYNERGY-VAE, showing that Cluster 3 has the lowest prevalence of depression, whereas Cluster 2 shows the highest. **(b)** UMAP visualization of the VAE latent space, illustrating clear separation between clusters. Participants with depression (black-bordered red dots) are distributed unevenly across clusters, indicating differential risk profiles captured by the multimodal latent embedding.

To assess how depression was distributed across latent subgroups, we calculated the proportion of individuals with PHQ-9 scores ≥ 10 within each cluster. As shown in Fig 6(a), depression prevalence was highest in Cluster 2 (10.9%) and Cluster 1 (10.6%), and notably lower in Cluster 3 (6.8%). Fisher’s exact tests revealed significant enrichment for depression in Clusters 1 and 2 (p < 0.05), while Cluster 3 showed a comparatively lower burden. These findings suggest that clusters derived from multimodal latent embeddings capture meaningful variation in depression risk.

To visualize the relationship between cluster membership and depression status, we projected the multimodal latent space into two dimensions using Uniform Manifold Approximation and Projection (UMAP) and overlaid the cluster assignments with the relevant depression labels (PHQ-9 ≥ 10; Fig 6(b)). Individuals diagnosed with depression are indicated as black-bordered red dots. Visual inspection indicated that cases of depression were not randomly distributed across the latent space. Cluster 2 (red) exhibited a high density of individuals experiencing depression, which aligns with its statistically significant enrichment (10.9%). Similarly, Cluster 1 (green) demonstrated a moderate concentration of depression cases (10.6%). In contrast, Cluster 3 (blue) exhibited relatively sparse overlap with depression, which is consistent with its lower depression rate (6.8%). These findings confirm that the latent clusters identified through the SYNERGY-VAE methodology effectively capture meaningful variations at the subpopulation level concerning depression risk, thereby supporting subsequent analysis stratified analyses and classification efforts.

### 3.6. Cluster-specific classification performance

We evaluated the predictive performance of four machine learning classifiers, Logistic Regression, Random Forest, Gradient Boosting, and XGBoost within each of the three depression-related clusters identified from the multimodal latent space (Table 2). Models were trained separately for each cluster using the top 30 features identified through permutation feature importance (PFI), with class imbalance addressed using SMOTE (Table L in S1 Appendix). We selected the top 30 features by permutation feature importance (PFI) to balance model interpretability and performance, based on evidence that predictive gains plateau beyond 20–50 features [31] (Table K in S1 Appendix). To ensure robustness and generalizability, we employed stratified 5-fold cross-validation, and performance was averaged across folds (Table D in S1 Appendix). Additionally, we used bootstrapping (1,000 resamples) to estimate 95% confidence intervals (CI) for the area under the ROC curve (AUC). Model performance was assessed using AUC along with 95% confidence intervals (CI). Additional metrics, including precision, recall, sensitivity, and specificity, are provided in Table E in S1 Appendix. Notably, XGBoost consistently demonstrated the highest AUC across all clusters, indicating its robustness in capturing nonlinear relationships in multimodal depression signatures. For comparison, we also trained models on the full, unclustered population, which yielded lower overall AUCs, supporting the added predictive value of stratifying individuals via SYNERGY-VAE (Table O in S1 Appendix).

**Table 2:** Performance of four classification models (XGBoost, Gradient Boosting, Logistic Regression, and Random Forest) in predicting depression within each latent cluster. Values represent the mean area under the ROC curve (AUC) along with 95% confidence intervals (CI) computed on held-out test sets. Models were trained using the top 30 cluster-specific features identified by permutation feature importance (PFI), with class imbalance addressed via SMOTE. XGBoost consistently achieved the highest AUC across all clusters.

Models	Cluster 1 (mean AUC (95% CI)	Cluster 2 (mean AUC (95% CI)	Cluster 3 (mean AUC (95% CI)
**XGBoost**	0.794 (95% CI: 0.720–0.868)	0.839 (95% CI: 0.804–0.874)	0.823 (95% CI: 0.776–0.869)
**Gradient Boosting**	0.768 (95% CI: 0.691–0.845)	0.838 (95% CI: 0.802–0.873)	0.821 (95% CI: 0.774–0.868)
**Logistic Regression**	0.763 (95% CI: 0.686–0.841)	0.793 (95% CI: 0.754–0.831)	0.792 (95% CI: 0.743–0.841)
**Random Forest**	0.759 (95% CI: 0.681–0.836)	0.826 (95% CI: 0.789–0.862)	0.811 (95% CI: 0.764–0.859)

In Cluster 1 (green; the smallest subgroup), XGBoost achieved the highest discrimination performance with a AUC of 0.794 (95% CI: 0.720–0.868), followed by Gradient Boosting (AUC = 0.768). Key features driving model performance in this cluster included Key features driving model performance in this cluster included socioeconomic and occupational indicators (poverty-income ratio, kind of work performed the longest), health-related factors (hepatitis B core antibody status, systolic blood pressure, prescription medication use), and dietary measures suggesting a socio-behavioral depression phenotype.

In Cluster 2 (red; the largest subgroup), XGBoost again outperformed all other models with a AUC of 0.839 (95% CI: 0.804–0.874), with Gradient Boosting closely behind (AUC = 0.838). Here, feature importance emphasized biologically relevant markers, including white blood cell count, blood urea nitrogen, and creatinine, highlighting an immuno-metabolic signature of depression risk unique to this subgroup.

In Cluster 3 (blue; intermediate size), XGBoost led with a AUC of 0.823 (95% CI: 0.776–0.869), marginally ahead of Gradient Boosting (AUC = 0.821). This cluster’s predictive features reflected a mixed profile, combining physiological (blood pressure, cotinine, creatinine) and behavioral variables (household food security, household size, and general health status), consistent with a more clinically complex depression presentation.

**Fig 7:**
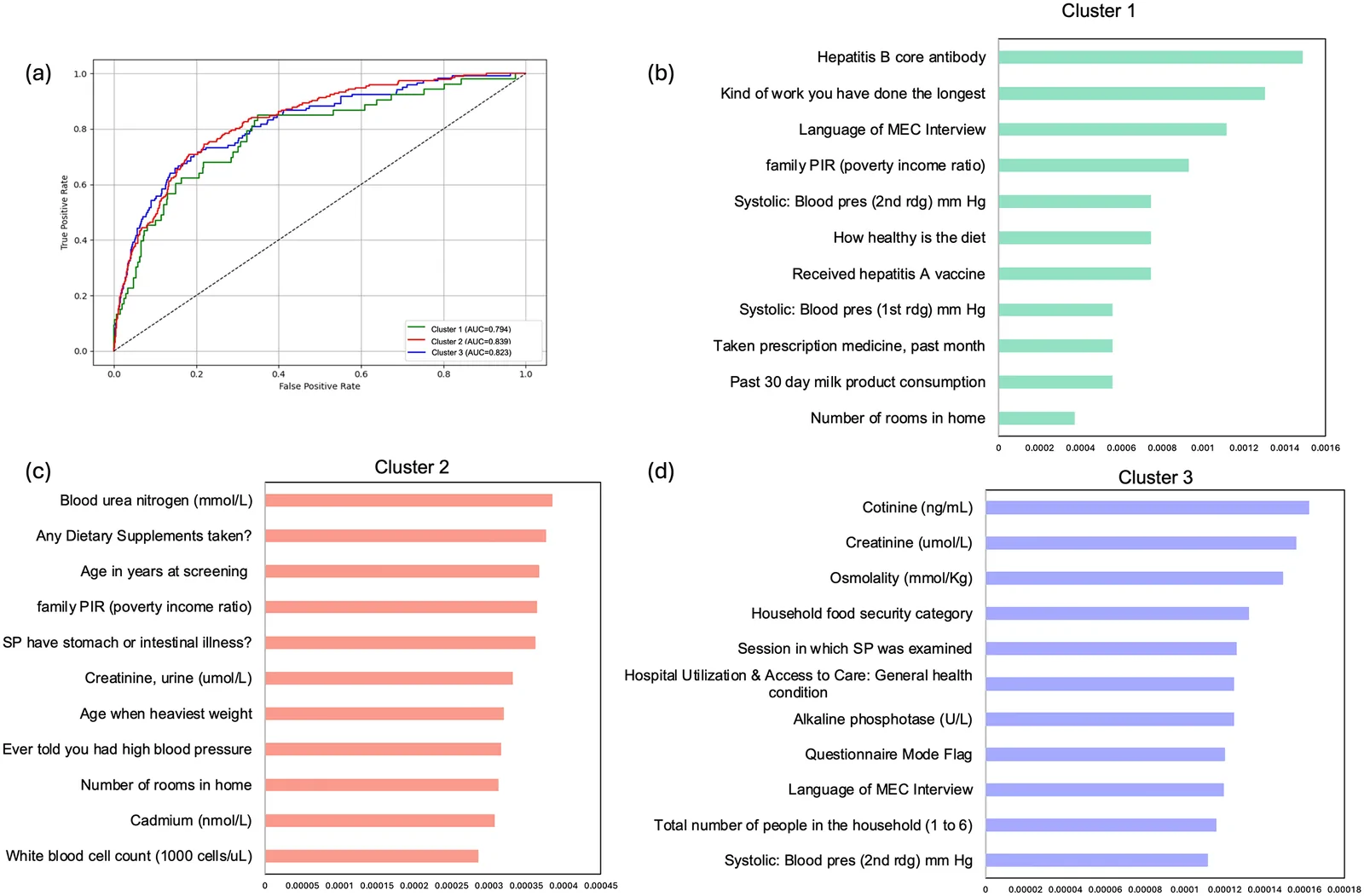
Cluster-specific model performance and feature importance. **(a)** ROC curves for XGBoost classifiers trained separately within each depression-related cluster. Cluster 2 achieved the highest AUC (0.839), followed by Cluster 3 (0.823) and Cluster 1 (0.794). **(b–d)** Top 10 features contributing to depression prediction within each cluster, based on permutation feature importance (PFI) scores. Each panel represents one cluster: **(b)** Cluster 1 (green), **(c)** Cluster 2 (red), and **(d)** Cluster 3 (blue). While some features (e.g., language preference, income, white blood cell count) appear consistently across clusters, others are cluster-specific, indicating distinct depression signatures across latent subpopulations.

Across all clusters, XGBoost consistently demonstrated superior or equivalent performance, reinforcing its robustness in modeling nonlinear, multimodal associations. These results confirm that clustering in the latent space not only reveals subgroups with differing depression prevalence but also enables precision modeling using cluster-specific features (Fig 7). The variation in top features across clusters further underscores the importance of explainable, stratified AI models tailored to distinct health subpopulations, an essential step toward equitable, personalized mental health screening in large-scale datasets. SHAP-based feature rankings showed strong concordance with PFI rankings across clusters, with Spearman correlations of 0.91, 0.93, and 0.89 for Clusters 1, 2, and 3, respectively (Table M in S1 Appendix). At the feature level, 8 of the top 10 SHAP-ranked features overlapped with the top 10 PFI-ranked features in Clusters 1 and 2, and 9 of 10 overlapped in Cluster 3. These findings support the robustness of the main predictors identified by PFI. Importantly, SHAP also provided complementary information by characterizing the direction and heterogeneity of feature effects within the cluster-specific XGBoost models, whereas PFI primarily quantified the reduction in predictive performance after feature perturbation.

## 4. Discussion

In this study, we introduced a multimodal, generative deep learning framework, SYNERGY-VAE, designed to integrate five distinct NHANES data domains: demographic, dietary, laboratory, examination, and questionnaire data, in order to identify latent subgroups associated with differing depression prevalence. Our findings demonstrate the efficacy of generative models in elucidating nuanced population structures and latent health phenotypes that may remain obscured through traditional analytic approaches.

In comparison to conventional methods, SYNERGY-VAE provides a more comprehensive perspective on individual health profiles by harnessing cross-domain interactions. While earlier clustering studies in depression research typically depended on a singular data source or employed simpler statistical models [32–35], our framework stands among the first to amalgamate such high-dimensional, multimodal health data into a latent structure that maintains interpretability through both standardized mean difference (SMD) analysis and encoder weight attribution. This constitutes a significant advancement in multimodal mental health analytics, particularly in revealing latent depression-related phenotypes that are not readily apparent through linear or univariate methods, but which become accessible with multimodal data.

SYNERGY-VAE identified three clusters with distinctive health profiles and varying prevalence rates of depression. The latent space not only facilitated robust subgrouping but also increased downstream interpretability through multiple complementary approaches: standardized mean difference (SMD) analysis, encoder weight attribution, and permutation feature importance (PFI) derived from cluster-specific depression classifiers. Importantly, these three methods of feature interpretation exhibited both converging and complementary patterns (Table M in S1 Appendix). The SHAP analysis strengthened this interpretation by showing that many of the same predictors identified through PFI also contributed substantially to individual-level model predictions. This convergence suggests that the cluster-specific predictors were not artifacts of a single interpretability method. At the same time, SHAP complemented PFI by indicating how predictor contributions varied across individuals within each cluster, thereby providing insight into within-cluster heterogeneity in model behavior. Notably, features such as physical activity level showed lower cross-method agreement, likely reflecting non-monotonic or interaction-dependent effects that permutation-based and gradient-based methods capture differently, or collinearity with related features such as BMI and dietary variables. Across all explainability strategies, features such as family income-to-poverty ratio, self-reported general health, and sleep disturbances emerged as consistent and high-impact contributors to both clustering and depression prediction. This convergence reinforces the established roles of socioeconomic status, perceived health, and sleep quality as pivotal dimensions of depression risk, in accordance with extensive prior literature concerning the biopsychosocial model of mental health. Short sleep duration, for example, has frequently been associated with an elevated depressive symptom burden [36]. Likewise, poverty-income ratio emerged as a prominent socioeconomic feature in our analysis and may reflect broader social and material disadvantage that co-occurs with depression risk [9]. Similarly, self-rated general health has been strongly associated with mental health outcomes, including depressive symptomatology [37]. The prominence of these variables in our model underscores its capacity to discern both clinically and socially meaningful indicators of depression. Our work supports a mounting body of evidence advocating for precision psychiatry-oriented research, where interventions are tailored according to multidimensional patient profiles rather than employing a one-size-fits-all methodology [11]. Our findings have several important implications. First, they support patient stratification by identifying subgroups with sufficiently distinct biopsychosocial profiles to potentially warrant differentiated screening and care pathways. Second, the cluster-specific feature importance profiles, including the socio-behavioral signature in Cluster 1 and the immuno-metabolic signature in Cluster 2, generate testable mechanistic hypotheses that can motivate targeted longitudinal or interventional studies. In particular, the prominence of immune and metabolic markers in Cluster 2, including white blood cell count, eosinophil percentage, and HDL cholesterol, is consistent with prior literature linking inflammatory and metabolic dysregulation with depression [6,7]. Third, the ability to characterize distinct depression risk phenotypes in a large NHANES-derived analytic cohort has relevance for mental health planning, including resource allocation and public health surveillance. In this sense, the unsupervised discovery of these latent phenotypes represents the central contribution of our framework, while supervised classification serves primarily as a validation and interpretability component rather than the primary endpoint.

Our results underscore the heterogeneity of depression and the necessity for personalized mental health strategies. The prevalence of depression varied markedly across latent clusters, ranging from 6.8% to 10.9%, and each cluster was characterized by distinct patterns of health-related features. For instance, one cluster reflected an immuno-metabolic risk profile, whereas another exhibited stronger psychosocial and access-related factors. These findings suggest that depressive symptoms may arise through diverse pathways and that generalized screening or treatment approaches are likely to overlook subgroup-specific signals [38,39]. These subgroup profiles also suggest potential practical applications but should be interpreted as hypothesis-generating rather than as clinically validated depression phenotypes. Cluster 1 may support potential screening framework pending prospective validation that more explicitly incorporate social and healthcare access barriers, for instance, systematic depression screening embedded within safety-net services, such as federally qualified health centers, food assistance programs, or housing support services may be better positioned to reach individuals whose risk is driven primarily by socioeconomic disadvantage. Screening in this subgroup could be triggered by combinations of low income-to-poverty ratio, poor self-rated health, sleep or stress burden, and healthcare access barriers. From a referral standpoint, this subgroup may benefit from pathways that co-locate mental health assessment with social service navigation, reflecting emerging models of integrated social prescribing. Intervention efforts in this cluster might productively target material hardship, healthcare access, and structural determinants alongside clinical symptom management. Cluster 2 may indicate a subgroup in which immuno-metabolic comorbidity could justify more proactive mental health assessment. The prominence of markers such as elevated white blood cell count, low HDL cholesterol, and elevated triglycerides in this cluster is consistent with prior evidence linking systemic inflammation and metabolic dysregulation to depression [6,7], and suggests that routine depression screening triggered by abnormal metabolic or inflammatory profiles for example in primary care, diabetes, cardiovascular prevention, or metabolic health clinics, could represent a feasible and targeted referral pathway. Intervention strategies for this subgroup might include lifestyle modification programs integrating physical activity and dietary change alongside pharmacological or psychological treatment, with bidirectional referral between primary care, endocrinology, and mental health services potentially improving case identification and follow-through. Cluster 3 may reflect a more complex multimorbidity phenotype that would benefit from coordinated, multidisciplinary screening. Individuals in this cluster, characterized by elevated markers of renal and hepatic function, may present with depression as a downstream comorbidity of advancing chronic organ disease, a pattern well-recognized in nephrology and hepatology settings. For this group, opportunistic depression screening during specialist follow-up appointments particularly in nephrology, hepatology, or complex chronic disease clinics, combined with multidisciplinary team care models that include behavioral health, may represent the most pragmatic referral pathway. Screening could be triggered by renal or hepatic laboratory abnormalities, multimorbidity burden, poor self-rated health, or sleep-related symptoms. Intervention targets in this cluster may include disease burden management, caregiver support, and psychosocial adjustment to chronic illness, rather than primary prevention alone. At the population level, such stratification may also help inform targeted outreach and resource planning, although these applications require prospective validation. Across all three clusters, the next step would be a prospective longitudinal study to evaluate whether cluster membership is stable over time, whether it predicts future depressive symptoms or clinically confirmed depression, and whether subgroup-informed screening improves case detection, referral completion, treatment engagement, or patient outcomes compared with usual screening. Thus, the present findings should be viewed as a basis for designing and testing stratified screening strategies, not as evidence for immediate clinical deployment.

An important methodological advantage of SYNERGY-VAE lies in its ability to capture nonlinear relationships across heterogeneous data modalities an aspect where traditional approaches fall short. Unlike linear dimensionality reduction techniques such as PCA, which assume orthogonality and may overlook cross-domain dependencies, our VAE-based latent space enabled the discovery of clinically meaningful subgroups not visible through PCA-based clustering. Indeed, we experimentally confirmed that PCA followed by K-means clustering yielded weaker cluster separation and no significant association with depression prevalence (Table F in S1 Appendix). Additionally, we compared our architecture against a simplified VAE using a single shared decoder across modalities. This configuration resulted in substantially higher reconstruction loss and poorer subgroup definition, underscoring the importance of modality-specific decoders in capturing nuanced health patterns (S2 Fig, Table 7 in S1 Appendix). Furthermore, pooled classifiers trained on the entire dataset without clustering achieved lower AUCs compared to the cluster-specific models, highlighting that population-wide modeling can obscure critical subgroup signals (Table P in S1 Appendix). These results collectively validate the need for non-linear, multimodal integration and tailored cluster-wise modeling in uncovering latent health phenotypes relevant to depression.

As the prevalence of depression varied across clusters, classifier performance exhibited corresponding variability. The highest area under the curve (AUC) score of 0.83 was attained in Cluster 2, where depression-related signals manifested most strongly, likely due to its enrichment in immuno-metabolic features. Conversely, Cluster 1’s classifier demonstrated slightly reduced performance (AUC = 0.78), implying that some health profiles may encapsulate depression risk in more subtle or nonlinear terms. This variability suggests that mental health risk may be apprehended differently across population strata, reinforcing the necessity for context-aware, subgroup-specific modeling. Clinically, our framework could enhance potential screening framework pending prospective validation within large-scale health systems or community health settings. For instance, individuals categorized into high-risk clusters based on multimodal health data could be prioritized for depression screenings, follow-up evaluations, or tailored interventions. Because our approach uses routinely collected health data, it may be adaptable to existing clinical or public health workflows with appropriate validation and implementation study.

Despite these strengths, several limitations warrant attention. First, NHANES comprises a cross-sectional dataset, thereby constraining causal inference and precluding assessment of temporal trajectories or directionality across clusters. Consequently, the subgroups and risk profiles identified here reflect statistical associations observed at a single time point and should not be interpreted as stable disease phenotypes or clinically validated depression subtypes. Readers situated within precision psychiatry frameworks should note that these clusters are derived from a screening instrument rather than clinically confirmed diagnoses, and their translational applicability remains contingent on prospective longitudinal validation. Second, depression was evaluated using self-report measures (PHQ-9), which, although widely validated, may be susceptible to reporting biases and misclassification relative to clinically confirmed diagnoses. Third, while we incorporated diverse health variables, environmental and contextual exposures (e.g., neighborhood stressors or healthcare access) were not included. Fourth, although PFI provided insights into predictor significance, future research could benefit from the inclusion of counterfactual explanations to further enhance both global and local interpretability. Fifth, encoder weight magnitudes provide a heuristic approximation of feature influence on the latent representation and should not be interpreted as robust global importance scores. Lastly, because descriptive statistics were not conducted using formal survey-weighted methods, results should be interpreted as characterizing the analytic sample with complete multimodal data. The omission of formal survey weights merits acknowledgment: as a complex probability sample, NHANES oversamples certain subgroups, and unweighted analyses may misrepresent population segments in unpredictable ways, particularly for lower-income individuals, racial and ethnic minorities, and older adults. Additionally, restricting the cohort to individuals with complete data across all five modalities likely introduces selection pressures, as individuals with missing data tend to differ systematically from those with complete records. Both constraints limit generalizability beyond the analytic sample and should be considered when interpreting the reported cluster profiles and depression associations. Finally, the SYNERGY-VAE framework was trained and evaluated within a harmonized NHANES 2005–2018 analytic cohort and should not be assumed to transfer directly to other survey or clinical datasets. Application to external datasets would require dataset-specific feature mapping, preprocessing, retraining or recalibration, and independent validation before use for clinical screening, surveillance, or decision support. Future work should also compare participants included in the complete-case analytic cohort with those excluded because of missing multimodal data to better quantify the direction and magnitude of potential selection bias.

Future endeavors could involve extending this framework to longitudinal datasets to enable dynamic modeling of mental health trajectories, while also applying these methods to underserved or high-risk populations to reveal structural disparities that may not be fully captured within NHANES. Furthermore, integrating this model within digital health tools could facilitate scalable, data-driven stratification of mental health within primary care or public health environments.

In conclusion, SYNERGY-VAE illustrates that latent depression-related phenotypes can be discerned from routinely collected, multimodal health data through the application of explainable deep learning techniques. By identifying and interpreting subgroups within a multimodal NHANES analytic sample with distinct biopsychosocial signatures, this framework represents a significant advancement toward more precise and inclusive strategies for mental health prediction and prevention.

## Supporting Information

S1 Appendix Table A. **Architecture summary of the SYNERGY-VAE model.** The model features a shared encoder that maps a 153-dimensional multimodal input into a 3-dimensional latent space via dense layers and a sampling layer. The encoder output feeds into five modality-specific decoders, each responsible for reconstructing one of the five NHANES data domains (demographic, dietary, examination, laboratory, and questionnaire), enabling both integration and modality-preserving reconstruction. Table B. Baseline characteristics of the study population stratified by depression status. Compares demographic, clinical, dietary, examination, laboratory, and questionnaire-based variables between individuals with and without depressive symptoms as defined by PHQ-9 ≥ 10. Statistical significance assessed using t-tests or chi-square tests as appropriate.Table C. Cluster-specific baseline characteristics of the study population. Presents the distribution of demographic, dietary, examination, laboratory, and questionnaire variables across the three latent clusters identified by SYNERGY-VAE, enabling characterization of the distinct health profiles captured within each subgroup. Table D. Mean AUC and 95% confidence intervals from 5-fold cross-validation. Summarizes the predictive performance of four classification models (Logistic Regression, Random Forest, Gradient Boosting, and XGBoost) evaluated within each of the three depression-related clusters, reported as average AUC with 95% confidence intervals based on stratified 5-fold cross-validation. Table E. Performance metrics including precision, recall, sensitivity, and specificity for each machine learning model across the three latent clusters. These metrics complement the ROC-AUC results and provide a granular view of model performance within each subgroup, highlighting variability in depression prediction across distinct health phenotypes. Table F. Silhouette scores for PCA-based K-means clustering. Presents silhouette scores for cluster sizes ranging from k = 2 to 10 derived from applying K-means to the PCA-transformed feature space, demonstrating limited cluster cohesion and separation relative to the SYNERGY-VAE approach. Table G. Cluster-wise distribution of participants and depression prevalence using the single-decoder VAE framework. Summarizes cluster membership, depression case counts, and prevalence for clusters derived from a single shared decoder architecture, showing low and relatively uniform depression prevalence across clusters and no significant enrichment. Table H. Sensitivity analysis of the KL regularization coefficient (λ) on latent space quality and downstream clustering performance. Reports reconstruction loss, KL divergence, ELBO, silhouette score, cluster-level depression enrichment p-values, and mean cluster-specific XGBoost AUC across λ ∈ {0.5, 1.0, 2.0, 4.0}, supporting the selection of λ = 1.0. Table I. Cluster stability assessment across K-means initializations and bootstrap resamples. (a) Adjusted Rand Index (ARI) across 50 random K-means seeds and 100 bootstrap resamples of the full dataset, with full VAE retraining. (b) Cluster size consistency across bootstrap resamples. (c) Consistency of cluster-specific depression prevalence and statistical enrichment significance across bootstrap resamples. Table J. Cluster stability and sensitivity to the number of clusters (k = 2 to 6). (a) Numerical silhouette scores, within-cluster sum of squares, and K-means ARI stability metrics across k = 2 to 6. (b) Sensitivity of depression enrichment and classification utility across candidate cluster numbers, reporting depression prevalence range, mean cluster-specific XGBoost AUC, pooled XGBoost AUC, and AUC uplift for each value of k. Table K. Sensitivity of XGBoost classification performance to PFI feature selection threshold across clusters.Reports AUC with 95% confidence intervals for the top 20, 25, 30 (selected), 35, and 40 PFI-ranked features within each cluster, confirming that the top-30 threshold sits within a stable performance plateau. Table L. Baseline comparator specification and performance comparison. (a) Preprocessing and tuning details for SYNERGY-VAE and all three baseline comparators (PCA + K-means, single-decoder VAE + K-means, and pooled classifier without clustering). (b) Classification performance across all comparators, reported as XGBoost AUC with 95% confidence intervals. (c) Statistical comparison of performance differences, reported as bootstrap ΔAUC with 95% confidence intervals for SYNERGY-VAE versus each comparator. Table M. SHAP-based interpretability analysis and concordance with PFI and encoder weight rankings. (a) Spearman rank correlation between SHAP, PFI, and encoder weight rankings across the top 30 features per cluster, with 95% bootstrap confidence intervals. (b) Feature-level concordance summary showing agreement in top 10 rankings across all three methods, with identification of key discordant features. Table N. Comparison of clustering performance across latent dimensionalities (2–10) in the SYNERGY-VAE framework. Reports silhouette score and within-cluster sum of squares for each latent dimension, supporting the selection of dimension 3 as the optimal balance between cluster cohesion and model complexity. Table O. Predictive performance of pooled models trained on the entire cohort (N = 13,532) without clustering.Reports AUC with 95% confidence intervals for XGBoost, Gradient Boosting, Logistic Regression, and Random Forest under a pooled modeling strategy, demonstrating lower performance relative to cluster-specific models. Table P. Cycle-restricted robustness of clustering results. Presents the number of clusters retained, cluster-specific depression prevalence, and consistency with the main analysis for subsets restricted to early (2005–2010), middle (2011–2014), and late (2015–2018) NHANES cycles, supporting the robustness of the three-cluster solution across survey periods. Table Q. Benjamini-Hochberg adjusted q-values for top SMD-ranked cluster signature features Table R**. Bootstrap comparison of cluster-specific XGBoost models versus pooled XGBoost model Fig A. Flow diagram of analytic cohort derivation across NHANES 2005–2018 cycles.** Shows participant inclusion and exclusion at each survey cycle, sequentially applying criteria for non-missing PHQ-9 availability and completeness across the five modeled domains (demographic, dietary, examination, laboratory, and questionnaire), resulting in the final analytic cohort of 13,532 participants. Fig B. Clustering performance metrics for the single-decoder VAE latent space. (a) Silhouette scores across varying cluster sizes (k = 2 to 15), indicating poor overall cohesion and separation with a maximum score of approximately 0.19 at k = 11. (b) Elbow method plot based on K-means inertia, suggesting diminishing compactness gains beyond k = 8–10. These results demonstrate the suboptimal latent structure produced by the single-decoder model relative to the multi-decoder SYNERGY-VAE. Fig C. Global SHAP feature importance: mean absolute SHAP value for the top 10 features per cluster (XGBoost).Displays cluster-specific SHAP importance profiles for Clusters 1, 2, and 3, illustrating both shared and cluster-specific predictive features and supporting the concordance analysis with PFI and encoder weight rankings reported in Table M in S1 Appendix.
(PDF)
