# Supplementary material for: SYNERGY-VAE: An explainable generative deep learning framework for discovering depression subgroups from multimodal population health data
Source: PLOS Digit Health. 2026 Sep 24;5(9):e0001719. doi: 10.1371/journal.pdig.0001719 (PMC13606994; doi:10.1371/journal.pdig.0001719)

S1 Appendix

Supplementary material for: SYNERGY-VAE: A Generative and Explainable Deep Learning Framework for Multimodal Depression Subgroup Discovery in NHANES Data

Authors: Divya Sharma[1,2,3*], Alice Rueda[4,5], Qiaowei Lin[4], Shakila Meshkat[4], Argyrios Perivolaris[4], Venkat Bhat*[4,5,6]

Affiliations:

Department of Mathematics and Statistics, York University, Toronto, Ontario, Canada
Biostatistics Department, Princess Margaret Cancer Centre, University Health Network, Toronto, Ontario, Canada
Biostatistics Department, Dalla Lana School of Public Health, University of Toronto, Toronto, Ontario, Canada
AI for Mental Health Program, St. Michael's Hospital, Unity Health Toronto, Toronto, Ontario, Canada
Department of Electrical and Computer Engineering, Toronto Metropolitan University, Toronto, Ontario, Canada
Department of Psychiatry, University of Toronto, Toronto, Ontario, Canada

*Co-Corresponding Authors

Table of Contents **Page**

List of Authors ---1
Table of contents ---1
Table A ---2
Table B --- 3
Table C --- 11
Table D --- 20
Table E --- 20
Table F ---21
Table G --- 21
Table H --- 22
Table I --- 22
Table J ---22
Table K ---23
Table L ---23
Table M ---25
Table N ---25
Table O ---26
Table P ---26
Table Q ---26
Table R ---27
Fig A --- 28
Fig B --- 29
Fig C --- 29

1. Model Architecture and Training Details

SYNERGY-VAE was implemented using TensorFlow/Keras. The model consists of a shared encoder and five modality-specific decoders (Table A). The encoder comprises two fully connected layers, and each decoder contains one hidden layer followed by an output layer matching the original dimensionality of each modality. A latent dimensionality of 3 was selected based on clustering stability and interpretability. We trained the model for 400 epochs using the Adam optimizer with a learning rate of 0.0001, batch size of 128, and binary cross-entropy loss for each modality's reconstruction. KL divergence was weighted equally with reconstruction loss using a λ -VAE formulation ($\lambda = 1$). Early stopping was employed based on validation loss with a patience of 10 epochs to prevent overfitting. Random seed was set to ensure reproducibility of clustering outcomes. All models were trained on an NVIDIA A100 GPU.

Table A: Architecture summary of the SYNERGY-VAE model. The model features a shared encoder that maps a 153-dimensional multimodal input into a 3-dimensional latent space via dense layers and a sampling layer. The encoder output feeds into five modality-specific decoders (functional_21 to functional_25), each responsible for reconstructing one of the five NHANES data domains, demographic, dietary, examination, laboratory, and questionnaire data, enabling both integration and modality-preserving reconstruction.

Layer (type)	Output Shape	Param #	Connected to
input_layer (InputLayer)	(None, 153)	0	-
dense (Dense)	(None, 64)	9,856	input_layer[0][0]
dense_1 (Dense)	(None, 3)	195	dense[0][0]
dense_2 (Dense)	(None, 3)	195	dense[0][0]
lambda (Lambda)	(None, 3)	0	dense_1[0][0], dense_2[0][0]
functional_21 (Functional)	(None, 19)	1,491	lambda[0][0]
functional_22 (Functional)	(None, 10)	906	lambda[0][0]
functional_23 (Functional)	(None, 13)	1,101	lambda[0][0]
functional_24 (Functional)	(None, 63)	4,351	lambda[0][0]
functional_25 (Functional)	(None, 48)	3,376	lambda[0][0]

A. Stepwise analytical workflow of SYNERGY-VAE

Input: Multimodal NHANES analytic cohort with depression label

STAGE 1: UNSUPERVISED PHENOTYPING (Full Cohort)
1. Fit SYNERGY-VAE on full analytic cohort
2. Obtain latent embeddings for all participants
3. Fit K-means on latent embeddings
4. Assign cluster labels to all participants

STAGE 2: SUPERVISED PREDICTION (Per Cluster) Fully leakage-controlled

For each cluster c:
a. Subset participants assigned to cluster c
b. Split data into stratified train/test sets

c. On training set only:
i. Compute PFI-based feature ranking
ii. Select top features
iii. Apply SMOTE
iv. Tune model hyperparameters with stratified 5-fold CV
v. Train final classifier
d. Evaluate classifier on held-out test set only

Output: Cluster-specific predictive performance and feature importance

Table B: Baseline characteristics of the study population stratified by depression status. The table compares demographic, clinical, dietary, examination, laboratory, and questionnaire-based variables between individuals with and without depressive symptoms, as defined by PHQ-9 criteria. Statistical significance for group differences was assessed using appropriate tests (e.g., t-test or chi-square), with corresponding p-values reported.

Characteristic	Total	Depressive Symptoms No	Depressive Symptoms Yes	*p*-value
Sample Size	13532	12304	1228	-
Total PHQ-9 Scores (mean (SD))	3.28 (4.38)	2.17 (2.44)	14.39 (3.99)	< 0.001
1. Demographic				
Age in years at screening (mean (SD))	45.95 (18.18)	45.93 (18.35)	46.15 (16.35)	0.686
Total # of people in the Household (mean (SD))	3.28 (1.72)	3.28 (1.72)	3.23 (1.77)	0.311
Total # of people in the Family (mean (SD))	3.06 (1.77)	3.07 (1.77)	2.96 (1.83)	0.057
Ratio of family income to poverty (mean (SD))	2.44 (1.65)	2.52 (1.65)	1.60 (1.33)	< 0.001
Sex (%)				< 0.001
Female	6784 (50.1)	6013 (48.9)	771 (62.8)	
Male	6748 (49.9)	6291 (51.1)	457 (37.2)	
Race/Hispanic origin (%)				< 0.001
Mexican American	2139 (15.8)	1945 (15.8)	194 (15.8)	
Non-Hispanic Black	2981 (22.0)	2706 (22.0)	275 (22.4)	
Non-Hispanic White	5597 (41.4)	5098 (41.4)	499 (40.6)	
Other Hispanic	1351 (10.0)	1184 (9.6)	167 (13.6)	
Other Race - Including Multi-Racial	1464 (10.8)	1371 (11.1)	93 (7.6)	
Citizenship status (%)				0.279
Citizen by birth or naturalization	11511 (85.1)	10442 (84.9)	1069 (87.1)	
Not a citizen of the US	1990 (14.7)	1835 (14.9)	155 (12.6)	
Don't know	4 (0.0)	4 (0.0)	0 (0.0)	
Refused	22 (0.2)	19 (0.2)	3 (0.2)	
Language of SP Interview (%)				0.006
English	11862 (87.7)	10816 (87.9)	1046 (85.2)	
Spanish	1670 (12.3)	1488 (12.1)	182 (14.8)	
Proxy used in SP Interview (%)				0.706
No	13480 (99.6)	12258 (99.6)	1222 (99.5)	
Yes	52 (0.4)	46 (0.4)	6 (0.5)	
Interpreter used in SP Interview (%)				0.179
No	13221 (97.7)	12014 (97.6)	1207 (98.3)	
Yes	311 (2.3)	290 (2.4)	21 (1.7)	
Language of Family Interview (%)				0.080
English	12304 (90.9)	11209 (91.1)	1095 (89.2)	
Spanish	1083 (8.0)	966 (7.9)	117 (9.5)	

Proxy used in Family Interview (%)<br>No<br>Yes	 13381 (98.9)<br>6 (0.0)	 12169 (98.9)<br>6 (0.0)	 1212 (98.7)<br>0 (0.0)	0.528
Interpreter used in Family Interview (%)<br>No<br>Yes	 13133 (97.1)<br>254 (1.9)	 11936 (97.0)<br>239 (1.9)	 1197 (97.5)<br>15 (1.2)	0.150
Language of MEC Interview (%)<br>No<br>Yes	 12264 (90.6)<br>1268 (9.4)	 11163 (90.7)<br>1141 (9.3)	 1101 (89.7)<br>127 (10.3)	0.240
Interpreter used in MEC Interview (%)<br>No<br>Yes	 13188 (97.5)<br>344 (2.5)	 11994 (97.5)<br>310 (2.5)	 1194 (97.2)<br>34 (2.8)	0.664
2. Dietary				
Total # of Dietary Supplements taken (mean (SD))	1.12 (2.18)	1.13 (2.11)	1.05 (2.74)	0.225
Total # of Antacids Taken (mean (SD))	0.14 (1.56)	0.13 (1.48)	0.22 (2.23)	0.080
Dietary Supplements taken (%)<br>No<br>Yes<br>Refused<br>Don't know	 7095 (52.4)<br>6432 (47.5)<br>2 (0.0)<br>1 (0.0)	 6423 (52.2)<br>5877 (47.8)<br>1 (0.0)<br>1 (0.0)	 672 (54.7)<br>555 (45.2)<br>1 (0.0)<br>0 (0.0)	0.123
Antacids taken (%)<br>No<br>Yes<br>Refused<br>Don't know	 12002 (88.7)<br>1525 (11.3)<br>2 (0.0)<br>2 (0.0)	 10956 (89.0)<br>1344 (10.9)<br>1 (0.0)<br>2 (0.0)	 1046 (85.2)<br>181 (14.7)<br>1 (0.1)<br>0 (0.0)	<0.001
Number of days of intake (%)<br>Day 1 only<br>Day 1 and day 2	 1535 (11.3)<br>11579 (85.6)	 1386 (11.3)<br>10541 (85.7)	 149 (12.1)<br>41 (3.3)	0.552
Intake day of the week (%)<br>Monday<br>Tuesday<br>Wednesday<br>Thursday<br>Friday<br>Saturday<br>Sunday	 1058 (7.8)<br>1243 (9.2)<br>1222 (9.0)<br>1460 (10.8)<br>3066 (22.7)<br>2870 (21.2)<br>2250 (16.6)	 941 (7.6)<br>1128 (9.2)<br>1109 (9.0)<br>1323 (10.8)<br>2809 (22.8)<br>2629 (21.4)<br>2039 (16.6)	 117 (9.5)<br>115 (9.4)<br>113 (9.2)<br>137 (11.2)<br>257 (20.9)<br>241 (19.6)<br>211 (17.2)	0.220
Language respondent used mostly - day one (%)<br>English<br>Spanish<br>English and Spanish<br>Asian Languages<br>Asian Languages and English<br>Other	 11385 (84.1)<br>1517 (11.2)<br>149 (1.1)<br>50 (0.4)<br>30 (0.2)<br>40 (0.3)	 10378 (84.3)<br>1352 (11.0)<br>136 (1.1)<br>47 (0.4)<br>29 (0.2)<br>38 (0.3)	 1007 (82.0)<br>165 (13.4)<br>13 (1.1)<br>3 (0.2)<br>1 (0.1)<br>2 (0.2)	0.129
Language respondent used mostly- two-day (%)<br>English<br>Spanish<br>English and Spanish<br>Asian Languages<br>Asian Languages and English<br>Other	 10193 (75.3)<br>1302 (9.6)<br>86 (0.6)<br>62 (0.5)<br>2 (0.0)<br>5 (0.0)	 9295 (75.5)<br>1163 (9.5)<br>78 (0.6)<br>61 (0.5)<br>2 (0.0)<br>5 (0.0)	 898 (73.1)<br>139 (11.3)<br>8 (0.7)<br>1 (0.1)<br>0 (0.0)<br>0 (0.0)	0.101
3. Examination				
Blood pressure: 60 sec. pulse (mean (SD))	72.73 (11.96)	72.41 (11.82)	75.91 (12.80)	<0.001

Blood pressure: maximum inflation levels (mm Hg) (mean (SD))	149.29 (23.26)	149.20 (20.53)	150.20 (41.74)	0.156
Systolic: Blood pres (1st rdg) (mm Hg) (mean (SD))	122.89 (18.33)	122.95 (18.18)	122.30 (19.77)	0.255
Diastolic: Blood pres (1st rdg) (mm Hg) (mean (SD))	70.11 (13.09)	70.03 (13.08)	70.95 (13.10)	0.023
Systolic: Blood pres (2nd rdg) (mm Hg) (mean (SD))	122.22 (18.12)	122.25 (17.93)	121.94 (19.96)	0.566
Diastolic: Blood pres (2nd rdg) (mm Hg) (mean (SD))	69.77 (13.12)	69.68 (13.06)	70.66 (13.66)	0.015
Body Measures: Standing Height (cm) (mean (SD))	167.63 (10.13)	167.84 (10.13)	165.49 (9.90)	<0.001
Body Mass Index (kg/m^2) (mean (SD))	28.96 (7.00)	28.79 (6.80)	30.68 (8.54)	<0.001
Upper Leg Length (cm) (mean (SD))	38.79 (3.84)	38.88 (3.82)	37.87 (3.98)	<0.001
Upper Arm Length (cm) (mean (SD))	37.23 (2.82)	37.25 (2.82)	36.93 (2.77)	<0.001
Arm Circumference (cm) (mean (SD))	33.10 (5.14)	33.03 (5.07)	33.73 (5.75)	<0.001
Waist Circumference (cm) (mean (SD))	98.29 (16.63)	97.97 (16.40)	101.54 (18.47)	<0.001
Blood pressure: Pulse regular or irregular (%)<br>Regular<br>Irregular	 12943 (95.6)<br>325 (2.4)	 11772 (95.7)<br>296 (2.4)	 1171 (95.4)<br>29 (2.4)	0.680
4. Laboratory				
Albumin, urine (ug/mL) (mean (SD))	36.72 (222.03)	34.53 (212.98)	58.64 (297.10)	<0.001
Creatinine, urine (umol/L) (mean (SD))	11077.58 (7247.52)	11005.46 (7169.90)	11800.05 (7950.79)	<0.001
Cadmium (nmol/L) (mean (SD))	4.48 (5.17)	4.31 (5.00)	6.19 (6.35)	<0.001
Lead (umol/L) (mean (SD))	0.07 (0.09)	0.07 (0.09)	0.07 (0.07)	0.451
Mercury, total (nmol/L) (mean (SD))	7.69 (12.70)	7.88 (12.87)	5.80 (10.67)	<0.001
Direct HDL-Cholesterol (mmol/L) (mean (SD))	1.36 (0.41)	1.37 (0.41)	1.31 (0.40)	<0.001
Total Cholesterol (mmol/L) (mean (SD))	4.96 (1.08)	4.96 (1.08)	5.02 (1.16)	0.058
White blood cell count (1000 cells/uL) (mean (SD))	7.23 (4.16)	7.18 (4.30)	7.64 (2.36)	<0.001
Lymphocyte percent (%) (mean (SD))	30.67 (8.53)	30.75 (8.51)	29.87 (8.74)	0.001
Monocyte percent (%) (mean (SD))	7.82 (2.42)	7.85 (2.41)	7.48 (2.48)	<0.001
Segmented neutrophils percent (%) (mean (SD))	57.99 (9.58)	57.86 (9.54)	59.26 (9.94)	<0.001
Eosinophils percent (%) (mean (SD))	2.85 (2.12)	2.86 (2.14)	2.74 (1.83)	0.064
Basophils percent (%) (mean (SD))	0.72 (0.55)	0.72 (0.55)	0.69 (0.50)	0.076
Lymphocyte number (1000 cells/uL) (mean (SD))	2.17 (3.24)	2.17 (3.39)	2.21 (0.82)	0.645
Monocyte number (1000 cells/uL) (mean (SD))	0.55 (0.20)	0.55 (0.20)	0.55 (0.20)	0.518
Segmented neutrophils num (1000 cell/uL) (mean (SD))	4.26 (1.87)	4.22 (1.86)	4.63 (1.93)	<0.001
Eosinophils number (1000 cells/uL) (mean (SD))	0.20 (0.19)	0.20 (0.19)	0.20 (0.15)	0.954
Basophils number (1000 cells/uL) (mean (SD))	0.04 (0.07)	0.04 (0.07)	0.05 (0.06)	0.588
Red blood cell count (million cells/uL) (mean (SD))	4.65 (0.50)	4.66 (0.50)	4.57 (0.49)	<0.001
Hemoglobin (g/dL) (mean (SD))	14.07 (1.50)	14.09 (1.50)	13.85 (1.58)	<0.001
Mean cell volume (fL) (mean (SD))	89.26 (5.86)	89.25 (5.87)	89.35 (5.74)	0.576
Mean cell hemoglobin (pg) (mean (SD))	30.33 (2.40)	30.33 (2.39)	30.39 (2.49)	0.360
Mean cell hemoglobin concentration (g/dL) (mean (SD))	33.96 (1.05)	33.96 (1.04)	33.98 (1.08)	0.597
Red cell distribution width (%) (mean (SD))	13.15 (1.35)	13.13 (1.33)	13.40 (1.60)	<0.001

Platelet count SI (1000 cells/uL) (mean (SD))	243.45<br>(63.30)	242.70 (62.61)	251.08 (69.42)	<0.001
Mean platelet volume (fL) (mean (SD))	8.21 (0.94)	8.21 (0.93)	8.23 (1.00)	0.595
Cotinine (ng/mL) (mean (SD))	55.54<br>(124.55)	51.38 (120.72)	97.62 (151.97)	<0.001
Glycohemoglobin (%) (mean (SD))	5.71 (1.06)	5.69 (1.01)	5.91 (1.44)	<0.001
Mercury, inorganic (umol/L) (mean (SD))	1.43 (2.35)	1.41 (1.33)	1.63 (6.59)	0.002
Albumin (g/dL) (mean (SD))	4.27 (0.35)	4.27 (0.35)	4.18 (0.36)	<0.001
Alanine aminotransferase ALT (U/L) (mean (SD))	25.62 (22.51)	25.50 (19.09)	26.84 (44.11)	0.054
Aspartate aminotransferase AST (U/L) (mean (SD))	25.94 (17.89)	25.79 (15.54)	27.48 (33.37)	0.002
Alkaline phosphatase (U/L) (mean (SD))	69.53 (23.81)	69.20 (23.73)	72.88 (24.41)	<0.001
Blood urea nitrogen (mmol/L) (mean (SD))	4.65 (1.99)	4.66 (1.96)	4.51 (2.23)	0.013
Total calcium (mmol/L) (mean (SD))	2.36 (0.09)	2.36 (0.09)	2.35 (0.10)	0.013
Cholesterol (mmol/L) (mean (SD))	4.96 (1.09)	4.95 (1.08)	5.00 (1.17)	0.190
Bicarbonate (mmol/L) (mean (SD))	25.14 (2.24)	25.16 (2.22)	24.90 (2.37)	<0.001
Creatinine (umol/L) (mean (SD))	79.09 (37.61)	78.94 (33.46)	80.64 (66.36)	0.145
Gamma glutamyl transferase (U/L) (mean (SD))	28.20 (39.61)	27.70 (38.04)	33.32 (52.72)	<0.001
Glucose, serum (mmol/L) (mean (SD))	5.61 (2.12)	5.56 (1.98)	6.05 (3.17)	<0.001
Iron, refrigerated (umol/L) (mean (SD))	15.24 (6.37)	15.32 (6.36)	14.44 (6.41)	<0.001
Lactate dehydrogenase LDH (U/L) (mean (SD))	130.59<br>(35.52)	130.52 (34.81)	131.34 (42.06)	0.454
Phosphorus (mmol/L) (mean (SD))	1.21 (0.19)	1.21 (0.18)	1.23 (0.20)	0.016
Bilirubin, total (umol/L) (mean (SD))	11.88 (5.33)	11.98 (5.34)	10.95 (5.10)	<0.001
Total protein (g/dL) (mean (SD))	7.17 (0.48)	7.17 (0.47)	7.16 (0.52)	0.470
Triglycerides (mmol/L) (mean (SD))	1.71 (1.54)	1.69 (1.49)	1.90 (1.94)	<0.001
Uric acid (umol/L) (mean (SD))	322.76<br>(84.59)	323.25 (84.09)	317.87 (89.35)	0.040
Sodium (mmol/L) (mean (SD))	139.21 (2.27)	139.23 (2.23)	138.97 (2.58)	<0.001
Potassium (mmol/L) (mean (SD))	3.97 (0.34)	3.97 (0.34)	3.96 (0.36)	0.496
Chloride (mmol/L) (mean (SD))	103.74 (2.89)	103.75 (2.86)	103.64 (3.24)	0.198
Osmolality (mmol/Kg) (mean (SD))	278.11 (5.08)	278.12 (5.01)	277.97 (5.69)	0.329
Globulin (g/L) (mean (SD))	29.07 (4.57)	29.00 (4.49)	29.78 (5.28)	<0.001
Mercury, total comment code (%)<br>At or above the detection limit<br>Below lower detection limit	 11675 (86.3)<br>1368 (10.1)	 10641 (89.7)<br>1225 (10.3)	 1034 (87.9)<br>143 (12.1)	0.057
Fasting: Coffee or tea with cream or sugar? (%)<br>No<br>Yes	 13254 (97.9)<br>147 (1.1)	 12056 (98.9)<br>135 (1.1)	 1198 (99.0)<br>12 (1.0)	0.823
Fasting: Alcohol, such as beer, wine, or liquor? (%)<br>No<br>Yes	 13349 (98.6)<br>52 (0.4)	 12142 (99.6)<br>49 (0.4)	 1207 (99.8)<br>3 (0.2)	0.562
Fasting: Gum, breath mints, lozenges or cough drops, or other cough or cold remedies? (%)<br>No<br>Yes	 <br>13024 (96.2)<br>377 (2.8)	 <br>11857 (97.3)<br>334 (2.7)	 <br>1167 (96.4)<br>43 (3.6)	0.123
Fasting: Antacids, laxatives, or anti-diarrheals? (%)<br>No<br>Yes	 13346 (98.6)<br>55 (0.4)	 12141 (99.6)<br>50 (0.4)	 1205 (99.6)<br>5 (0.4)	1.000
Fasting: Dietary supplements such as vitamins and minerals? (%)<br>No<br>Yes	 13289 (98.2)<br>112 (0.8)	 12091 (99.2)<br>100 (0.8)	 1198 (99.0)<br>12 (1.0)	0.646

Session in which SP was examined (%)<br>Afternoon<br>Evening<br>Morning	 4890 (36.1)<br>2148 (15.9)<br>6494 (48.0)	 4416 (35.9)<br>1946 (15.8)<br>5942 (48.3)	 474 (38.6)<br>202 (16.4)<br>552 (45.0)	0.076
Hepatitis A Antibody (Anti-HAV) (%)<br>Indeterminate<br>Negative<br>Positive	 13 (0.1)<br>6946 (51.3)<br>5710 (42.2)	 11 (0.1)<br>6301 (54.6)<br>5230 (45.3)	 2 (0.2)<br>645 (57.2)<br>480 (42.6)	0.160
Hepatitis B Surface Antibody (%)<br>Negative<br>Positive	 9357 (69.1)<br>3527 (26.1)	 8480 (72.3)<br>3243 (27.7)	 877 (75.5)<br>284 (24.5)	0.021
Hepatitis B core antibody (%)<br>Negative<br>Positive	 12020 (88.8)<br>866 (6.4)	 10952 (93.4)<br>773 (6.6)	 1068 (92.0)<br>93 (8.0)	0.075
Mercury, inorganic comment code (%)<br>At or above the detection limit<br>Below lower detection limit	 2857 (21.1)<br>10165 (75.1)	 2633 (22.2)<br>9215 (77.8)	 224 (19.1)<br>950 (80.9)	0.014
5. Questionnaire				
Number of rooms in home<br>(mean (SD))	10.08 (2.85)	10.09 (2.88)	9.93 (2.46)	0.057
Current self-reported height (inches) (mean (SD))	201.37<br>(1148.87)	201.10<br>(1147.31)	204.13<br>(1164.85)	0.930
Current self-reported weight (pounds) (mean (SD))	279.19<br>(983.05)	277.08 (975.64)	300.35<br>(1054.89)	0.431
Self-reported weight - 1 yr ago (pounds) (mean (SD))	318.85<br>(1158.24)	307.54<br>(1113.85)	432.47<br>(1530.66)	<0.001
Self-reported greatest weight (pounds) (mean (SD))	313.59<br>(1065.36)	307.93<br>(1045.20)	370.32<br>(1248.59)	0.051
Self-reported greatest weight (pounds) (mean (SD))	560.38<br>(7182.72)	570.85<br>(7252.95)	454.71<br>(6433.61)	0.593
Ever told you had high blood pressure (%)<br>No<br>Yes<br>Don't know	 9181 (67.8)<br>4331 (32.0)<br>20 (0.1)	 8505 (69.1)<br>3779 (30.7)<br>20 (0.2)	 676 (55.0)<br>552 (45.0)<br>0 (0.0)	<0.001
Current Health Status: General health condition (%)<br>Excellent<br>Very good<br>Good<br>Fair<br>Poor	 1305 (9.7)<br>3645 (26.9)<br>5441 (40.2)<br>2666 (19.7)<br>475 (3.5)	 1278 (10.4)<br>3533 (28.7)<br>5061 (41.1)<br>2184 (17.8)<br>248 (2.0)	 27 (2.2)<br>112 (9.1)<br>380 (30.9)<br>482 (39.3)<br>227 (18.5)	<0.001
SP have head cold or chest cold (%)<br>No<br>Yes<br>Don't know	 11266 (83.3)<br>2250 (16.6)<br>16 (0.1)	 10312 (83.8)<br>1977 (16.1)<br>15 (0.1)	 954 (77.7)<br>273 (22.2)<br>1 (0.1)	<0.001
SP have stomach or intestinal illness (%)<br>No<br>Yes<br>Don't know	 12535 (92.6)<br>988 (7.3)<br>9 (0.1)	 11508 (93.5)<br>789 (6.4)<br>7 (0.1)	 1027 (83.6)<br>199 (16.2)<br>2 (0.2)	<0.001
SP have flu, pneumonia, ear infection (%)<br>No<br>Yes<br>Don't know	 12961 (95.8)<br>553 (4.1)<br>18 (0.1)	 11842 (96.2)<br>448 (3.6)<br>14 (0.1)	 1119 (91.1)<br>105 (8.6)<br>4 (0.3)	<0.001
SP donated blood in past 12 months (%)<br>No<br>Yes<br>Don't know	 12870 (95.1)<br>654 (4.8)<br>8 (0.1)	 11691 (95.0)<br>607 (4.9)<br>6 (0.0)	 1179 (96.0)<br>47 (3.8)<br>2 (0.2)	0.067

Blood ever tested for HIV virus (%)				<0.001
No	8059 (59.6)	7454 (60.6)	605 (49.3)	
Yes	5037 (37.2)	4456 (36.2)	581 (47.3)	
Don't know	436 (3.2)	394 (3.2)	42 (3.4)	
Doctor told you have diabetes (%)				<0.001
No	11710 (86.5)	10754 (87.4)	956 (77.9)	
Yes	1528 (11.3)	1289 (10.5)	239 (19.5)	
Borderline	287 (2.1)	257 (2.1)	30 (2.4)	
Don't know	7 (0.1)	4 (0.0)	3 (0.2)	
Taking insulin now (%)				<0.001
No	13096 (96.8)	11936 (97.0)	1160 (94.5)	
Yes	434 (3.2)	366 (3.0)	68 (5.5)	
Don't know	1 (0.0)	1 (0.0)	0 (0.0)	
How healthy is the diet (%)				<0.001
Excellent	1144 (8.5)	1094 (8.9)	50 (4.1)	
Very good	2619 (19.4)	2487 (20.2)	132 (10.7)	
Good	5630 (41.6)	5226 (42.5)	404 (32.9)	
Fair	3336 (24.7)	2888 (23.5)	448 (36.5)	
Poor	795 (5.9)	602 (4.9)	193 (15.7)	
Don't know	8 (0.1)	7 (0.1)	1 (0.1)	
Past 30 day milk product consumption (%)				0.026
Never	2325 (17.2)	2102 (17.1)	223 (18.2)	
Rarely-less than once a week	2297 (17.0)	2075 (16.9)	222 (18.1)	
Sometimes-less than once a day	3877 (28.7)	3533 (28.8)	344 (28)	
Often-once a day or more	4998 (36.9)	4565 (37.1)	433 (35.3)	
Varied	35 (0.3)	29 (0.2)	6 (0.5)	
HH Worried run out of food (%)				<0.001
Never true	9466 (70.0)	8884 (72.2)	582 (47.4)	
Sometimes true	2555 (18.9)	2197 (17.9)	358 (29.2)	
Often true	1366 (10.1)	1099 (8.9)	267 (21.7)	
Don't know	15 (0.1)	12 (0.1)	3 (0.2)	
Refused	20 (0.1)	16 (0.1)	4 (0.3)	
HH Food didn't last (%)				<0.001
Never true	10047 (74.2)	9414 (76.5)	633 (51.5)	
Sometimes true	2309 (17.1)	1985 (16.1)	324 (26.4)	
Often true	1040 (7.7)	789 (6.4)	251 (20.4)	
Don't know	7 (0.1)	5 (0.0)	2 (0.2)	
Refused	19 (0.1)	15 (0.1)	4 (0.3)	
HH Couldn't afford balanced meals (%)				<0.001
Never true	10658 (78.8)	9952 (80.9)	706 (57.5)	
Sometimes true	1858 (13.7)	1574 (12.8)	284 (23.1)	
Often true	879 (6.5)	659 (5.4)	220 (17.9)	
Don't know	9 (0.1)	8 (0.1)	1 (0.1)	
Refused	18 (0.1)	15 (0.1)	3 (0.2)	
Household food security category (%)				<0.001
HH full food security: 0	8953 (66.2)	8443 (68.6)	510 (41.5)	
HH marginal food security: 1-2	1606 (11.9)	1429 (11.6)	177 (14.4)	
HH low food security: 3-5 / 3-7 (HH with child)	1730 (12.8)	1477 (12)	253 (20.6)	
HH very low food security: 6-10 / 8-18 (HH with child)	1110 (8.2)	841 (6.8)	269 (21.9)	
Adult food security category (%)				<0.001
AD full food security: 0	8996 (66.5)	8480 (68.9)	516 (42.0)	
AD marginal food security: 1-2	1680 (12.4)	1498 (12.2)	182 (14.8)	
AD low food security: 3-5	1502 (11.1)	1269 (10.3)	233 (19.0)	
AD very low food security: 6-10	1221 (9.0)	943 (7.7)	278 (22.6)	
HH Emergency food received (%)				<0.001
No	11899 (87.9)	11011 (89.5)	888 (72.3)	
Yes	1492 (11.0)	1172 (9.5)	320 (26.1)	

Don’t know	15 (0.1)	11 (0.1)	4 (0.3)	
Refused	16 (0.1)	14 (0.1)	2 (0.2)	
Covered by health insurance (%)				
No	3294 (24.3)	2940 (23.9)	354 (28.8)	
Yes	10220 (75.5)	9346 (76.0)	874 (71.2)	
Don’t know	15 (0.1)	15 (0.1)	0 (0.0)	
Refused	2 (0.0)	2 (0.0)	0 (0.0)	
Hospital Utilization & Access to Care: General health				<0.001
condition (%)	1801 (13.3)	1762 (14.3)	39 (3.2)	
Excellent	3637 (26.9)	3509 (28.5)	128 (10.4)	
Very good	5035 (37.2)	4656 (37.8)	379 (30.9)	
Good	2482 (18.3)	2017 (16.4)	465 (37.9)	
Fair	572 (4.2)	356 (2.9)	216 (17.6)	
Poor	4 (0.0)	4 (0.0)	0 (0.0)	
Don’t know	1 (0.0)	0 (0.0)	1 (0.1)	
Refused				
Health now compared with 1 year ago (%)				<0.001
About the same	9329 (68.9)	8675 (70.5)	654 (53.3)	
Better	2678 (19.8)	2474 (20.1)	204 (16.6)	
Worse	1522 (11.2)	1153 (9.4)	369 (30.0)	
Don’t know	3 (0.0)	2 (0.0)	1 (0.1)	
Routine place to go for healthcare (%)				0.003
Yes	11106 (82.1)	10073 (81.9)	1033 (84.1)	
No place	2348 (17.4)	2167 (17.6)	181 (14.7)	
More than one place	76 (0.6)	62 (0.5)	14 (1.1)	
Don’t know	2 (0.0)	2 (0.0)	0 (0.0)	
Overnite hospital patient in last year (%)				<0.001
No	11934 (88.2)	11002 (89.4)	932 (75.9)	
Yes	1593 (11.8)	1298 (10.5)	295 (24.0)	
Don’t know	5 (0.0)	4 (0.0)	1 (0.1)	
Seen mental health professional/past yr (%)				<0.001
No	12416 (91.8)	11509 (93.5)	907 (73.9)	
Yes	1115 (8.2)	794 (6.5)	321 (26.1)	
Don’t know	1 (0.0)	1 (0.0)	0 (0.0)	
Home owned, bought, rented, other (%)				<0.001
Owned or being bought	7836 (57.9)	7287 (59.2)	549 (44.7)	
Other arrangement	397 (2.9)	356 (2.9)	41 (3.3)	
Rented	5174 (38.2)	4550 (37.0)	624 (50.8)	
Don’t know	5 (0.0)	5 (0.0)	0 (0.0)	
Refused	4 (0.0)	4 (0.0)	0 (0.0)	
Received hepatitis A vaccine (%)				0.612
No doses	7958 (58.8)	7234 (58.8)	724 (59.0)	
Less than 2 doses	346 (2.6)	309 (2.5)	37 (3.0)	
Yes, at least 2 doses	3331 (24.6)	3039 (24.7)	292 (23.8)	
Don’t know	1886 (13.9)	1711 (13.9)	175 (14.3)	
Refused				
Received hepatitis B 3 dose series (%)				0.541
No doses	7237 (53.5)	6562 (53.3)	675 (55.0)	
Less than 3 doses	342 (2.5)	309 (2.5)	33 (2.7)	
Yes, at least 3 doses	2371 (17.5)	2170 (17.6)	201 (16.4)	
Yes, all 3 doses	1878 (13.9)	1719 (14.0)	159 (12.9)	
Don’t know	1692 (12.5)	1532 (12.5)	160 (13.0)	
Refused	12 (0.1)	12 (0.1)	0 (0.0)	
Ever been told you have asthma (%)				<0.001
No	11481 (84.8)	10565 (85.9)	916 (74.6)	
Yes	2039 (15.1)	1727 (14.0)	312 (25.4)	
Don’t know	12 (0.1)	12 (0.1)	0 (0.0)	

Taking treatment for anemia/past 3 mos (%)				<0.001
No	12987 (96.0)	11861 (96.4)	1126 (91.7)	
Yes	533 (3.9)	435 (3.5)	98 (8.0)	
Don't know	12 (0.1)	8 (0.1)	4 (0.3)	
Doctor ever said you were overweight (%)				<0.001
No	8926 (66.0)	8285 (67.3)	641 (52.2)	
Yes	4598 (34.0)	4011 (32.6)	587 (47.8)	
Don't know	8 (0.1)	8 (0.1)	0 (0.0)	
Ever receive blood transfusion (%)				<0.001
No	11940 (88.2)	10938 (88.9)	1002 (81.6)	
Yes	1446 (10.7)	1241 (10.1)	205 (16.7)	
Don't know	144 (1.1)	124 (1.0)	20 (1.6)	
Refused	2 (0.0)	1 (0.0)	1 (0.1)	
Type of work done last week (%)				<0.001
Working at a job or business	7351 (54.3)	6969 (56.6)	382 (31.1)	
With a job or business but not at work	274 (2.0)	260 (2.1)	14 (1.1)	
Not working at a job or business				
Looking for work	5285 (39.1)	4526 (36.8)	759 (61.8)	
Refused	615 (4.5)	542 (4.4)	73 (5.9)	
Don't know	1 (0.0)	1 (0.0)	0 (0.0)	
	5 (0.0)	5 (0.0)	0 (0.0)	
Kind of work you have done the longest (%)				<0.001
Same as current occupation	5295 (39.1)	5035 (40.9)	260 (21.2)	
Enter occupation	7540 (55.7)	6657 (54.1)	883 (71.9)	
Armed forces	95 (0.7)	85 (0.7)	10 (0.8)	
Never worked	581 (4.3)	509 (4.1)	72 (5.9)	
Refused	14 (0.1)	12 (0.1)	2 (0.2)	
Don't know	6 (0.0)	5 (0.0)	1 (0.1)	
Products used in home to control insects (%)				<0.001
No	12186 (90.1)	11162 (90.7)	1024 (83.4)	
Yes	1310 (9.7)	1111 (9.0)	199 (16.2)	
Don't know	36 (0.3)	31 (0.3)	5 (0.4)	
Products used to kill weeds? (%)				0.207
No	12694 (93.8)	11162 (90.7)	1024 (83.4)	
Yes	681 (5.0)	1111 (9.0)	199 (16.2)	
Don't know	157 (1.2)	31 (0.3)	5 (0.4)	
Taken prescription medicine, past month (%)				0.207
No	6227 (46.0)	5856 (47.6)	371 (30.2)	
Yes	7302 (54.0)	6445 (52.4)	857 (69.8)	
Refused	2 (0.0)	2 (0.0)	0 (0.0)	
Ever told doctor had trouble sleeping (%)				<0.001
No	10226 (75.6)	9699 (78.8)	527 (42.9)	
Yes	3304 (24.4)	2603 (21.2)	701 (57.1)	
Don't know	2 (0.0)	2 (0.0)	0 (0.0)	
Questionnaire Mode Flag (%)				0.001
A-CASI (12 - 19 Yrs)	773 (5.7)	723 (5.9)	50 (4.1)	
Home Interview (18+ Yrs)	3762 (27.8)	3457 (28.1)	305 (24.8)	
Home Interview (20+ Yrs)	8997 (66.5)	8124 (66.0)	873 (71.1)	
How do you consider your weight (%)				<0.001
About the right weight	5723 (42.3)	5361 (43.6)	362 (29.5)	
Overweight	7039 (52.0)	6289 (51.1)	750 (61.1)	
Underweight	739 (5.5)	626 (5.1)	113 (9.2)	
Refused	3 (0.0)	3 (0.0)	0 (0.0)	
Don't know	27 (0.2)	24 (0.2)	3 (0.2)	
Like to weigh more, less or same (%)				<0.001
Stay about the same	4114 (30.4)	3840 (31.2)	274 (22.3)	
Less	8253	7441 (60.5)	812 (66.1)	
More	(61.0)	1016 (8.3)	140 (11.4)	

Refused<br>Don't know	1156 (8.5)<br>2 (0.0)<br>6 (0.0)	2 (0.0)<br>4 (0.0)	0 (0.0)<br>2 (0.2)	
Tried to lose weight in past year (%)<br>No<br>Yes<br>Refused<br>Don't know	 7645 (56.5)<br>4224 (31.2)<br>1 (0.0)<br>7 (0.1)	 6980 (56.7)<br>3826 (31.1)<br>1 (0.0)<br>5 (0.0)	 665 (54.2)<br>398 (32.4)<br>0 (0.0)<br>2 (0.2)	0.168
Smoke (%)<br>Non-smoker<br>Smoker	 258 (1.9)<br>13274 (98.1)	 237 (1.9)<br>12067 (98.1)	 21 (1.7)<br>1207 (98.3)	0.675

Note: # - number, SP - Sample Person, pres - pressure, rdg - reading, HDL - high-density lipoprotein, HH - Household

Table C: Baseline characteristics of the study population stratified by clusters. The table compares demographic, clinical, dietary, examination, laboratory, and questionnaire-based variables between individuals with and without depressive symptoms, as defined by PHQ-9 criteria. Statistical significance for group differences was assessed using appropriate tests (e.g., t-test or chi-square), with corresponding p-values reported.

Characteristic	Total	Clusters 1	Clusters 2	Clusters 3	*p*-value
Sample Size	13532	1656	5970	5906	-
Total PHQ-9 Scores (mean (SD))	3.28 (4.38)	3.42 (4.75)	3.72 (4.60)	2.80 (3.98)	<0.001
Depressive symptoms (%)<br>No<br>Yes	 12304 (90.9)<br>1228 (9.1)	 1481 (89.4)<br>175 (10.6)	 5319 (89.1)<br>651 (10.9)	 5504 (93.2)<br>402 (6.8)	<0.001
1. Demographic					
Age in years at screening (mean (SD))	45.95 (18.18)	46.20 (15.43)	46.04 (18.46)	45.79 (18.60)	0.629
Total # of people in the Household (mean (SD))	3.28 (1.72)	4.49 (1.83)	3.10 (1.64)	3.12 (1.64)	<0.001
Total # of people in the Family (mean (SD))	3.06 (1.77)	4.13 (1.96)	2.93 (1.69)	2.88 (1.70)	<0.001
Ratio of family income to poverty (mean (SD))	2.44 (1.65)	1.40 (1.03)	2.50 (1.66)	2.65 (1.66)	<0.001
Sex (%)<br>Female<br>Male	 6784 (50.1)<br>6748 (49.9)	 787 (47.5)<br>869 (52.5)	 5945 (99.6)<br>25 (0.4)	 52 (0.9)<br>5854 (99.1)	<0.001
Race/Hispancic origin (%)<br>Mexican American<br>Non-Hispanic Black<br>Non-Hispanic White<br>Other Hispanic<br>Other Race - Including Multi-Racial	 2139 (15.8)<br>2981 (22.0)<br>5597 (41.4)<br>1351 (10.0)<br>1464 (10.8)	 987 (59.6)<br>8 (0.5)<br>10 (0.6)<br>582 (35.1)<br>69 (4.2)	 599 (10.0)<br>1471 (24.6)<br>2737 (45.8)<br>415 (7.0)<br>748 (12.5)	 553 (9.4)<br>1502 (25.4)<br>2850 (48.3)<br>354 (6.0)<br>647 (11.0)	<0.001
Citizenship status (%)<br>Citizen by birth or naturalization<br>Not a citizen of the US<br>Don't know<br>Refused	 11511 (85.1)<br>1990 (14.7)<br>4 (0.0)<br>22 (0.2)	 460 (27.8)<br>1175 (71.0)<br>2 (0.1)<br>16 (1.0)	 5563 (93.2)<br>403 (6.8)<br>1 (0.0)<br>3 (0.1)	 5488 (92.9)<br>412 (7.0)<br>1 (0.0)<br>3 (0.1)	<0.001
Language of SP Interview (%)<br>English<br>Spanish	 11862 (87.7)<br>1670 (12.3)	 141 (8.5)<br>1515 (91.5)	 5911 (99.0)<br>59 (1.0)	 5810 (98.4)<br>96 (1.6)	<0.001

Proxy used in SP Interview (%)					0.140
No	13480 (99.6)	1649 (99.6)	5954 (99.7)	5877 (99.5)	
Yes	52 (0.4)	7 (0.4)	16 (0.3)	29 (0.5)	
Interpreter used in SP Interview (%)					<0.001
No	13221 (97.7)	1495 (90.3)	5820 (97.5)	5906 (100.0)	
Yes	311 (2.3)	161 (9.7)	150 (2.5)	0 (0.0)	
Language of Family Interview (%)					<0.001
English	12304 (90.9)	674 (40.7)	5870 (98.3)	5760 (97.5)	
Spanish	1083 (8.0)	960 (58.0)	41 (0.7)	82 (1.4)	
Proxy used in Family Interview (%)					0.550
No	13381 (98.9)	1634 (98.7)	5909 (99.0)	5838 (98.8)	
Yes	6 (0.0)	0 (0.0)	2 (0.0)	4 (0.1)	
Interpreter used in Family Interview (%)					<0.001
No	13133 (97.1)	1516 (91.5)	5796 (97.1)	5821 (98.6)	
Yes	254 (1.9)	118 (7.1)	115 (1.9)	21 (0.4)	
Language of MEC Interview (%)					<0.001
No	12264 (90.6)	472 (28.5)	5926 (99.3)	5866 (99.3)	
Yes	1268 (9.4)	1184 (71.5)	44 (0.7)	40 (0.7)	
Interpreter used in MEC Interview (%)					<0.001
No	13188 (97.5)	1447 (87.4)	5843 (97.9)	5898 (99.9)	
Yes	344 (2.5)	209 (12.6)	127 (2.1)	8 (0.1)	
2. Dietary					
Total # of Dietary Supplements taken (mean (SD))	1.12 (2.18)	0.63 (2.19)	1.37 (1.94)	1.01 (2.36)	<0.001
Total # of Antacids Taken (mean (SD))	0.14 (1.56)	0.16 (3.09)	0.12 (0.34)	0.16 (1.67)	0.491
Dietary Supplements taken (%)					<0.001
No	7095 (52.4)	1147 (69.3)	2653 (44.4)	3295 (55.8)	
Yes	6432 (47.5)	508 (30.7)	3316 (55.5)	2608 (44.2)	
Refused	2 (0.0)	1 (0.1)	0 (0.0)	1 (0.0)	
Don’t know	1 (0.0)	0 (0.0)	0 (0.0)	1 (0.0)	
Antacids taken (%)					<0.001
No	12002 (88.7)	1577 (95.2)	5253 (88.0)	5172 (87.6)	
Yes	1525 (11.3)	77 (4.6)	717 (12.0)	731 (12.4)	
Refused	2 (0.0)	1 (0.1)	0 (0.0)	1 (0.0)	
Don’t know	2 (0.0)	1 (0.1)	0 (0.0)	1 (0.0)	
Number of days of intake (%)					<0.001
Day 1 only	1535 (11.3)	228 (13.8)	473 (7.9)	834 (14.1)	
Day 1 and day 2	11579 (85.6)	1348 (81.4)	5286 (88.5)	4945 (83.7)	
Intake day of the week (%)					<0.001
Monday	1058 (7.8)	77 (4.6)	488 (8.2)	493 (8.3)	
Tuesday	1243 (9.2)	104 (6.3)	576 (9.6)	563 (9.5)	
Wednesday	1222 (9.0)	147 (8.9)	523 (8.8)	552 (9.3)	
Thursday	1460 (10.8)	122 (7.4)	660 (11.1)	678 (11.5)	
Friday	3066 (22.7)	439 (26.5)	1334 (22.3)	1293 (21.9)	
Saturday	2870 (21.2)	470 (28.4)	1210 (20.3)	1190 (20.1)	
Sunday	2250 (16.6)	228 (13.8)	991 (16.6)	1031 (17.5)	
Language respondent used mostly - day one					<0.001
(%)	11385 (84.1)	133 (8.0)	5590 (93.6)	5662 (95.9)	
English	1517 (11.2)	1366 (82.5)	76 (1.3)	75 (1.3)	
Spanish	149 (1.1)	48 (2.9)	47 (0.8)	54 (0.9)	
English and Spanish	50 (0.4)	16 (1.0)	29 (0.5)	5 (0.1)	
Asian Languages	30 (0.2)	7 (0.4)	22 (0.4)	1 (0.0)	
Asian Languages and English	40 (0.3)	18 (1.1)	18 (0.3)	4 (0.1)	
Other					

Language respondent used mostly- two-day<br>(%)<br>English<br>Spanish<br>English and Spanish<br>Asian Languages<br>Asian Languages and English<br>Other	 10193 (75.3)<br>1302 (9.6)<br>86 (0.6)<br>62 (0.5)<br>2 (0.0)<br>5 (0.0)	 123 (7.4)<br>1187 (71.7)<br>31 (1.9)<br>12 (0.7)<br>0 (0.0)<br>4 (0.2)	 5170 (86.6)<br>68 (1.1)<br>22 (0.4)<br>41 (0.7)<br>2 (0.0)<br>1 (0.0)	 4900 (83.0)<br>47 (0.8)<br>33 (0.6)<br>9 (0.2)<br>0 (0.0)<br>0 (0.0)	<0.001
3. Examination					
Blood pressure: 60 sec. pulse (mean (SD))	72.73 (11.96)	71.68 (10.75)	74.67 (11.83)	71.07 (12.12)	<0.001
Blood pressure: maximum inflation levels (mm Hg) (mean (SD))	149.29 (23.26)	149.66 (17.82)	146.97 (25.86)	151.50 (21.56)	<0.001
Systolic: Blood pres (1st rdg) (mm Hg) (mean (SD))	122.89 (18.33)	123.53 (17.90)	120.80 (19.60)	124.77 (16.88)	<0.001
Diastolic: Blood pres (1st rdg) (mm Hg) (mean (SD))	70.11 (13.09)	69.72 (12.13)	68.70 (13.14)	71.61 (13.12)	<0.001
Systolic: Blood pres (2nd rdg) (mm Hg) (mean (SD))	122.22 (18.12)	122.26 (17.88)	120.23 (19.35)	124.20 (16.65)	<0.001
Diastolic: Blood pres (2nd rdg) (mm Hg) (mean (SD))	69.77 (13.12)	69.07 (12.60)	68.49 (13.15)	71.23 (13.08)	<0.001
Body Measures: Standing Height (cm) (mean (SD))	167.63 (10.13)	161.93 (9.20)	161.53 (6.91)	175.38 (7.50)	<0.001
Body Mass Index (kg/m^2) (mean (SD))	28.96 (7.00)	28.89 (5.46)	29.43 (7.92)	28.50 (6.33)	<0.001
Upper Leg Length (cm) (mean (SD))	38.79 (3.84)	36.55 (3.64)	37.09 (3.22)	41.13 (3.14)	<0.001
Upper Arm Length (cm) (mean (SD))	37.23 (2.82)	35.96 (2.45)	35.79 (2.26)	39.02 (2.37)	<0.001
Arm Circumference (cm) (mean (SD))	33.10 (5.14)	32.59 (3.97)	32.25 (5.64)	34.09 (4.72)	<0.001
Waist Circumference (cm) (mean (SD))	98.29 (16.63)	97.66 (13.23)	96.76 (17.27)	100.00 (16.67)	<0.001
Blood pressure: Pulse regular or irregular (%)<br>Regular<br>Irregular	 12943 (95.6)<br>325 (2.4)	 1595 (96.3)<br>14 (0.8)	 5703 (95.5)<br>127 (2.1)	 5645 (95.6)<br>184 (3.1)	<0.001
4. Laboratory					
Albumin, urine (ug/mL) (mean (SD))	36.72 (222.03)	46.36 (262.24)	27.95 (143.45)	42.86 (269.61)	<0.001
Creatinine, urine (umol/L) (mean (SD))	11077.58 (7247.52)	9862.39 (5741.66)	9733.07 (6898.10)	12780.78 (7611.24)	<0.001
Cadmium (nmol/L) (mean (SD))	4.48 (5.17)	3.40 (3.32)	4.85 (5.55)	4.41 (5.16)	<0.001
Lead (umol/L) (mean (SD))	0.07 (0.09)	0.09 (0.14)	0.06 (0.05)	0.08 (0.10)	<0.001
Mercury, total (nmol/L) (mean (SD))	7.69 (12.70)	7.93 (17.99)	7.47 (11.32)	7.85 (12.18)	0.205
Direct HDL-Cholesterol (mmol/L) (mean (SD))	1.36 (0.41)	1.26 (0.34)	1.49 (0.42)	1.26 (0.38)	<0.001
Total Cholesterol (mmol/L) (mean (SD))	4.96 (1.08)	5.15 (1.07)	5.03 (1.07)	4.85 (1.09)	<0.001
White blood cell count (1000 cells/uL) (mean (SD))	7.23 (4.16)	7.33 (1.91)	7.36 (5.68)	7.06 (2.48)	<0.001
Lymphocyte percent (%) (mean (SD))	30.67 (8.53)	31.22 (7.59)	31.03 (8.67)	30.16 (8.62)	<0.001

Monocyte percent (%) (mean (SD))	7.82 (2.42)	7.46 (1.99)	7.42 (2.25)	8.32 (2.60)	<0.001
Segmented neutrophils percent (%) (mean (SD))	57.99 (9.58)	57.84 (8.61)	58.26 (9.69)	57.76 (9.72)	0.017
Eosinophils percent (%) (mean (SD))	2.85 (2.12)	2.88 (2.42)	2.63 (1.90)	3.07 (2.21)	<0.001
Basophils percent (%) (mean (SD))	0.72 (0.55)	0.66 (0.46)	0.72 (0.51)	0.74 (0.59)	<0.001
Lymphocyte number (1000 cells/uL) (mean (SD))	2.17 (3.24)	2.24 (0.65)	2.26 (4.80)	2.07 (0.82)	0.005
Monocyte number (1000 cells/uL) (mean (SD))	0.55 (0.20)	0.54 (0.18)	0.52 (0.19)	0.57 (0.20)	<0.001
Segmented neutrophils num (1000 cell/uL) (mean (SD))	4.26 (1.87)	4.30 (1.50)	4.35 (1.85)	4.16 (1.98)	<0.001
Eosinophils number (1000 cells/uL) (mean (SD))	0.20 (0.19)	0.21 (0.20)	0.19 (0.15)	0.21 (0.21)	<0.001
Basophils number (1000 cells/uL) (mean (SD))	0.04 (0.07)	0.04 (0.06)	0.04 (0.06)	0.04 (0.09)	0.047
Red blood cell count (million cells/uL) (mean (SD))	4.65 (0.50)	4.70 (0.47)	4.41 (0.41)	4.88 (0.47)	<0.001
Hemoglobin (g/dL) (mean (SD))	14.07 (1.50)	14.16 (1.57)	13.23 (1.21)	14.89 (1.27)	<0.001
Mean cell volume (fL) (mean (SD))	89.26 (5.86)	88.79 (5.67)	88.80 (6.27)	89.85 (5.41)	<0.001
Mean cell hemoglobin (pg) (mean (SD))	30.33 (2.40)	30.15 (2.29)	30.11 (2.60)	30.61 (2.18)	<0.001
Mean cell hemoglobin concentration (g/dL) (mean (SD))	33.96 (1.05)	33.94 (0.95)	33.88 (1.09)	34.05 (1.02)	<0.001
Red cell distribution width (%) (mean (SD))	13.15 (1.35)	13.14 (1.47)	13.29 (1.48)	13.02 (1.16)	<0.001
Platelet count SI (1000 cells/uL) (mean (SD))	243.45 (63.30)	240.73 (58.17)	257.30 (65.51)	230.23 (59.38)	<0.001
Mean platelet volume (fL) (mean (SD))	8.21 (0.94)	8.31 (0.93)	8.24 (0.95)	8.15 (0.92)	<0.001
Cotinine (ng/mL) (mean (SD))	55.54 (124.55)	18.27 (61.04)	45.62 (109.29)	76.14 (146.80)	<0.001
Glycohemoglobin (%) (mean (SD))	5.71 (1.06)	5.95 (1.30)	5.64 (0.96)	5.70 (1.06)	<0.001
Mercury, inorganic (umol/L) (mean (SD))	1.43 (2.35)	1.71 (5.94)	1.44 (1.37)	1.34 (0.84)	<0.001
Albumin (g/dL) (mean (SD))	4.27 (0.35)	4.28 (0.32)	4.16 (0.34)	4.36 (0.33)	<0.001
Alanine aminotransferase ALT (U/L) (mean (SD))	25.62 (22.51)	29.03 (23.48)	21.23 (15.05)	29.04 (27.21)	<0.001
Aspartate aminotransferase AST (U/L) (mean (SD))	25.94 (17.89)	26.94 (24.27)	23.72 (12.91)	27.88 (19.69)	<0.001
Alkaline phosphatase (U/L) (mean (SD))	69.53 (23.81)	75.03 (22.07)	68.32 (24.24)	69.16 (23.65)	<0.001
Blood urea nitrogen (mmol/L) (mean (SD))	4.65 (1.99)	4.68 (1.74)	4.38 (1.96)	4.91 (2.04)	<0.001
Total calcium (mmol/L) (mean (SD))	2.36 (0.09)	2.34 (0.09)	2.35 (0.09)	2.37 (0.09)	<0.001
Cholesterol (mmol/L) (mean (SD))	4.96 (1.09)	5.16 (1.08)	5.02 (1.08)	4.83 (1.09)	<0.001
Bicarbonate (mmol/L) (mean (SD))	25.14 (2.24)	25.05 (2.11)	24.84 (2.31)	25.47 (2.16)	<0.001

Creatinine (umol/L) (mean (SD))	79.09 (37.61)	70.19 (31.08)	69.38 (26.29)	91.38 (44.72)	<0.001
Gamma glutamyl transferase (U/L) (mean (SD))	28.20 (39.61)	28.80 (29.58)	23.75 (35.76)	32.49 (44.95)	<0.001
Glucose, serum (mmol/L) (mean (SD))	5.61 (2.12)	6.01 (2.62)	5.45 (1.98)	5.65 (2.08)	<0.001
Iron, refrigerated (umol/L) (mean (SD))	15.24 (6.37)	15.40 (6.44)	13.93 (6.20)	16.52 (6.25)	<0.001
Lactate dehydrogenase LDH (U/L) (mean (SD))	130.59 (35.52)	129.64 (40.64)	130.09 (29.89)	131.37 (38.96)	0.086
Phosphorus (mmol/L) (mean (SD))	1.21 (0.19)	1.20 (0.18)	1.24 (0.18)	1.19 (0.19)	<0.001
Bilirubin, total (umol/L) (mean (SD))	11.88 (5.33)	11.53 (4.73)	10.53 (4.39)	13.34 (5.94)	<0.001
Total protein (g/dL) (mean (SD))	7.17 (0.48)	7.31 (0.46)	7.11 (0.47)	7.20 (0.47)	<0.001
Triglycerides (mmol/L) (mean (SD))	1.71 (1.54)	2.10 (2.30)	1.51 (1.16)	1.79 (1.57)	<0.001
Uric acid (umol/L) (mean (SD))	322.76 (84.59)	310.34 (80.91)	288.65 (76.36)	360.51 (77.61)	<0.001
Sodium (mmol/L) (mean (SD))	139.21 (2.27)	139.14 (1.99)	139.05 (2.34)	139.38 (2.26)	<0.001
Potassium (mmol/L) (mean (SD))	3.97 (0.34)	3.95 (0.34)	3.92 (0.34)	4.02 (0.34)	<0.001
Chloride (mmol/L) (mean (SD))	103.74 (2.89)	104.21 (2.74)	103.96 (2.93)	103.39 (2.86)	<0.001
Osmolality (mmol/Kg) (mean (SD))	278.11 (5.08)	278.44 (4.47)	277.39 (5.24)	278.72 (4.98)	<0.001
Globulin (g/L) (mean (SD))	29.07 (4.57)	30.24 (4.30)	29.44 (4.53)	28.35 (4.57)	<0.001
Mercury, total comment code (%)					0.007
At or above the detection limit	11675 (86.3)	1481 (91.6)	5144 (89.6)	5050 (88.9)	
Below lower detection limit	1368 (10.1)	136 (8.4)	600 (10.4)	632 (11.1)	
Fasting: Coffee or tea with cream or sugar? (%)					0.479
No	13254 (97.9)	1630 (99.0)	5833 (98.8)	5791 (99.0)	
Yes	147 (1.1)	16 (1.0)	72 (1.2)	59 (1.0)	
Fasting: Alcohol, such as beer, wine, or liquor? (%)					0.006
No	13349 (98.6)	1641 (99.7)	5892 (99.8)	5816 (99.4)	
Yes	52 (0.4)	5 (0.3)	13 (0.2)	34 (0.6)	
Fasting: Gum, breath mints, lozenges or cough drops, or other cough or cold remedies? (%)					0.147
No	13024 (96.2)	1595 (96.9)	5725 (97.0)	5704 (97.5)	
Yes	377 (2.8)	51 (3.1)	180 (3.0)	146 (2.5)	
Fasting: Antacids, laxatives, or anti-diarrheals? (%)					0.415
No	13346 (98.6)	1642 (99.8)	5877 (99.5)	5827 (99.6)	
Yes	55 (0.4)	4 (0.2)	28 (0.5)	23 (0.4)	
Fasting: Dietary supplements such as vitamins and minerals? (%)					0.114
No	13289 (99.2)	1637 (99.5)	5861 (99.3)	5791 (99.0)	
Yes	112 (0.8)	9 (0.5)	44 (0.7)	59 (1.0)	
Session in which SP was examined (%)					0.099
Afternoon	4890 (36.1)	570 (34.4)	2222 (37.2)	2098 (35.5)	
Evening	2148 (15.9)	255 (15.4)	927 (15.5)	966 (16.4)	
Morning	6494 (48.0)	831 (50.2)	2821 (47.3)	2842 (48.1)	

Hepatitis A Antibody (Anti-HAV) (%)					<0.001
Indeterminate	13 (0.1)	0 (0.0)	6 (0.1)	7 (0.1)	
Negative	6946 (51.3)	89 (5.6)	3417 (61.3)	3440 (62.4)	
Positive	5710 (42.2)	1496 (94.4)	2148 (38.6)	2066 (37.5)	
Hepatitis B Surface Antibody (%)					<0.001
Negative	9357 (69.1)	1370 (85.3)	3832 (67.8)	4155 (73.9)	
Positive	3527 (26.1)	236 (14.7)	1822 (32.2)	1469 (26.1)	
Hepatitis B core antibody (%)					0.671
Negative	12020 (88.8)	1492 (92.8)	5285 (93.5)	5243 (93.2)	
Positive	866 (6.4)	115 (7.2)	370 (6.5)	381 (6.8)	
Mercury, inorganic comment code (%)					<0.001
At or above the detection limit	2857 (21.1)	343 (21.2)	1385 (24.2)	1129 (19.9)	
Below lower detection limit	10165 (75.1)	1274 (78.8)	4347 (75.8)	4544 (80.1)	
5. Questionnaire					
Number of rooms in home (mean (SD))	10.08 (2.85)	9.66 (1.93)	10.19 (2.88)	10.08 (3.01)	<0.001
Current self-reported height (inches) (mean (SD))	201.37 (1148.87)	1067.16 (2993.65)	83.99 (445.39)	84.41 (378.94)	<0.001
Current self-reported weight (pounds) (mean (SD))	279.19 (983.05)	662.95 (2150.28)	237.59 (821.81)	214.14 (440.55)	<0.001
Self-reported weight - 1 yr ago (pounds) (mean (SD))	318.85 (1158.24)	724.29 (2271.70)	263.50 (959.46)	261.27 (804.13)	<0.001
Self-reported greatest weight (pounds) (mean (SD))	313.59 (1065.36)	640.91 (2076.86)	272.06 (918.03)	264.18 (713.93)	<0.001
Self-reported greatest weight (pounds) (mean (SD))	560.38 (7182.72)	739.68 (8334.99)	548.01 (7112.13)	524.76 (6915.49)	0.565
Ever told you had high blood pressure (%)					<0.001
No	9181 (67.8)	1216 (73.4)	4016 (67.3)	3949 (66.9)	
Yes	4331 (32.0)	431 (26.0)	1952 (32.7)	1948 (33.0)	
Don't know	20 (0.1)	9 (0.5)	2 (0.0)	9 (0.2)	
Current Health Status: General health condition (%)	1305 (9.7)	98 (5.9)	539 (9.1)	668 (11.4)	<0.001
Excellent	3645 (26.9)	146 (8.8)	1708 (28.6)	1791 (30.3)	
Very good	5441 (40.2)	664 (40.1)	2435 (40.8)	2342 (39.7)	
Good	2666 (19.7)	658 (39.7)	1068 (17.9)	940 (15.9)	
Fair	475 (3.5)	90 (5.4)	220 (3.7)	165 (2.8)	
Poor					
SP have head cold or chest cold (%)					<0.001
No	11266 (83.3)	1293 (78.1)	4964 (83.1)	5009 (84.8)	
Yes	2250 (16.6)	362 (21.9)	996 (16.7)	892 (15.1)	
Don't know	16 (0.1)	1 (0.1)	10 (0.2)	5 (0.1)	
SP have stomach or intestinal illness (%)					<0.001
No	12535 (92.6)	1550 (93.6)	5468 (91.6)	5517 (93.4)	
Yes	988 (7.3)	104 (6.3)	498 (8.3)	386 (6.5)	
Don't know	9 (0.1)	2 (0.1)	4 (0.1)	3 (0.1)	
SP have flu, pneumonia, ear infection (%)					0.014
No	12961 (95.8)	1578 (95.3)	5688 (95.3)	5695 (96.4)	
Yes	553 (4.1)	77 (4.6)	271 (4.5)	205 (3.5)	
Don't know	18 (0.1)	1 (0.1)	11 (0.2)	6 (0.1)	
SP donated blood in past 12 months (%)					<0.001
No	12870 (95.1)	1635 (98.7)	5689 (95.3)	5546 (93.9)	
Yes	654 (4.8)	21 (1.3)	280 (4.7)	353 (6.0)	
Don't know	8 (0.1)	0 (0.0)	1 (0.0)	7 (0.1)	
Blood ever tested for HIV virus (%)					<0.001
No	8059 (59.6)	1142 (69.0)	3446 (57.7)	3471 (58.8)	
Yes	5037 (37.2)	478 (28.9)	2334 (39.1)	2225 (37.7)	

Don’t know	436 (3.2)	36 (2.2)	190 (3.2)	210 (3.6)	
Doctor told you have diabetes (%)					<0.001
No	11710 (86.5)	1398 (84.4)	5200 (87.1)	5112 (86.6)	
Yes	1528 (11.3)	230 (13.9)	626 (10.5)	672 (11.4)	
Borderline	287 (2.1)	25 (1.5)	140 (2.3)	122 (2.1)	
Don’t know	7 (0.1)	3 (0.2)	4 (0.1)	0 (0.0)	
Taking insulin now (%)					0.009
No	13096 (96.8)	1610 (97.2)	5809 (97.3)	5677 (96.1)	
Yes	434 (3.2)	46 (2.8)	160 (2.7)	228 (3.9)	
Don’t know	1 (0.0)	0 (0.0)	1 (0.0)	0 (0.0)	
How healthy is the diet (%)					<0.001
Excellent	1144 (8.5)	110 (6.6)	448 (7.5)	586 (9.9)	
Very good	2619 (19.4)	122 (7.4)	1282 (21.5)	1215 (20.6)	
Good	5630 (41.6)	686 (41.4)	2488 (41.7)	2456 (41.6)	
Fair	3336 (24.7)	662 (40.0)	1357 (22.7)	1317 (22.3)	
Poor	795 (5.9)	74 (4.5)	392 (6.6)	329 (5.6)	
Don’t know	8 (0.1)	2 (0.1)	3 (0.1)	3 (0.1)	
Past 30 day milk product consumption (%)					0.014
Never	2325 (17.2)	304 (18.4)	1052 (17.6)	969 (16.4)	
Rarely-less than once a week	2297 (17.0)	292 (17.6)	1059 (17.7)	946 (16.0)	
Sometimes-less than once a day	3877 (28.7)	434 (26.3)	1717 (28.8)	1726 (29.2)	
Often-once a day or more	4998 (36.9)	619 (37.4)	2127 (35.6)	2252 (38.1)	
Varied	35 (0.3)	7 (0.4)	15 (0.3)	13 (0.2)	
HH Worried run out of food (%)					<0.001
Never true	9466 (70.0)	803 (48.5)	4287 (71.8)	4376 (74.1)	
Sometimes true	2555 (18.9)	513 (31.0)	1039 (17.4)	1003 (17.0)	
Often true	1366 (10.1)	310 (18.7)	588 (9.8)	468 (7.9)	
Don’t know	15 (0.1)	4 (0.2)	4 (0.1)	7 (0.1)	
Refused	20 (0.1)	9 (0.5)	6 (0.1)	5 (0.1)	
HH Food didn't last (%)					<0.001
Never true	10047 (74.2)	852 (51.4)	4578 (76.7)	4617 (78.2)	
Sometimes true	2309 (17.1)	505 (30.5)	927 (15.5)	877 (14.8)	
Often true	1040 (7.7)	273 (16.5)	412 (6.9)	355 (6.0)	
Don’t know	7 (0.1)	0 (0.0)	2 (0.0)	5 (0.1)	
Refused	19 (0.1)	9 (0.5)	5 (0.1)	5 (0.1)	
HH Couldn't afford balanced meals (%)					<0.001
Never true	10658	1051 (63.5)	4803 (80.5)	4804 (81.3)	
Sometimes true	(78.8)	390 (23.6)	760 (12.7)	708 (12.0)	
Often true	1858 (13.7)	187 (11.3)	355 (5.9)	337 (5.7)	
Don’t know	879 (6.5)	2 (0.1)	1 (0.0)	6 (0.1)	
Refused	9 (0.1)	9 (0.5)	5 (0.1)	4 (0.1)	
	18 (0.1)				
Household food security category (%)					<0.001
HH full food security: 0	8953 (66.2)	723 (43.7)	4059 (68.0)	4171 (70.6)	
HH marginal food security: 1-2	1606 (11.9)	283 (17.1)	710 (11.9)	613 (10.4)	
HH low food security: 3-5 / 3-7 (HH with child)	1730 (12.8)	436 (26.3)	682 (11.5)	612 (10.4)	
HH very low food security: 6-10 / 8-18 (HH with child)	1110 (8.2)	188 (11.3)	467 (7.9)	455 (7.7)	
Adult food security category (%)					<0.001
AD full food security: 0	8996 (66.5)	731 (44.1)	4080 (68.3)	4185 (70.9)	
AD marginal food security: 1-2	1680 (12.4)	304 (18.4)	736 (12.3)	640 (10.8)	
AD low food security: 3-5	1502 (11.1)	386 (23.3)	578 (9.7)	538 (9.1)	
AD very low food security: 6-10	1221 (9.0)	209 (12.6)	524 (8.8)	488 (8.3)	
HH Emergency food received (%)					0.006
No	11899	1415 (85.4)	5252 (88.0)	5232 (88.6)	
Yes	(87.9)	217 (13.1)	661 (11.1)	614 (10.4)	

Don't know<br>Refused	1492 (11.0)<br>15 (0.1)<br>16 (0.1)	1 (0.1)<br>6 (0.4)	6 (0.1)<br>5 (0.1)	8 (0.1)<br>5 (0.1)	
Covered by health insurance (%)<br>No<br>Yes<br>Don't know<br>Refused	 3294 (24.3)<br>10220 (75.5)<br>15 (0.1)<br>2 (0.0)	 934 (56.4)<br>721 (43.5)<br>1 (0.1)<br>0 (0.0)	 1027 (17.2)<br>4933 (82.6)<br>8 (0.1)<br>2 (0.0)	 1333 (22.6)<br>4566 (77.3)<br>6 (0.1)<br>0 (0.0)	<0.001
Hospital Utilization & Access to Care:<br>General health condition (%)<br>Excellent<br>Very good<br>Good<br>Fair<br>Poor<br>Don't know<br>Refused	 1801 (13.3)<br>3637 (26.9)<br>5035 (37.2)<br>2482 (18.3)<br>572 (4.2)<br>4 (0.0)<br>1 (0.0)	 104 (6.3)<br>150 (9.1)<br>652 (39.4)<br>639 (38.6)<br>109 (6.6)<br>2 (0.1)<br>0 (0.0)	 784 (13.1)<br>1730 (29.0)<br>2242 (37.6)<br>974 (16.3)<br>239 (4.0)<br>1 (0.0)<br>0 (0.0)	 913 (15.5)<br>1757 (29.7)<br>2141 (36.3)<br>869 (14.7)<br>224 (3.8)<br>1 (0.0)<br>1 (0.0)	<0.001
Health now compared with 1 year ago (%)<br>About the same<br>Better<br>Worse<br>Don't know	 9329 (68.9)<br>2678 (19.8)<br>1522 (11.2)<br>3 (0.0)	 1163 (70.2)<br>279 (16.8)<br>214 (12.9)<br>0 (0.0)	 4100 (68.7)<br>1165 (19.5)<br>703 (11.8)<br>2 (0.0)	 4066 (68.8)<br>1234 (20.9)<br>605 (10.2)<br>1 (0.0)	0.001
Routine place to go for healthcare (%)<br>Yes<br>No place<br>More than one place<br>Don't know	 11106 (82.1)<br>2348 (17.4)<br>76 (0.6)<br>2 (0.0)	 1075 (64.9)<br>573 (34.6)<br>8 (0.5)<br>0 (0.0)	 5288 (88.6)<br>641 (10.7)<br>40 (0.7)<br>1 (0.0)	 4743 (80.3)<br>1134 (19.2)<br>28 (0.5)<br>1 (0.0)	<0.001
Overnite hospital patient in last year (%)<br>No<br>Yes<br>Don't know	 11934<br>(88.2)<br>1593 (11.8)<br>5 (0.0)	 1466 (88.5)<br>190 (11.5)<br>0 (0.0)	 5134 (86.0)<br>833 (14.0)<br>3 (0.1)	 5334 (90.3)<br>570 (9.7)<br>2 (0.0)	<0.001
Seen mental health professional/past yr (%)<br>No<br>Yes<br>Don't know	 12416 (91.8)<br>1115 (8.2)<br>1 (0.0)	 1582 (95.5)<br>74 (4.5)<br>0 (0.0)	 5401 (90.5)<br>569 (9.5)<br>0 (0.0)	 5433 (92.0)<br>472 (8.0)<br>1 (0.0)	<0.001
Home owned, bought, rented, other (%)<br>Owned or being bought<br>Other arrangement<br>Rented<br>Don't know<br>Refused	 7836 (57.9)<br>397 (2.9)<br>5174 (38.2)<br>5 (0.0)<br>4 (0.0)	 685 (41.4)<br>35 (2.1)<br>914 (55.2)<br>1 (0.1)<br>3 (0.2)	 3545 (59.4)<br>180 (3.0)<br>2195 (36.8)<br>1 (0.1)<br>0 (0.0)	 3606 (61.1)<br>182 (3.1)<br>2065 (35.0)<br>3 (0.1)<br>1 (0.0)	<0.001
Received hepatitis A vaccine (%)<br>No doses<br>Less than 2 doses<br>Yes, at least 2 doses<br>Don't know<br>Refused	 7958 (58.8)<br>346 (2.6)<br>3331 (24.6)<br>1886 (13.9)	 913 (55.1)<br>37 (2.2)<br>336 (20.3)<br>370 (22.3)	 3472 (58.2)<br>168 (2.8)<br>1562 (26.2)<br>761 (12.7)	 3573 (60.5)<br>141 (2.4)<br>1433 (24.3)<br>755 (12.8)	<0.001
Received hepatitis B 3 dose series (%)<br>No doses<br>Less than 3 doses<br>Yes, at least 3 doses<br>Yes, all 3 doses<br>Don't know<br>Refused	 7237 (53.5)<br>342 (2.5)<br>2371 (17.5)<br>1878 (13.9)<br>1692 (12.5)<br>12 (0.1)	 935 (56.5)<br>38 (2.3)<br>141 (8.5)<br>160 (9.7)<br>381 (23.0)<br>1 (0.1)	 3004 (50.3)<br>173 (2.9)<br>1236 (20.7)<br>923 (15.5)<br>628 (10.5)<br>6 (0.1)	 3298 (55.8)<br>131 (2.2)<br>994 (16.8)<br>795 (13.5)<br>683 (11.6)<br>5 (0.1)	<0.001
Ever been told you have asthma (%)<br>No<br>Yes	 11481 (84.8)<br>2039 (15.1)	 1526 (92.1)<br>128 (7.7)	 4898 (82.0)<br>1070 (17.9)	 5057<br>(85.6)	<0.001

Don’t know	12 (0.1)	2 (0.1)	2 (0.0)	841 (14.2)<br>8 (0.1)	
Taking treatment for anemia/past 3 mos (%)<br>No<br>Yes<br>Don’t know	 12987 (96.0)<br>533 (3.9)<br>12 (0.1)	 1602 (96.7)<br>51 (3.1)<br>3 (0.2)	 5583 (93.5)<br>381 (6.4)<br>6 (0.1)	 5802 (98.2)<br>101 (1.7)<br>3 (0.1)	<0.001
Doctor ever said you were overweight (%)<br>No<br>Yes<br>Don’t know	 8926 (66.0)<br>4598 (34.0)<br>8 (0.1)	 1174 (70.9)<br>480 (29.0)<br>2 (0.1)	 3635 (60.9)<br>2334 (39.1)<br>1 (0.0)	 4117 (69.7)<br>1784 (30.2)<br>5 (0.1)	<0.001
Ever receive blood transfusion (%)<br>No<br>Yes<br>Don’t know<br>Refused	 11940<br>(88.2)<br>1446 (10.7)<br>144 (1.1)<br>2 (0.0)	 1505 (90.9)<br>144 (8.7)<br>7 (0.4)<br>0 (0.0)	 5125 (85.8)<br>782 (13.1)<br>61 (1.0)<br>2 (0.0)	 5310 (89.9)<br>520 (8.8)<br>76 (1.3)<br>0 (0.0)	<0.001
Type of work done last week (%)<br>Working at a job or business<br>With a job or business but not at work<br>Not working at a job or business<br>Looking for work<br>Refused<br>Don’t know	 7351 (54.3)<br>274 (2.0)<br><br>5285 (39.1)<br>615 (4.5)<br>1 (0.0)<br>5 (0.0)	 977 (59.0)<br>23 (1.4)<br><br>601 (36.3)<br>54 (3.3)<br>1 (0.1)<br>0 (0.0)	 3007 (50.4)<br>124 (2.1)<br><br>2596 (43.5)<br>237 (4.0)<br>0 (0.0)<br>5 (0.1)	 3367 (57.0)<br>127 (2.2)<br><br>2088 (35.4)<br>324 (5.5)<br>0 (0.0)<br>0 (0.0)	<0.001
Kind of work you have done the longest (%)<br>Same as current occupation<br>Enter occupation<br>Armed forces<br>Never worked<br>Refused<br>Don’t know	 5295 (39.1)<br>7540 (55.7)<br>95 (0.7)<br>581 (4.3)<br>14 (0.1)<br>6 (0.0)	 754 (45.5)<br>719 (43.4)<br>2 (0.1)<br>173 (10.4)<br>5 (0.3)<br>3 (0.2)	 2146 (35.9)<br>3553 (59.5)<br>7 (0.1)<br>256 (4.3)<br>4 (0.1)<br>3 (0.1)	 2395<br>(40.6)<br>3268 (55.3)<br>86 (1.5)<br>152 (2.6)<br>5 (0.1)<br>0 (0.0)	<0.001
Products used in home to control insects (%)<br>No<br>Yes<br>Don’t know	 12186 (90.1)<br>1310 (9.7)<br>36 (0.3)	 1486 (89.7)<br>165 (10.0)<br>5 (0.3)	 5372 (90.0)<br>585 (9.8)<br>13 (0.2)	 5328 (90.2)<br>560 (9.5)<br>18 (0.3)	0.837
Products used to kill weeds? (%)<br>No<br>Yes<br>Don’t know	 12694 (93.8)<br>681 (5.0)<br>157 (1.2)	 1605 (96.9)<br>44 (2.7)<br>7 (0.4)	 5579 (93.5)<br>287 (4.8)<br>104 (1.7)	 5510 (93.3)<br>350 (5.9)<br>46 (0.8)	<0.001
Taken prescription medicine, past month (%)<br>No<br>Yes<br>Refused	 6227 (46.0)<br>7302 (54.0)<br>2 (0.0)	 1013 (61.2)<br>642 (38.8)<br>1 (0.1)	 2271 (38.0)<br>3698 (61.9)<br>1 (0.0)	 2943 (49.8)<br>2962 (50.2)<br>0 (0.0)	<0.001
Ever told doctor had trouble sleeping (%)<br>No<br>Yes<br>Don’t know	 10226 (75.6)<br>3304 (24.4)<br>2 (0.0)	 1451 (87.6)<br>205 (12.4)<br>0 (0.0)	 4194 (70.3)<br>1775 (29.7)<br>1 (0.0)	 4581 (77.6)<br>1324 (22.4)<br>1 (0.0)	<0.001
Questionnaire Mode Flag (%)<br>A-CASI (12 - 19 Yrs)<br>Home Interview (18+ Yrs)<br>Home Interview (20+ Yrs)	 773 (5.7)<br>3762 (27.8)<br>8997 (66.5)	 76 (4.6)<br>409 (24.7)<br>1171 (70.7)	 337 (5.6)<br>1739 (29.1)<br>3894 (65.2)	 360 (6.1)<br>1614 (27.3)<br>3932 (66.6)	<0.001
How do you consider your weight (%)<br>About the right weight<br>Overweight<br>Underweight<br>Refused	 5723 (42.3)<br>7039 (52.0)<br>739 (5.5)<br>3 (0.0)	 776 (46.9)<br>791 (47.8)<br>73 (4.4)<br>0 (0.0)	 2082 (34.9)<br>3636 (60.9)<br>246 (4.1)<br>1 (0.0)	 2865 (48.5)<br>2612 (44.2)<br>420 (7.1)<br>2 (0.0)	<0.001

Don't know	27 (0.2)	16 (1.0)	5 (0.1)	6 (0.1)	
Like to weigh more, less or same (%)<br>Stay about the same<br>Less<br>More<br>Refused<br>Don't know	 4114 (30.4)<br>8253<br>(61.0)<br>1156 (8.5)<br>2 (0.0)<br>6 (0.0)	 643 (38.8)<br>919 (55.5)<br>92 (5.6)<br>0 (0.0)	 1393 (23.3)<br>4280 (71.7)<br>295 (4.9)<br>1 (0.0)	 2078 (35.2)<br>3054 (51.7)<br>769 (13.0)<br>1 (0.0)	<0.001
Tried to lose weight in past year (%)<br>No<br>Yes<br>Refused<br>Don't know	 7645 (56.5)<br>4224 (31.2)<br>1 (0.0)<br>7 (0.1)	 1056 (63.8)<br>427 (25.8)<br>0 (0.0)<br>4 (0.2)	 2878 (48.2)<br>2342 (39.2)<br>0 (0.0)<br>2 (0.0)	 3711 (62.8)<br>1455 (24.6)<br>1 (0.0)<br>1 (0.0)	<0.001
Smoke (%)<br>Non-smoker<br>Smoker	 258 (1.9)<br>13274 (98.1)	 28 (1.7)<br>1628 (98.3)	 95 (1.6)<br>5875 (98.4)	 135 (2.3)<br>5771 (97.7)	0.017

Note: # - number, SP - Sample Person, pres - pressure, rdg - reading, HDL - high-density lipoprotein, HH - Household

Table D: Mean AUC and 95% confidence intervals from 5-fold cross-validation. This table summarizes the predictive performance of four classification models, Logistic Regression, Random Forest, Gradient Boosting, and XGBoost evaluated within each of the three depression-related clusters. Performance is reported as the average area under the ROC curve (AUC) with 95% confidence intervals, based on stratified 5-fold cross-validation.

Model	Cluster 1 AUC (95% CI)	Cluster 2 AUC (95% CI)	Cluster 3 AUC (95% CI)
XGBoost	0.780 (95% CI: 0.766, 0.794)	0.834 (95% CI: 0.824, 0.844)	0.814 (95% CI: 0.804, 0.824)
Gradient Boosting	0.756 (95% CI: 0.746, 0.766)	0.824 (95% CI: 0.814, 0.834)	0.804 (95% CI: 0.794, 0.814)
Logistic Regression	0.744 (95% CI: 0.734, 0.754)	0.784 (95% CI: 0.774, 0.794)	0.774 (95% CI: 0.764, 0.784)
Random Forest	0.734 (95% CI: 0.724, 0.744)	0.814 (95% CI: 0.804, 0.824)	0.794 (95% CI: 0.784, 0.804)

Table E: Performance metrics including precision, recall, sensitivity, and specificity for each machine learning model across the three latent clusters derived from the SYNERGY-VAE framework. These metrics complement the ROC-AUC results and provide a more granular view of model performance within each subgroup, highlighting the variability in depression prediction across distinct health phenotypes.

ML Method	Precision	Recall	Sensitivity	Specificity
XGBoost - Cluster 1	0.78	0.75	0.75	0.8
XGBoost - Cluster 2	0.83	0.81	0.81	0.85
XGBoost - Cluster 3	0.81	0.78	0.78	0.83
Gradient Boosting - Cluster 1	0.76	0.73	0.73	0.78
Gradient Boosting - Cluster 2	0.82	0.8	0.8	0.84
Gradient Boosting - Cluster 3	0.8	0.77	0.77	0.82
Logistic Regression - Cluster 1	0.75	0.72	0.72	0.76

Logistic Regression - Cluster 2	0.78	0.76	0.76	0.8
Logistic Regression - Cluster 3	0.77	0.74	0.74	0.79
Random Forest - Cluster 1	0.74	0.71	0.71	0.75
Random Forest - Cluster 2	0.81	0.79	0.79	0.83
Random Forest - Cluster 3	0.79	0.76	0.76	0.81

Table F: Silhouette scores for PCA-based K-means clustering. This table presents the silhouette scores for cluster sizes ranging from 2 to 10, derived from applying K-means clustering to the PCA-transformed feature space. The relatively low silhouette values suggest limited cluster cohesion and separation using the PCA-based approach.

Number of Clusters (k)	Silhouette Score
2	0.15
3	0.112
4	0.142
5	0.138
6	0.147
7	0.148
8	0.158
9	0.151
10	0.139

Table G: Cluster-wise distribution of participants and depression prevalence using single decoder framework. This table summarizes the number of participants, depression cases, and corresponding prevalence for each of the ten clusters derived from the Single decoder + KMeans model. Depression prevalence remained low and relatively uniform across all clusters, ranging from 8.4% to 9.4%, with no significant association between cluster assignment and depression enrichment.

**Cluster ID**	**Participants**	**Depression Cases**	**Depression Prevalence (%)**
Cluster 1	1350	120	8.9
Cluster 2	1320	112	8.5
Cluster 3	1285	118	9.2
Cluster 4	1605	129	9.2
Cluster 5	1295	114	8.8
Cluster 6	1360	123	9.0
Cluster 7	1310	110	8.4
Cluster 8	1375	121	8.8
Cluster 9	1300	117	9.0
Cluster 10	1332	125	9.4

Table H: Sensitivity analysis of KL regularization coefficient (λ) on latent space quality and downstream clustering performance

λ Value	Reconstruction Loss (val)	KL Divergence (val)	ELBO (val)	Silhouette Score	Depression Enrichment Cluster 1 (p)	Depression Enrichment Cluster 2 (p)	Depression Enrichment Cluster 3 (p)	Cluster-Specific XGBoost AUC (mean

								across clusters)
0.5	0.142	3.21	−0.174	0.21	0.031	0.018	0.074	0.801
**1.0**	**0.158**	**2.87**	**−0.187**	**0.51**	**0.008**	**0.003**	**0.041**	**0.819**
2.0	0.173	2.44	−0.202	0.24	0.042	0.027	0.089	0.806
4.0	0.201	1.98	−0.221	0.17	0.118	0.051	0.203	

Table I(a): Cluster stability assessment across K-means initializations and bootstrap resamples

Stability Assessment	Configuration	N Runs	Mean ARI	SD	% Runs with ARI > 0.80
**K-means initialization stability**	Fixed encoder, varying K-means seed	50	0.94	0.03	100%
**Bootstrap resampling stability**	Full VAE retrain on bootstrap resample	100	0.89	0.05	91%

Table I(b): Cluster size consistency across bootstrap resamples

Cluster	Reference Solution (n)	Reference Solution (%)	Mean Bootstrap n	SD	Mean Bootstrap %	SD %
Cluster 1	1,656	12.2%	1,643	48	12.1%	0.4%
Cluster 2	5,970	44.1%	5,961	87	44.1%	0.6%
Cluster 3	5,906	43.6%	5,928	91	43.8%	0.7%

Table I(c): Consistency of cluster-specific depression prevalence across bootstrap resamples

Cluster	Reference Depression Prevalence	Mean Bootstrap Prevalence	SD	% Resamples Significant (p < 0.017, Bonferroni)
Cluster 1	10.6%	10.5%	0.6%	97%
Cluster 2	10.9%	10.8%	0.5%	98%
Cluster 3	6.8%	6.9%	0.5%	96%

Table J(a): Numerical silhouette scores and K-means stability metrics across k = 2 to 6

k	Silhouette Score	WSS (Within-Cluster Sum of Squares)	Mean ARI Across 50 Seeds (± SD)	% Runs ARI > 0.80
2	0.49	590,000	0.97 ± 0.02	100%
3	0.51	430,000	0.94 ± 0.03	100%
4	0.41	370,000	0.88 ± 0.05	89%
5	0.35	320,000	0.81 ± 0.07	74%
6	0.34	280,000	0.74 ± 0.09	61%

Table J(b). Sensitivity of depression enrichment and classification utility across candidate cluster numbers ($k = 2$ to 6)

k	Depression prevalence range across clusters	Mean cluster-specific XGBoost AUC	Mean pooled XGBoost AUC	AUC uplift (clustered – pooled)
2	8.0%–10.2%	0.801	0.682	+0.119
**3**	**6.8%–10.9%**	**0.819**	**0.682**	**+0.137**
4	7.1%–10.4%	0.804	0.682	+0.122
5	7.4%–10.1%	0.806	0.682	+0.124
6	7.6%–9.8%	0.803	0.682	+0.121

Table K: Sensitivity of XGBoost classification performance to PFI feature selection threshold across clusters

Number of Features	Cluster 1 AUC (95% CI)	Cluster 2 AUC (95% CI)	Cluster 3 AUC (95% CI)
**Top 20**	0.770 (0.703–0.839)	0.825 (0.793–0.869)	0.810 (0.766–0.856)
**Top 25**	0.784 ((0.712–0.850)	0.833 (0.798–0.872)	0.818 (0.771–0.859)
**Top 30***(selected)*	**0.794 (0.720–0.868)**	**0.839 (0.804–0.874)**	**0.823 (0.776–0.869)**
**Top 35**	0.786 (0.710–0.846)	0.834 (0.796–0.870)	0.818 (0.770–0.858)
**Top 40**	0.780 (0.704–0.842)	0.830 (0.791–0.867)	0.814 (0.765–0.854)

Table L(a): Baseline comparator specification, preprocessing and tuning details

Specification	SYNERGY-VAE + K-means (Proposed)	PCA + K-means (Baseline 1)	Single-Decoder VAE + K-means (Baseline 2)	Pooled Classifier (Baseline 3)
**Dimensionality reduction**	VAE encoder (nonlinear, 153 to 3)	PCA (linear, components)	Single-decoder VAE (nonlinear, 153 to 3)	None (full feature set)
**Number of latent dimensions**	3	3 (matched)	3 (matched)	N/A
**Decoder architecture**	5 modality-specific decoders	N/A	1 shared decoder (all modalities)	N/A
**Reconstruction loss**	Modality-specific MSE (5 terms)	N/A	Single MSE (all modalities combined)	N/A
**KL regularization (λ)**	1.0	N/A	1.0 (matched)	N/A
**Optimizer**	Adam (lr = 0.0001)	N/A	Adam (lr = 0.0001, matched)	N/A
**Training epochs**	400 (early stopping, patience=10)	N/A	400 (early stopping, patience=10, matched)	N/A
**Clustering algorithm**	K-means (k=3, k-means++ init, n_init=50)	K-means (k=3, k-means++ init, n_init=50, matched)	K-means (k=3, k-means++ init, n_init=50, matched)	N/A (no clustering)
**Cluster assignment**	Full-cohort VAE embeddings; K-means labels assigned prior to within-cluster train-test splitting	Full-cohort PCA embeddings; K-means labels assigned prior to within-cluster train-test splitting	Full-cohort VAE embeddings; K-means labels assigned prior to within-cluster train-test splitting	N/A
**Feature scaling**	StandardScaler (fit on train only)	StandardScaler (fit on train only, matched)	StandardScaler (fit on train only, matched)	StandardScaler (fit on train only, matched)
**Feature selection**	PFI top 30 (within training CV folds)	PFI top 30 (within training CV folds, matched)	PFI top 30 (within training CV folds, matched)	PFI top 30 (training data only; matched feature-selection framework)

**Class imbalance handling**	SMOTE (within training folds only)	SMOTE (within training folds only, matched)	SMOTE (within training folds only, matched)	SMOTE (within training folds only, matched)
**Classifier**	XGBoost (+ LR, RF, GB)	XGBoost (+ LR, RF, GB, matched)	XGBoost (+ LR, RF, GB, matched)	XGBoost (+ LR, RF, GB, matched)
**Hyperparameter tuning**	5-fold CV grid search (training set only)	5-fold CV grid search (training set only, matched)	5-fold CV grid search (training set only, matched)	5-fold CV grid search (training set only, matched)
**Hyperparameter grid**	n_estimators {100,200,300}, max_depth {3,5,7}, lr {0.01,0.05,0.1}, subsample {0.7,0.9}	Identical grid (matched)	Identical grid (matched)	Identical grid (matched)
**Final evaluation**	Bootstrap AUC (1,000 resamples, held-out test set)	Bootstrap AUC (1,000 resamples, matched)	Bootstrap AUC (1,000 resamples, matched)	Bootstrap AUC (1,000 resamples, matched)
**Silhouette score**	0.51	0.14	0.18	N/A
**Depression enrichment significant**	Yes (all 3 clusters)	No ($p > 0.05$ all clusters)	No ($p > 0.05$ all clusters)	N/A

Table L(b): Classification performance across all comparators, XGBoost AUC (95% CI)

Model	Cluster 1 AUC (95% CI)	Cluster 2 AUC (95% CI)	Cluster 3 AUC (95% CI)	Overall / Pooled AUC (95% CI)
**SYNERGY-VAE + K-means (Proposed)**	**0.794 (0.720–0.868)**	**0.839 (0.804–0.874)**	**0.823 (0.776–0.869)**	—
PCA + K-means (Baseline 1)	0.774 (0.697–0.851)	0.791 (0.753–0.829)	0.786 (0.737–0.835)	—
Single-Decoder VAE + K-means (Baseline 2)	0.779 (0.703–0.855)	0.801 (0.764–0.838)	0.798 (0.750–0.846)	—
Pooled Classifier — No Clustering (Baseline 3)	—	—	—	0.682 (0.672–0.692)

Table L(c): Statistical comparison of performance differences, Bootstrap ΔAUC (95% CI) for SYNERGY-VAE vs each comparator

Comparison	Cluster / Level	ΔAUC (SYNERGY-VAE minus Comparator)	Bootstrap 95% CI	Statistically Significant (CI excludes 0)
**SYNERGY-VAE vs PCA+K-means**	Cluster 1	+0.020	(+0.004, +0.038)	Yes
	Cluster 2	+0.048	(+0.029, +0.067)	Yes
	Cluster 3	+0.037	(+0.019, +0.055)	Yes
**SYNERGY-VAE vs Single-Decoder VAE**	Cluster 1	+0.015	(+0.001, +0.031)	Yes
	Cluster 2	+0.038	(+0.021, +0.055)	Yes
	Cluster 3	+0.025	(+0.009, +0.041)	Yes
**SYNERGY-VAE vs Pooled Classifier**	Cluster 1	+0.023	(+0.006, +0.042)	Yes
	Cluster 2	+0.068	(+0.047, +0.089)	Yes

	Cluster 3	+0.052	(+0.033, +0.071)	Yes

Table M(a): Concordance between SHAP, PFI, and encoder weight rankings — Spearman rank correlation (top 30 features per cluster)

Comparison	Cluster 1 ρ (95% CI)	Cluster 2 ρ (95% CI)	Cluster 3 ρ (95% CI)	Mean ρ Across Clusters	Interpretation
**SHAP vs PFI**	0.91 (0.84–0.96)	0.93 (0.87–0.97)	0.89 (0.81–0.95)	0.91	Very high concordance
**SHAP vs Encoder Weights**	0.61 (0.43–0.75)	0.58 (0.39–0.73)	0.64 (0.47–0.77)	0.61	Moderate concordance
**PFI vs Encoder Weights**	0.63 (0.45–0.77)	0.57 (0.38–0.72)	0.62 (0.44–0.76)	0.61	Moderate concordance

Table M(b) Table: Feature-level concordance summary: agreement in top 10 rankings across SHAP, PFI, and encoder weights per cluster

Cluster	Features in Top 10 for Both SHAP and PFI	Features in Top 10 for Both SHAP and Encoder Weights	Features Consistent Across All Three Methods	Key Discordant Features (Encoder Weights vs SHAP/PFI)
**Cluster 1**	8/10 (80%)	5/10 (50%)	5/10 (50%)	Physical activity level (high encoder rank, low SHAP/PFI); Hemoglobin (high encoder rank, low SHAP/PFI)
**Cluster 2**	8/10 (80%)	6/10 (60%)	5/10 (50%)	Mean cell hemoglobin (high encoder rank, moderate SHAP/PFI); Albumin (high encoder rank, lower SHAP)
**Cluster 3**	9/10 (90%)	6/10 (60%)	6/10 (60%)	Creatinine (elevated encoder rank vs SHAP); Dietary recall frequency (lower encoder rank vs SHAP/PFI)

Table N: Comparison of clustering performance across latent dimensionalities (2–10) in the SYNERGY-VAE framework. Latent dimension 3 achieved the highest silhouette score and a substantial reduction in within-cluster sum of squares (WSS), indicating optimal balance between cluster cohesion and separation. This justified the selection of 3 as the preferred latent dimensionality for subgroup discovery.

Latent Dimension	Silhouette Score	Within-Cluster Sum of Squares (WSS)
2	0.43	1180000
3	0.52	600000
4	0.5	420000
5	0.42	370000
6	0.35	320000
7	0.33	280000
8	0.35	230000
9	0.34	200000
10	0.31	180000

Table O: Predictive performance (AUC and 95% CI) of pooled models trained on the entire cohort (N = 13,532) without clustering. All models demonstrate substantially lower AUCs compared to their cluster-specific counterparts, highlighting the benefit of stratified modeling via SYNERGY-VAE.

Model	Pooled AUC (95% CI)
**XGBoost**	0.682 (95% CI: 0.672–0.692)
**Gradient Boosting**	0.664 (95% CI: 0.654–0.674)
**Logistic Regression**	0.648 (95% CI: 0.638–0.658)
**Random Forest**	0.632 (95% CI: 0.622–0.642)

Table P. Cycle-restricted robustness of clustering results

Analysis subset	n	Number of clusters retained	Cluster-specific depression prevalence (%)	Pattern broadly consistent with main analysis?
Main pooled cohort (2005–2018)	13,532	3	C1: 10.6, C2: 10.9, C3: 6.8	
Early cycles (2005–2010)	5,859	3	C1: 10.1, C2: 11.2, C3: 7.3	Yes
Middle cycles (2011–2014)	5,712	3	C1: 10.9, C2: 11.5, C3: 5.3	Yes
Late cycles (2015–2018)	1,961	3	C1: 11.1, C2: 10.2, C3: 6.5	Yes

Table Q: Benjamini-Hochberg adjusted q-values for top SMD-ranked cluster signature features

Cluster	Rank	NHANES modality	Feature	Benjamini-Hochberg q-value
Cluster 1	1	Demographic	Household size	0.041
Cluster 1	2	Demographic	Family poverty income ratio	0.019
Cluster 1	3	Dietary	Dietary Day 1 weight	0.003
Cluster 1	4	Dietary	Dietary 2-day weight	0.023
Cluster 1	5	Examination	Standing height	0.014
Cluster 1	6	Laboratory	Cotinine	0.005
Cluster 1	7	Laboratory	Total protein	0.011
Cluster 1	8	Laboratory	Direct HDL cholesterol	0.030
Cluster 1	9	Laboratory	Globulin	0.016
Cluster 1	10	Questionnaire	Self-reported height	0.016
Cluster 2	1	Examination	Standing height	0.012
Cluster 2	2	Laboratory	Hemoglobin	0.028
Cluster 2	3	Laboratory	Red blood cell count	0.024
Cluster 2	4	Laboratory	Uric acid	0.005
Cluster 2	5	Laboratory	Direct HDL cholesterol	0.020
Cluster 2	6	Laboratory	Albumin	0.027
Cluster 2	7	Laboratory	Creatinine	0.020
Cluster 2	8	Laboratory	Bilirubin	0.017
Cluster 2	9	Laboratory	Platelet count SI	0.002
Cluster 2	10	Laboratory	Refrigerated iron	0.016
Cluster 3	1	Examination	Blood pressure	0.012
Cluster 3	2	Laboratory	Cotinine	0.032
Cluster 3	3	Laboratory	Potassium	0.020
Cluster 3	4	Laboratory	Globulin	0.044
Cluster 3	5	Laboratory	Alanine aminotransferase	0.026
Cluster 3	6	Laboratory	Bicarbonate	0.017
Cluster 3	7	Laboratory	Blood urea nitrogen	0.011
Cluster 3	8	Laboratory	Chloride	0.021
Cluster 3	9	Laboratory	Total calcium	0.036
Cluster 3	10	Laboratory	Monocyte percent	0.030

Table R. Bootstrap comparison of cluster-specific XGBoost models versus pooled XGBoost model

Cluster	Cluster-specific model AUC	Pooled model AUC evaluated in same cluster test subset	ΔAUC	Bootstrap 95% CI for ΔAUC	Statistically significant?
Cluster 1	0.794 (0.720 - 0.868)	0.705 (0.683 - 0.719)	+0.089	(0.021, 0.154)	Yes
Cluster 2	0.839 (0.804 - 0.874)	0.774 (0.735 - 0.798)	+0.065	(0.024, 0.108)	Yes
Cluster 3	0.823 (0.776 - 0.869)	0.761 (0.724 - 0.787)	+0.062	(0.013, 0.111)	Yes

AUC, area under the receiver operating characteristic curve. ΔAUC was calculated as $AUC_{cluster\text{-}specific} - AUC_{pooled}$. The pooled XGBoost model was trained once on the full unclustered training cohort using the same preprocessing, SMOTE-based class rebalancing, and PFI-based feature-selection workflow as the cluster-specific models. For cluster-level comparison, pooled-model predictions were evaluated within the held-out test participants assigned to each latent cluster. Statistical significance was assessed using 1,000 bootstrap resamples of the paired held-out predictions within each cluster; ΔAUC confidence intervals excluding zero were considered statistically significant.

Fig A: Flow diagram of analytic cohort derivation across NHANES 2005–2018 cycles. The figure shows participant inclusion and exclusion at each survey cycle, beginning with the total NHANES sample and sequentially applying non-missing PHQ-9 availability and completeness across the five modeled domains (demographic, dietary, examination, laboratory, and questionnaire).

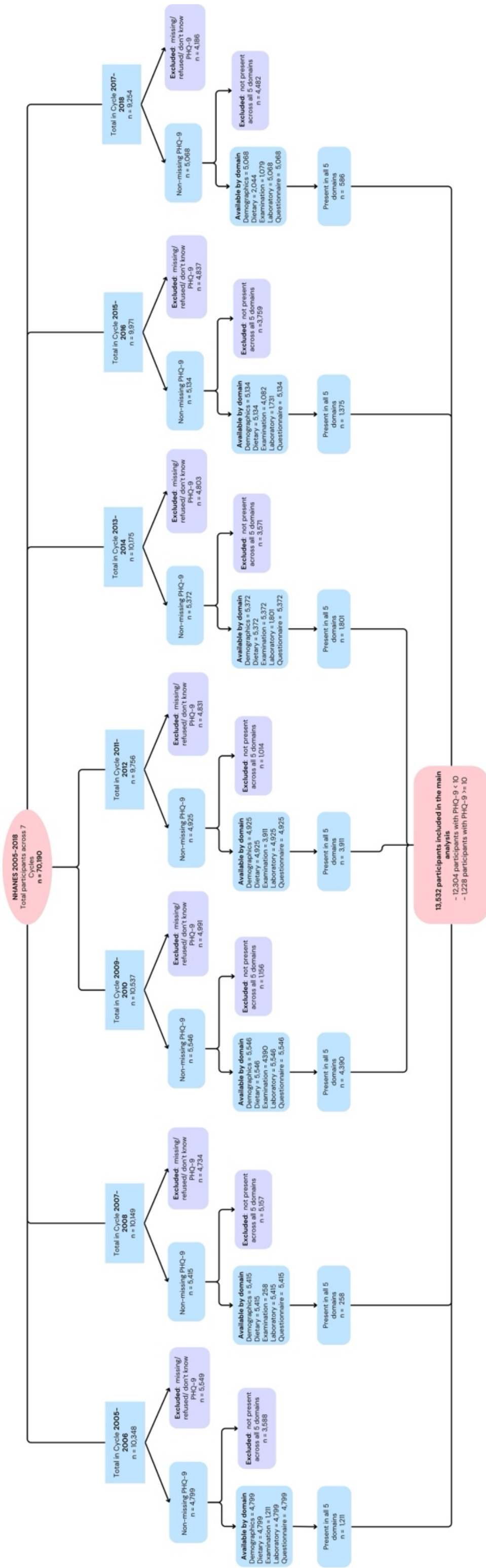

Evaluation of Single Encoder - Single Decoder Framework

The single-decoder VAE yielded a substantially higher reconstruction loss (binary cross-entropy: 0.45) compared to the multi-decoder setup (0.28), highlighting the inability of a shared decoder to accurately reconstruct heterogeneous multimodal inputs such as demographic, laboratory, dietary, and clinical questionnaire data. Visual inspection of the latent space revealed poor separation of clusters when using a single decoder. The learned representations did not preserve modality-specific structure, resulting in clusters that were neither interpretable nor associated with meaningful depression outcomes.

Fig B: Clustering performance metrics for single-decoder VAE latent space. (a) Silhouette scores across varying cluster sizes (k = 2 to 15) indicate poor overall cohesion and separation, with the highest score (~0.19) at k = 11. (b) Elbow method plot based on K-means inertia suggests a diminishing return in clustering compactness beyond k = 8-10. These results highlight the suboptimal structure captured by the single-decoder model compared to the multi-decoder SYNERGY-VAE.

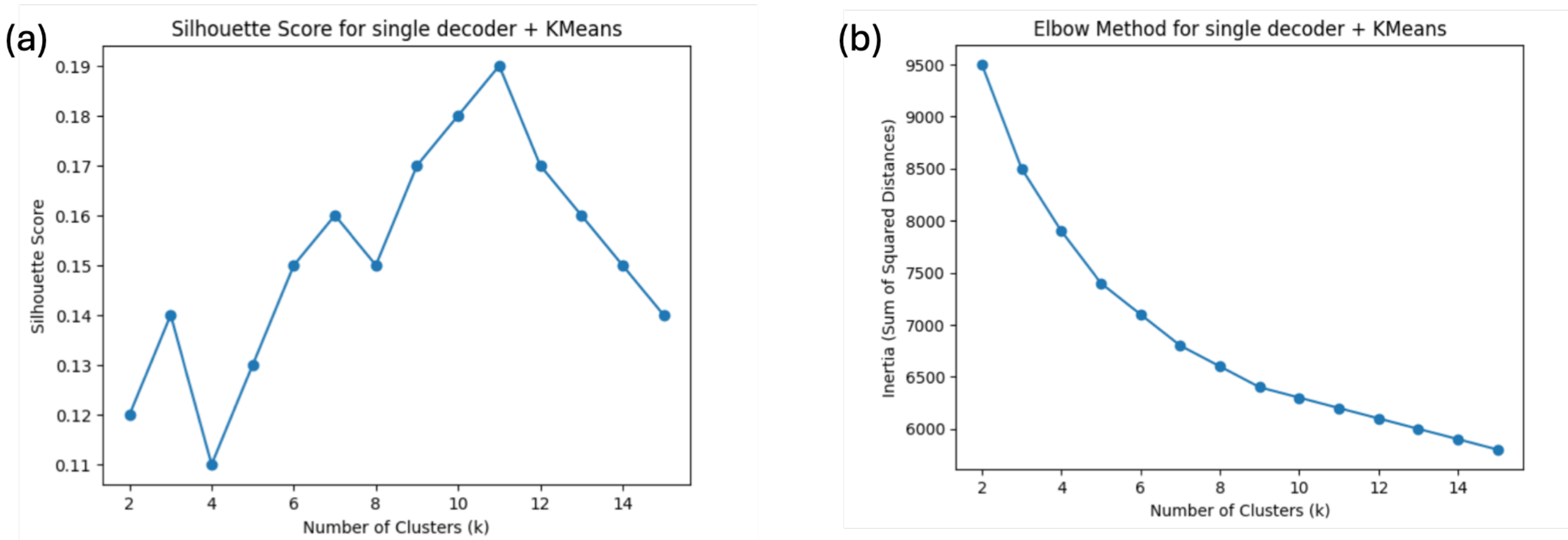

Fig C: Global SHAP feature importance: mean absolute SHAP value for top 10 features per cluster (XGBoost). Displays cluster-specific SHAP importance profiles for Clusters 1, 2, and 3, illustrating both shared and cluster-specific predictive features and supporting the concordance analysis with PFI and encoder weight rankings reported in Table M in S1 Appendix.

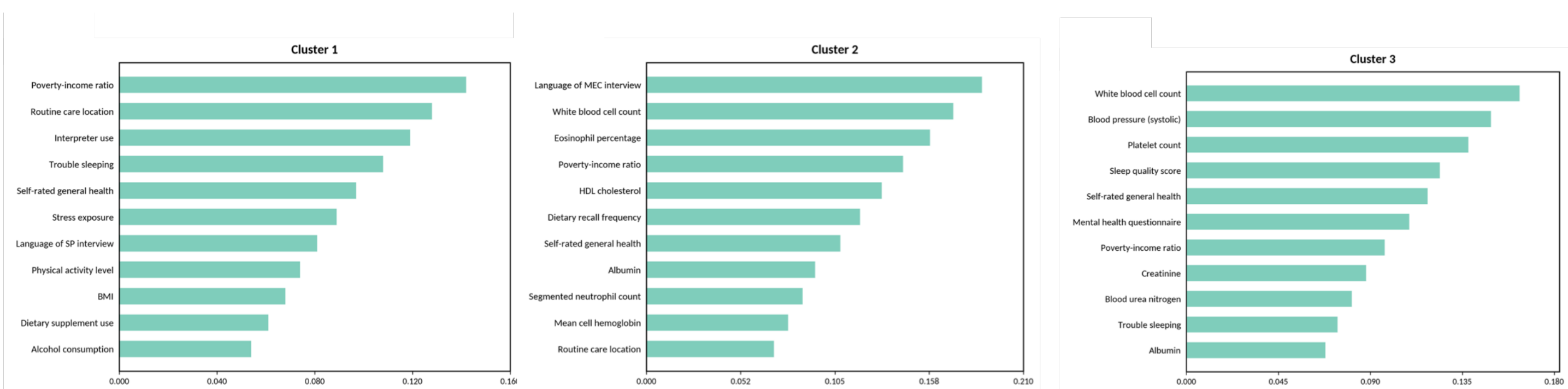

Supplement: Supplementary file 1 [file pdig.0001719.s001.pdf]
