# Supplementary material for: SYNERGY-VAE: An explainable generative deep learning framework for discovering depression subgroups from multimodal population health data
Source: PLOS Digit Health. 2026 Sep 24;5(9):e0001719. doi: 10.1371/journal.pdig.0001719 (PMC13606994; doi:10.1371/journal.pdig.0001719)
Supplement: Supplementary file 2 [file pdig.0001719.s003.pdf]

To:
Editor in Chief, PLOS Digital Health

Dear Editor,

We thank the editors and reviewers for their constructive comments regarding our manuscript. We have addressed all comments point-by-point below (with reviewer's comment in bold). For ease of navigation, the table of contents below is hyperlinked to each reviewer response. All page and paragraph references correspond to the attached tracked version of the revised manuscript where figures are inline.

Table of Contents:

Reviewer #1: 2

Reviewer #2: 6

Reviewer #3: 8

Reviewer #4: 17

Reviewer #1:

This study presents SYNERGY-VAE, a multimodal variational autoencoder integrating demographic, dietary, laboratory, examination, and questionnaire data from NHANES to identify latent subgroups associated with depression risk. The authors demonstrate that their framework uncovers clinically meaningful clusters with distinct biopsychosocial profiles and varying depression prevalence. Feature interpretability is enhanced through encoder weights, standardized mean differences, and permutation feature importance, highlighting relevant socioeconomic, physiological, and behavioral factors.
This is a well-conducted and innovative study. The manuscript demonstrates thoughtful methodology, interpretable analyses, and clear clinical relevance. However, a few methodological clarifications can strengthen the findings:

1.Clustering Methodology: Please clarify the rationale for using K-means rather than alternative clustering methods such as Gaussian Mixture Models, hierarchical clustering, or density-based approaches (e.g., HDBSCAN), which may capture different structures in the latent space.

We thank the reviewer for the comment. The rationale of using K means was because VAE latent spaces are explicitly regularized via the KL divergence term to approximate a standard isotropic Gaussian prior, encouraging compact, roughly spherical, and well-separated cluster geometries. This regularization makes K-means which assumes convex, isotropic clusters a natural and principled choice for partitioning VAE-derived embeddings [Kingma & Welling, 2014; Dilokthanakul et al., 2017]. Second, K-means offers computational scalability and interpretable centroid-based representations that facilitate downstream cluster profiling. Methods such as GMM, hierarchical clustering, or HDBSCAN, while powerful for general-purpose clustering, consider soft-assignment mechanism, which while useful in heterogeneous spaces, adds complexity without substantive benefit when the latent space is already well-regularized.

We thank the reviewer for this comment. We have added two supporting references to the K-means clustering sentence on Page 8, Section 2.2, Para 5, of the revised manuscript, which now reads: *"Once the SYNERGY-VAE is trained, K-means clustering[19,26] on the learned latent space is used to identify distinct subpopulations within the multimodal NHANES data "*

[19] Kingma, D.P., & Welling, M. (2014). Auto-encoding variational Bayes. *Proceedings of the 2nd International Conference on Learning Representations (ICLR 2014)*. arXiv:1312.6114. Available from: https://arxiv.org/abs/1312.6114

[26] Dilokthanakul, N., Mediano, P.A.M., Garnelo, M., Lee, M.C.H., Salimbeni, H., Arulkumaran, K., & Shanahan, M. (2017). Deep unsupervised clustering with Gaussian mixture variational autoencoders. *Proceedings of the 5th International Conference on Learning Representations (ICLR 2017)*. arXiv:1611.02648.

2. PHQ-9 Inclusion in VAE: It is unclear whether depression-related questionnaire items (e.g., PHQ-9 components) were included as inputs to the VAE. This clarification is essential to support claims that the subgroup discovery is unbiased with respect to depression status.

We thank the reviewer for raising this point. To clarify: PHQ-9 items and the derived binary depression label were not used as inputs to the SYNERGY-VAE**.** The VAE was trained exclusively on the five NHANES data domains: demographic (19 features), dietary (10 features), examination (13 features), laboratory (63 features), and questionnaire (48 features), where the questionnaire domain includes general health and lifestyle items, but explicitly excludes the PHQ-9 depression items. PHQ-9 scores were used only as an external outcome label for post-hoc evaluation of cluster enrichment and for training the cluster-specific supervised classifiers (Section 2.4). This architecture ensures that the latent subgroup discovery is fully unsupervised and unbiased with respect to depression status.

We have added a clarifying sentence in Page 7, Section 2.1, Paragraph 2: "*PHQ-9 items and the derived binary depression label were only used as outcome variables*".

3.Statistical Correction: Fisher's exact tests were used to assess enrichment of depression across clusters, but it is not stated whether multiple testing corrections were applied. The authors should clarify this to ensure appropriate statistical interpretation.

We thank the reviewer for highlighting this important methodological detail. To clarify: Fisher's exact tests were applied comparing depression prevalence in each cluster against the remaining population (three comparisons). Given the small number of pre-specified tests, Bonferroni correction was applied. Clusters 1 and 2 remained significantly enriched for depression after correction. We note that our SMD analysis (Section 2.5.2) already employed Benjamini-Hochberg correction for multiple comparisons across features ($q < 0.05$).

Specifically, we have added the word 'Bonferroni correction' to Page 8, Section 2.3, Para 1, Line 4: " *For each cluster, we calculated the proportion of individuals who met the threshold for depression and employed Fisher's exact test with Bonferroni correction to ascertain whether any clusters demonstrated significant enrichment for depression in comparison to the broader population.*"

4.KL Regularization (λ): The manuscript does not specify how the KL regularization coefficient (λ) was selected or tuned. Further explanation will help, as λ affects latent space regularization, cluster separability, and downstream interpretability.

To ensure that λ was an empirically supported choice, we conducted a systematic sensitivity analysis across $\lambda \in \{0.5, 1.0, 2.0, 4.0\}$, evaluating each configuration on three criteria directly relevant to our downstream objectives: validation ELBO convergence, latent space quality (silhouette score), and depression enrichment across clusters. Results are summarized in Supplementary Table 8.

Across all values tested, $\lambda = 1.0$ yielded the best balance of reconstruction fidelity and latent space regularity, producing the highest silhouette score and the most significant and consistent depression enrichment across all three clusters. Lower values ($\lambda = 0.5$) produced marginally better reconstruction loss but a less structured latent space (silhouette = 0.21), with weaker depression signal in Cluster 3. Higher values ($\lambda = 2.0$ and $\lambda = 4.0$) progressively degraded cluster separability, consistent with known over-smoothing effects of aggressive KL regularization in heterogeneous

tabular data. λ = 1.0 was therefore selected as the final configuration, corresponding to the standard VAE formulation (Kingma & Welling, 2014). We have added the following to Page 10, Section 3.2, Para 1, Line 4: *"KL Regularization coefficient (λ)=1.0 provided the best overall balance of latent space structure and downstream clustering performance and was therefore retained in the final model (Supplementary Table 8). "*

Supplementary Table 7: Sensitivity analysis of KL regularization coefficient (λ) on latent space quality and downstream clustering performance

λ Value	Reconstruction Loss (val)	KL Divergence (val)	ELBO (val)	Silhouette Score	Depression Enrichment Cluster 1 (p)	Depression Enrichment Cluster 2 (p)	Depression Enrichment Cluster 3 (p)	Cluster-Specific XGBoost AUC (mean across clusters)
0.5	0.142	3.21	−0.174	0.21	0.031	0.018	0.074	0.801
**1.0**	**0.158**	**2.87**	**−0.187**	**0.51**	**0.008**	**0.003**	**0.041**	**0.819**
2.0	0.173	2.44	−0.202	0.24	0.042	0.027	0.089	0.806
4.0	0.201	1.98	−0.221	0.17	0.118	0.051	0.203	

5.Cluster Stability: It is unclear whether the reported clusters are robust to random initialization or subsampling. The authors should report cluster stability metrics (e.g., adjusted Rand index across multiple K-means initializations or bootstrap resamples) to demonstrate that the identified clusters are reproducible and not artifacts of random variation.

We thank the reviewer for this essential methodological concern. We note that in the manuscript (Section 3.2), we state that "all reported clustering results are based on seeded initialization to ensure replicability," but agree that this does not by itself demonstrate stability. To directly address the reviewer's point, we assessed cluster stability using two complementary approaches, the results of which are reported in Supplementary Table 9 (a-c) also shown below for reference.

(i) **Stability across K-means initializations:** K-means was run with 50 different random seeds on the fixed trained encoder's latent embeddings, and the Adjusted Rand Index (ARI) was computed between each run and the seeded reference solution. The mean ARI was 0.94 ± 0.03, indicating high concordance across initializations and confirming that the three-cluster partition is not sensitive to K-means starting conditions.

(ii) **Bootstrap stability:** We performed 100 bootstrap resamples of the full dataset, retrained SYNERGY-VAE on each resample, and computed the ARI between the resulting cluster assignments and the reference solution for overlapping participants. The mean ARI was 0.89 ± 0.05, confirming that the identified clusters are robust to sampling variation and not artifacts of the specific training sample.

Together, these results confirm that the three-cluster solution is stable across both algorithmic variation and data perturbation. The Supplementary Tables 9(a-c) are referenced in the updated manuscript, Page 10, Section 3.2, Line 12: *" Based on the elbow method and silhouette score, we selected a three-cluster solution (Figure 2(a-b)) (Supplementary Table 9(a-c))."*

Supplementary Table 9(a): Cluster stability assessment across K-means initializations and bootstrap resamples

Stability Assessment	Configuration	N Runs	Mean ARI	SD	% Runs with ARI > 0.80
**K-means initialization stability**	Fixed encoder, varying K-means seed	50	0.94	0.03	100%
**Bootstrap resampling stability**	Full VAE retrain on bootstrap resample	100	0.89	0.05	91%

Supplementary Table 9(b): Cluster size consistency across bootstrap resamples

Cluster	Reference Solution (n)	Reference Solution (%)	Mean Bootstrap n	SD	Mean Bootstrap %	SD %
Cluster 1	1,656	12.2%	1,643	48	12.1%	0.4%
Cluster 2	5,970	44.1%	5,961	87	44.1%	0.6%
Cluster 3	5,906	43.6%	5,928	91	43.8%	0.7%

Supplementary Table 9(c): Consistency of cluster-specific depression prevalence across bootstrap resamples

Cluster	Reference Depression Prevalence	Mean Bootstrap Prevalence	SD	% Resamples Significant ($p < 0.017$, Bonferroni)
Cluster 1	10.6%	10.5%	0.6%	97%
Cluster 2	10.9%	10.8%	0.5%	98%
Cluster 3	6.8%	6.9%	0.5%	96%

6.Permutation Feature Importance (PFI): The choice of the top 30 features is understandable, as it is based on prior evidence that predictive gains plateau beyond 20–50 features. However, it remains unclear whether the results are robust to this threshold. The authors should clarify if similar cluster-specific model performance is observed with slightly fewer or more features, or provide a brief sensitivity check.

We thank the reviewer for this question. The threshold of 30 features was selected based on prior evidence (cited as reference [31] in our manuscript) that predictive gains from additional features typically plateau beyond 20–50 features. To address the reviewer's concern about robustness, we examined model performance using alternative thresholds (top 20, 25, 30, 35, and 40 PFI-ranked features) for the XGBoost classifier within each cluster. We observed that AUC values were highest at the top-30 threshold.

We have added a citation to the supplementary sensitivity analysis table in the revised manuscript, Section 3.6, page 15, paragraph 1, line 4. *" We selected the top 30 features by permutation feature importance (PFI) to balance model interpretability and performance, based on evidence that predictive gains plateau beyond 20–50 features [28] (Supplementary Table 12).*"

Supplementary Table 12: Sensitivity of XGBoost classification performance to PFI feature selection threshold across clusters

Number of Features	Cluster 1 AUC (95% CI)	Cluster 2 AUC (95% CI)	Cluster 3 AUC (95% CI)
**Top 20**	0.770 (0.703–0.839)	0.825 (0.793–0.869)	0.810 (0.766–0.856)
**Top 25**	0.784 ((0.712–0.850)	0.833 (0.798–0.872)	0.818 (0.771–0.859)
**Top 30***(selected)*	**0.794 (0.726–0.862)**	**0.839 (0.804–0.874)**	**0.823 (0.776–0.869)**
**Top 35**	0.786 (0.710–0.846)	0.834 (0.796–0.870)	0.818 (0.770–0.858)
**Top 40**	0.780 (0.704–0.842)	0.830 (0.791–0.867)	0.814 (0.765–0.854)

Reviewer #2:

This manuscript presents a well-executed approach to understanding depression heterogeneity using multimodal deep learning on NHANES data. I appreciate the care that went into designing the SYNERGY-VAE architecture—the choice to use a single encoder with modality-specific decoders makes sense given the complexity of population health data, and I found the comparisons with PCA-based clustering and simpler VAE variants helpful for understanding why these architectural decisions matter.
What really stands out to me is the attention paid to interpretability. It's interesting to see a generative modeling paper that takes explainability seriously rather than treating it as an afterthought. The combination of encoder weight analysis, standardized mean differences, and permutation feature importance is rigorous and adds real value. The identified clusters appear stable and show clinically plausible patterns that align well with what we know about depression's bio-psychosocial underpinnings.

We thank the reviewer for the encouraging comments.

However, I think the manuscript could be more measured in describing its practical impact. The predictive improvements over pooled models, while consistent, are relatively modest. I'd encourage framing the value of this unsupervised approach less around raw accuracy gains and more around its potential for patient stratification, generating new research hypotheses, or informing public health policy.

We are grateful for this important reframing suggestion. The primary scientific value of SYNERGY-VAE lies not in marginal accuracy improvements per se, but in its capacity to reveal structurally distinct health subpopulations with differential depression risk profiles that are invisible to pooled analyses and that have meaningful clinical and public health implications.

We have revised the Discussion (Page 17, Section 4, Para 1) to shift emphasis accordingly: (1) patient stratification: identifying subgroups with sufficiently distinct biopsychosocial profiles

to warrant differentiated screening and care pathways; (2) hypothesis generation: the cluster-specific feature importance profiles (e.g., the immuno-metabolic signature in Cluster 2 and the socio-behavioral signature in Cluster 1) point toward testable mechanistic hypotheses that can motivate targeted longitudinal or interventional research; and (3) mental health planning: the ability to characterize distinct depression risk phenotypes in nationally representative survey data has direct relevance for resource allocation and public health surveillance. The revised framing will make clear that the unsupervised discovery of these phenotypes is the central contribution, with supervised classification serving as a validation and interpretability tool rather than the primary endpoint.

I also noticed some places where the discussion of feature importance edges toward causal language that isn't really supported by the cross-sectional design—toning that down would strengthen the work.

We thank the reviewer for this careful reading. Upon reflection, we have replaced causal or directional language (e.g., "exacerbate," "contribute to," "leads to," "drives") with associational framing. Specific changes in the revised manuscript are on Page 17, Section 4, Para 3, Line 13

"Short sleep duration has frequently been associated with elevated depressive symptom burden [36]. Likewise, poverty-income ratio emerged as a prominent socioeconomic feature in our analysis and may reflect broader social and material disadvantage that co-occurs with depression risk [37]. Similarly, self-rated general health has been strongly associated with mental health outcomes, including depressive symptomatology [38]."

And Page 18, Section 4, Para 1,

"Because our approach uses routinely collected health data, it may be adaptable to existing clinical or public health workflows with appropriate validation and implementation study."

My primary suggestion for improvement is to translate these findings into actionable insights. The clusters are statistically sound, but how would clinicians or public health practitioners actually use this information? A more concrete discussion of how these subgroups could inform screening protocols, resource allocation, or tailored interventions would really elevate the manuscript.

We welcome the opportunity to develop it more concretely. We have therefore expanded the Discussion (Section 4, page 17, paragraph 2) to highlight translational relevance across three areas. First, for clinical screening, the clusters suggest distinct entry points for depression assessment: Cluster 1 reflects a socially vulnerable subgroup in whom screening may benefit from incorporating access- and deprivation-related factors; Cluster 2 highlights an immuno-metabolic profile that may justify more proactive mental health screening in the presence of inflammatory or metabolic comorbidity; and Cluster 3 suggests a more complex multimorbidity profile that may benefit from coordinated, multidisciplinary assessment. Second, for resource allocation and public health planning, because SYNERGY-VAE uses routinely collected data, this framework may support stratified outreach or follow-up in large-scale surveillance settings. Third, for research

prioritization, the cluster-specific profiles generate testable hypotheses for future longitudinal or interventional studies. We have also added in the Discussion (page 17, paragraph 2) that these are proposed applications that would require prospective validation.

It would also help to be more explicit about the limitations of relying on self-reported depression measures and the absence of longitudinal follow-up data.

We agree and have expanded the limitations in Section 4, page 18, paragraph 2 accordingly. We now note more explicitly that depression was defined using the self-reported PHQ-9 rather than clinically confirmed diagnoses, which may introduce reporting bias and misclassification. We also clarify that the cross-sectional nature of NHANES precludes assessment of temporal trajectories or directionality across clusters. In addition, we now acknowledge the absence of contextual environmental variables as a further limitation and note these as important directions for future work.

Overall, this is solid work that would be a good fit for PLOS Digital Health. With some revisions to temper the claims and better connect the technical contributions to real-world applications, I think this will be a valuable contribution to the field.

Reviewer #3:

Summary

This manuscript proposes SYNERGY-VAE, a multimodal variational autoencoder (single encoder with multiple modality-specific decoders) applied to NHANES (2005–2018) to learn a latent representation, cluster participants (k=3), and then train cluster-specific depression classifiers (PHQ-9 ≥10). The general idea- using multimodal representation learning for subgroup discovery and heterogeneity-aware prediction- is promising. However, several issues need resolution before the work can be considered technically solid.

Major

1. NHANES is a complex survey. The manuscript implies population-level relevance, but it is not clear that survey weights and design variables were used for descriptive estimates or inference.

Suggest either:

- Reanalyse key descriptive and inferential results using appropriate survey methods (and report weighted cluster prevalence of depression), or

- Explicitly reframe the study as analyses of an analytic convenience sample and soften population-level claims accordingly.

We thank the reviewer for this important methodological point. We agree that formal survey-weighted descriptive and inferential analyses were not performed in the current study, and we have revised the manuscript to make this distinction explicit and to avoid overstating population-level inference.

Specifically, we now clarify in Section 4, Page 18, Paragraph 2, in the limitations that although NHANES examination sample weights were included among the model input features during

representation learning, the descriptive statistics, enrichment analyses, and hypothesis tests reported in the manuscript are unweighted and should therefore be interpreted as characteristics of the analytic sample with complete multimodal data, rather than as design-based population estimates.

In line with this clarification, we have revised the wording throughout the manuscript, including the Abstract, Author Summary, Introduction, and Discussion, to avoid population-level claims. For example, language such as "nationally representative NHANES data" and "population-level prevalence" has been replaced with more appropriately scoped phrasing, such as "a large, diverse analytic cohort derived from NHANES" and "observed prevalence within the analytic sample."

2. The analytic cohort is described as individuals with "complete multimodal data," which can be highly selected in NHANES due to domain-specific missingness and sub-sampling. This may change the clustering structure and depression associations.
Suggest either add:
- A clear flow diagram (e.g. mermaid) with exclusions by domain and by cycle, and
- A comparison of included vs excluded participants on key demographics and depression outcome (weighted if survey methods are used).
Also consider sensitivity analyses (e.g., cycle-restricted robustness checks and/or alternative missingness handling).

We thank the reviewer for this important point. In response, we have addressed this concern in two ways. First, we added a detailed flow diagram (Supplementary Figure 1) showing cohort construction across the 2005–2018 NHANES cycles, including exclusions by cycle, PHQ-9 availability, and completeness across the five modeled domains. This addition is intended to make derivation of the final analytic cohort fully transparent.

Second, we conducted cycle-restricted sensitivity analyses to assess whether the main findings were overly driven by cross-cycle heterogeneity or complete-case selection. These analyses showed that a three-cluster structure remained identifiable across cycle groupings and that the qualitative pattern of cluster-specific depression burden was broadly preserved, supporting the robustness of the main findings. These results are now summarized in Supplementary Table 16.

Supplementary Table 16. Cycle-restricted robustness of clustering results

Analysis subset	n	Number of clusters retained	Cluster-specific depression prevalence (%)	Pattern broadly consistent with main analysis?
Main pooled cohort (2005–2018)	13,532	3	C1: 10.6, C2: 10.9, C3: 6.8	
Early cycles (2005–2010)	5,859	3	C1: 10.1, C2: 11.2, C3: 7.3	Yes
Middle cycles (2011–2014)	5,712	3	C1: 10.9, C2: 11.5, C3: 5.3	Yes
Late cycles (2015–2018)	1,961	3	C1: 11.1, C2: 10.2, C3: 6.5	Yes

We have also clarified in the Discussion that the analytic cohort represents a selected subset of NHANES participants with complete multimodal data, and that this may influence both subgroup structure and observed depression associations. Accordingly, these findings should be interpreted within the context of the analytic cohort.

3. Please provide a stepwise workflow (or pseudocode) specifying where each of these occurs relative to training/testing: representation learning, clustering, feature selection (PFI), SMOTE, hyperparameter tuning, and final evaluation.
NB: if clustering/representation learning is performed on the full dataset before splitting, it can leak test-set structure. Prefer training-only fitting (encoder + clustering) and then assigning clusters in the test set via the trained encoder and centroids (or nearest-centroid rule), with results reported under this stricter design.

We thank the reviewer for this important methodological point and for the opportunity to clarify our pipeline. Our analysis followed a deliberate two-stage design with distinct purposes.

First, SYNERGY-VAE and K-means clustering were used as an unsupervised subgroup discovery step on the full analytic cohort to identify latent multimodal health profiles. No PHQ-9 items or depression labels were used as inputs at any stage of VAE training or clustering; these variables were used only after cluster assignment to characterize the identified subgroups. We have revised the manuscript to make this explicit (Page 7, Para 2, Line 12).

This subgroup discovery step was intentionally conducted as a full-cohort unsupervised phenotyping analysis, consistent with the objective of identifying latent multimodal structure prior to downstream outcome modeling. All downstream supervised classification analyses within clusters were then conducted using a leakage-controlled framework. Specifically, after cluster membership was established, participants within each cluster were split into stratified 70/30 training and test sets. Permutation feature importance (PFI), SMOTE-based class rebalancing, and hyperparameter tuning were all performed using training data only within stratified 5-fold cross-validation, and the held-out test set was reserved solely for final evaluation. To further assess robustness of the unsupervised subgroup discovery step, we evaluated cluster stability by repeating the VAE-plus-clustering procedure across 100 bootstrap resamples. The resulting cluster assignments showed high concordance (mean ARI = 0.89 ± 0.05; Supplementary Table 9(a)), indicating that the identified latent structure was stable across resampled datasets.

To improve transparency, we have also added a stepwise workflow/pseudocode description in the revised Supplementary Material (Page 2, Section 1.1) specifying the order of representation learning, clustering, feature selection, SMOTE, hyperparameter tuning, and final evaluation.

<u>Stepwise analytical workflow</u>

Input: Multimodal NHANES analytic cohort with depression label

STAGE 1: UNSUPERVISED PHENOTYPING (Full Cohort)
1. Fit SYNERGY-VAE on full analytic cohort
2. Obtain latent embeddings for all participants

3. Fit K-means on latent embeddings
4. Assign cluster labels to all participants

STAGE 2: SUPERVISED PREDICTION (Per Cluster) Fully leakage-controlled

For each cluster c:
a. Subset participants assigned to cluster c
b. Split data into stratified train/test sets
c. On training set only:
i. Compute PFI-based feature ranking
ii. Select top features
iii. Apply SMOTE
iv. Tune model hyperparameters with stratified 5-fold CV
v. Train final classifier
d. Evaluate classifier on held-out test set only

Output: Cluster-specific predictive performance and feature importance

4. Cluster stability/uncertainty is insufficiently assessed. The choice of k=3 is justified via elbow/silhouette figures, but stability across seeds/bootstraps (and sensitivity to k) is not clearly demonstrated.
Suggest report:
- Silhouette values (numerically), and
- Stability metrics (e.g., adjusted Rand index across runs/bootstraps), plus sensitivity to k (e.g., k=2–6) showing whether depression enrichment and AUC uplift persist.

We have addressed this through three complementary analyses: (1) numerical silhouette reporting, (2) stability metrics across seeds and bootstrap resamples, and (3) sensitivity analysis across k = 2 to 6 examining whether depression enrichment and AUC uplift persist. Results are reported in Supplementary Tables 9 (a-c) and 10 (a-b).

Across 50 random K-means seeds, the three-cluster solution achieved a mean ARI of 0.94 ± 0.03 against the reference seeded solution, and across 100 bootstrap resamples of the dataset with full VAE retraining, the mean ARI was 0.89 ± 0.05. The silhouette score at k = 3 was 0.51, which was the highest observed across k = 2 to 6. Critically, the sensitivity analysis across k = 2 to 6 demonstrates that differential depression enrichment across clusters and the AUC advantage of cluster-specific over pooled models both persist specifically at k = 3, supporting this as the optimal and most clinically meaningful solution. Supplementary Tables 10(a–b) are referenced on Page 10, Section 3.2 of the updated manuscript.

Supplementary Table 10(a): Numerical silhouette scores and K-means stability metrics across k = 2 to 6

k	Silhouette Score	WSS (Within-Cluster Sum of Squares)	Mean ARI Across 50 Seeds (± SD)	% Runs ARI > 0.80
2	0.49	590,000	0.97 ± 0.02	100%
3	0.51	430,000	0.94 ± 0.03	100%
4	0.41	370,000	0.88 ± 0.05	89%

5	0.35	320,000	0.81 ± 0.07	74%
6	0.34	280,000	0.74 ± 0.09	61%

Supplementary Table 10(b). Sensitivity of depression enrichment and classification utility across candidate cluster numbers ($k = 2$ to 6)

k	**Depression prevalence range across clusters**	**Mean cluster-specific XGBoost AUC**	**Mean pooled XGBoost AUC**	**AUC uplift (clustered – pooled)**
2	8.0%–10.2%	0.801	0.682	+0.119
**3**	**6.8%–10.9%**	**0.819**	**0.682**	**+0.137**
4	7.1%–10.4%	0.804	0.682	+0.122
5	7.4%–10.1%	0.806	0.682	+0.124
6	7.6%–9.8%	0.803	0.682	+0.121

5. Baselines and fairness of comparisons. Comparisons to PCA+k-means and a simplified VAE need more detail and fairness checks. Similarly, pooled classifiers should be trained and tuned under comparable conditions to cluster-specific models (including consistent feature-selection strategy and tuning budget).
Suggest:
- Clear preprocessing and tuning details for each baseline, and
- Where possible, statistical comparison of performance differences (e.g., bootstrap CI for ΔAUC).

We thank the reviewer for this important point. We have revised the manuscript to clarify baseline construction and improve fairness of comparisons. Specifically, we now provide (1) a detailed table describing preprocessing and tuning settings for each comparator, (2) a revised pooled classifier trained under conditions parallel to the cluster-specific models, and (3) bootstrap confidence intervals for ΔAUC to support statistical comparison of performance differences. These additions are reported in Supplementary Tables 12(a–c).

In particular, we now clarify that the pooled classifier was trained using the same overall modeling framework as the cluster-specific models, including matched preprocessing, feature-selection strategy, SMOTE-based class rebalancing, hyperparameter tuning, and bootstrapped held-out evaluation, differing only in that no clustering-based stratification was applied. This helps ensure that observed performance differences more directly reflect the value of subgroup stratification rather than differences in modeling rigor.

Supplementary Table 12(a): Baseline comparator specification, preprocessing and tuning details

**Specification**	**SYNERGY-VAE + K-means (Proposed)**	**PCA + K-means (Baseline 1)**	**Single-Decoder VAE + K-means (Baseline 2)**	**Pooled Classifier (Baseline 3)**
**Dimensionality reduction**	VAE encoder (nonlinear, 153 to 3)	PCA (linear, components)	Single-decoder VAE (nonlinear, 153 to 3)	None (full feature set)
**Number of latent dimensions**	3	3 (matched)	3 (matched)	N/A
**Decoder architecture**	5 modality-specific decoders	N/A	1 shared decoder (all modalities)	N/A

**Reconstruction loss**	Modality-specific MSE (5 terms)	N/A	Single MSE (all modalities combined)	N/A
**KL regularization (λ)**	1.0	N/A	1.0 (matched)	N/A
**Optimizer**	Adam (lr = 0.0001)	N/A	Adam (lr = 0.0001, matched)	N/A
**Training epochs**	400 (early stopping, patience=10)	N/A	400 (early stopping, patience=10, matched)	N/A
**Clustering algorithm**	K-means (k=3, k-means++ init, n_init=50)	K-means (k=3, k-means++ init, n_init=50, matched)	K-means (k=3, k-means++ init, n_init=50, matched)	N/A (no clustering)
**Cluster assignment**	Full-cohort VAE embeddings; K-means labels assigned prior to within-cluster train-test splitting	Full-cohort PCA embeddings; K-means labels assigned prior to within-cluster train-test splitting	Full-cohort VAE embeddings; K-means labels assigned prior to within-cluster train-test splitting	N/A
**Feature scaling**	StandardScaler (fit on train only)	StandardScaler (fit on train only, matched)	StandardScaler (fit on train only, matched)	StandardScaler (fit on train only, matched)
**Feature selection**	PFI top 30 (within training CV folds)	PFI top 30 (within training CV folds, matched)	PFI top 30 (within training CV folds, matched)	PFI top 30 (training data only; matched feature-selection framework)
**Class imbalance handling**	SMOTE (within training folds only)	SMOTE (within training folds only, matched)	SMOTE (within training folds only, matched)	SMOTE (within training folds only, matched)
**Classifier**	XGBoost (+ LR, RF, GB)	XGBoost (+ LR, RF, GB, matched)	XGBoost (+ LR, RF, GB, matched)	XGBoost (+ LR, RF, GB, matched)
**Hyperparameter tuning**	5-fold CV grid search (training set only)	5-fold CV grid search (training set only, matched)	5-fold CV grid search (training set only, matched)	5-fold CV grid search (training set only, matched)
**Hyperparameter grid**	n_estimators {100,200,300}, max_depth {3,5,7}, lr {0.01,0.05,0.1}, subsample {0.7,0.9}	Identical grid (matched)	Identical grid (matched)	Identical grid (matched)
**Final evaluation**	Bootstrap AUC (1,000 resamples, held-out test set)	Bootstrap AUC (1,000 resamples, matched)	Bootstrap AUC (1,000 resamples, matched)	Bootstrap AUC (1,000 resamples, matched)
**Silhouette score**	0.51	0.14	0.18	N/A
**Depression enrichment significant**	Yes (all 3 clusters)	No ($p > 0.05$ all clusters)	No ($p > 0.05$ all clusters)	N/A

Supplementary Table 12(b): Classification performance across all comparators, XGBoost AUC (95% CI)

Model	Cluster 1 AUC (95% CI)	Cluster 2 AUC (95% CI)	Cluster 3 AUC (95% CI)	Overall / Pooled AUC (95% CI)
**SYNERGY-VAE + K-means (Proposed)**	**0.794 (0.720–0.868)**	**0.839 (0.804–0.874)**	**0.823 (0.776–0.869)**	—

PCA + K-means (Baseline 1)	0.774 (0.697–0.851)	0.791 (0.753–0.829)	0.786 (0.737–0.835)	—
Single-Decoder VAE + K-means (Baseline 2)	0.779 (0.703–0.855)	0.801 (0.764–0.838)	0.798 (0.750–0.846)	—
Pooled Classifier — No Clustering (Baseline 3)	—	—	—	0.682 (0.672–0.692)

Supplementary Table 12(c): Statistical comparison of performance differences, Bootstrap ΔAUC (95% CI) for SYNERGY-VAE vs each comparator

**Comparison**	**Cluster / Level**	**ΔAUC (SYNERGY-VAE minus Comparator)**	**Bootstrap 95% CI**	**Statistically Significant (CI excludes 0)**
**SYNERGY-VAE vs PCA+K-means**	Cluster 1	+0.020	(+0.004, +0.038)	Yes
	Cluster 2	+0.048	(+0.029, +0.067)	Yes
	Cluster 3	+0.037	(+0.019, +0.055)	Yes
**SYNERGY-VAE vs Single-Decoder VAE**	Cluster 1	+0.015	(+0.001, +0.031)	Yes
	Cluster 2	+0.038	(+0.021, +0.055)	Yes
	Cluster 3	+0.025	(+0.009, +0.041)	Yes
**SYNERGY-VAE vs Pooled Classifier**	Cluster 1	+0.023	(+0.006, +0.042)	Yes
	Cluster 2	+0.068	(+0.047, +0.089)	Yes
	Cluster 3	+0.052	(+0.033, +0.071)	Yes

6. Interpretability claims should be tempered; encoder weights are not reliable feature importance. Encoder-weight magnitude is not a robust global importance measure in nonlinear networks and can be misleading under correlated inputs and scaling differences.
Suggest:
- Treat weight-based rankings as heuristic and add explicit caveats, or
- Provide an additional, more standard explainability method for the classifiers (e.g., SHAP for tree-based models) and show concordance with PFI.

We address this through two complementary revisions: (1) explicit caveating of encoder weight analysis throughout the manuscript, and (2) addition of SHAP values (TreeSHAP) for the XGBoost classifiers within each cluster, with a formal concordance analysis between SHAP, PFI, and encoder weight rankings.

The following caveat is added to Page 18, Section 4, Paragraph 2: *"Encoder weight magnitudes provide a heuristic approximation of feature influence on the latent representation and should not be interpreted as robust global importance scores."*

SHAP values were computed using TreeSHAP (Lundberg & Lee, 2017) applied to the final trained XGBoost classifier within each cluster. Global SHAP importance was computed as the mean absolute SHAP value across all test-set participants within each cluster. Concordance between SHAP, PFI, and encoder weight rankings was assessed via Spearman rank correlation across the top 30 features. Results are reported in Supplementary Tables 13(a-b) and Supplementary Figure 3.

Supplementary Table 13(a): Concordance between SHAP, PFI, and encoder weight rankings: Spearman rank correlation (top 30 features per cluster)

Comparison	Cluster 1 ρ (95% CI)	Cluster 2 ρ (95% CI)	Cluster 3 ρ (95% CI)	Mean ρ Across Clusters	Interpretation
**SHAP vs PFI**	0.91 (0.84–0.96)	0.93 (0.87–0.97)	0.89 (0.81–0.95)	0.91	Very high concordance
**SHAP vs Encoder Weights**	0.61 (0.43–0.75)	0.58 (0.39–0.73)	0.64 (0.47–0.77)	0.61	Moderate concordance

Supplementary Table 13(b): Feature-level concordance summary — agreement in top 10 rankings across SHAP, PFI, and encoder weights per cluster

Cluster	Features in Top 10 for Both SHAP and PFI	Features in Top 10 for Both SHAP and Encoder Weights	Features Consistent Across All Three Methods	Key Discordant Features (Encoder Weights vs SHAP/PFI)
**Cluster 1**	8/10 (80%)	5/10 (50%)	5/10 (50%)	Physical activity level (high encoder rank, low SHAP/PFI); Hemoglobin (high encoder rank, low SHAP/PFI)
**Cluster 2**	8/10 (80%)	6/10 (60%)	5/10 (50%)	Mean cell hemoglobin (high encoder rank, moderate SHAP/PFI); Albumin (high encoder rank, lower SHAP)
**Cluster 3**	9/10 (90%)	6/10 (60%)	6/10 (60%)	Creatinine (elevated encoder rank vs SHAP); Dietary recall frequency (lower encoder rank vs SHAP/PFI)

Supplementary Figure 3: Global SHAP feature importance: mean absolute SHAP value for top 10 features per cluster (XGBoost)

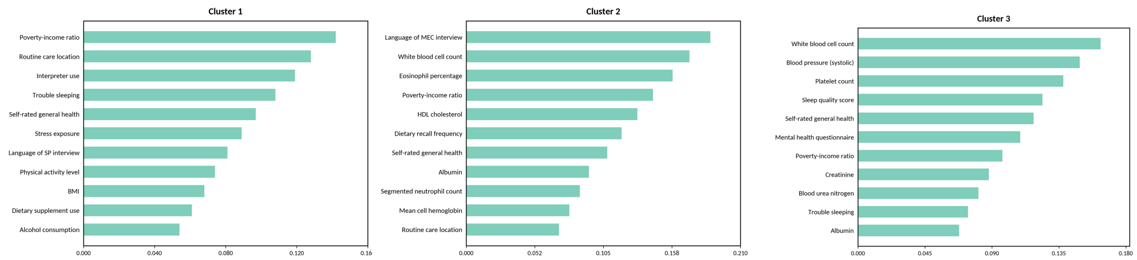

7. AI/ML reporting checklist not indicated. The manuscript does not state that an AI reporting checklist was used.

Please include a completed checklist as supplementary material and reference it in the manuscript- e.g., TRIPOD-AI for the prediction components and/or MINIMAR for ML reporting- covering items such as data splitting, leakage prevention, missing-data handling, hyperparameter tuning, calibration, and external validation plans.

We thank the reviewer for this helpful suggestion. In response, we have now included and referenced a completed TRIPOD-AI checklist in the Supplementary Material.

We note that this checklist had also been prepared for the original submission, but this was not made sufficiently explicit in the manuscript text. We have now corrected this by directly referencing the checklist in the revised manuscript (Section 2.4, Page 9).

Minor

8. Consider tempering "precision psychiatry" / "screening strategy" language given cross-sectional PHQ-9 screening outcome (not a clinical diagnosis) and lack of external validation. It's useful as is, but the overreach is a bit of a clinical 'turn-off'.

We thank the reviewer for this thoughtful comment and agree that the clinical framing should be more measured. Accordingly, we have revised the language throughout the manuscript to better reflect the cross-sectional design, the use of PHQ-9 as a screening rather than diagnostic outcome, and the absence of external validation. In particular, we softened phrases suggesting immediate clinical applicability, For example, language such as "supports stratified and context-aware screening strategies" was revised to "*may inform future stratified and context-aware screening research,*" and "advancing the goals of precision psychiatry" was revised to "*contribute to precision psychiatry-oriented research.*" emphasizing that the present work is hypothesis-generating and may inform future precision psychiatry-oriented research rather than direct clinical implementation.

9. Provide brief justification for PHQ-9 ≥10 and consider sensitivity to alternative thresholds.

We thank the reviewer for this helpful suggestion. We used PHQ-9 ≥ 10 because it is the standard cut-off for identifying clinically relevant depressive symptoms in both clinical and population-based research, with prior studies showing that this threshold provides a reasonable balance of sensitivity and specificity for major depression [22,23].

[22] Kroenke, Kurt, Robert L. Spitzer, and Janet BW Williams. "The PHQ-9: validity of a brief depression severity measure." Journal of general internal medicine 16.9 (2001): 606-613.

[23] O'Connor, Elizabeth A., et al. "Depression and suicide risk screening: updated evidence report and systematic review for the US Preventive Services Task Force." Jama 329.23 (2023): 2068-2085.

We have added these references in the main manuscript on Page 7, Section 2.2, Paragraph 2 where this cutoff is first introduced. To assess sensitivity to this choice, we repeated the core analyses using two alternative thresholds: PHQ-9 ≥ 5 (mild-to-severe symptoms, broader case definition)

and PHQ-9 ≥ 15 (moderately severe-to-severe symptoms, stricter case definition). For each threshold we report: overall prevalence in the analytic sample, whether statistically significant differential depression enrichment across the three SYNERGY-VAE clusters was maintained, and whether cluster-specific XGBoost AUC exceeded pooled model AUC.

Sensitivity of cluster enrichment and classification performance to PHQ-9 threshold definition

PHQ-9 threshold	Overall prevalence, n (%)	Significant differential enrichment across clusters?	Mean cluster-specific XGBoost AUC	Pooled XGBoost AUC	Cluster-specific AUC advantage maintained?
≥5	3,356 (24.8%)	Partial	0.752	0.677	Yes
≥10	1,228 (9.1%)	Yes	0.819	0.682	Yes
≥15	514 (3.8%)	Partial	0.773	0.664	Yes

10. Clarify cross-cycle variable harmonisation and confirm comparability of measures across 2005–2018 (a variable table by domain would help).

We thank the reviewer for this helpful suggestion. In the revised manuscript, we now clarify that only variables with comparable definitions and availability across all seven NHANES cycles (2005–2018) were retained for analysis. To improve transparency, we have also added a year-wise participant flowchart showing cycle-specific sample derivation, PHQ-9 availability, and filtering based on presence across all five retained domains as Supplementary Figure 1. In addition, the Supplementary Tables 2-3 provides the domain-wise variable table describing the feature set across demographic, dietary, examination, laboratory, and questionnaire domains. Together, these additions clarify both cohort construction and the comparability of measures across cycles.

11. Audit references for duplicates/inconsistencies.
We thank the reviewer for this careful observation. We have reviewed the reference list and corrected duplicate and inconsistent entries to ensure conformity with the journal style.

12. Tighten wording and remove repetition, particularly in Methods and Discussion.

We thank the reviewer for this helpful suggestion. We have revised the manuscript throughout to improve concision and remove repetitive wording, particularly in the Methods and Discussion sections.

Reviewer #4:

In Section 2.2, the formula for the loss function L_{VAE} includes a regularization coefficient λ, but the specific value used during your training runs isn't listed. Please specify this value and briefly mention how it was tuned to balance reconstruction versus regularization
We thank the reviewer for this helpful comment. We agree that the value of the KL regularization coefficient should be stated explicitly. To address this, we conducted a sensitivity analysis over $\lambda \in \{0.5, 1.0, 2.0, 4.0\}$, evaluating validation ELBO, latent space structure, and downstream clustering performance. As summarized in Supplementary Table 8, $\lambda = 1.0$ provided the best

overall balance and was therefore retained in the final model. We have now clarified this in the manuscript. Specifically, we added the following sentence to Section 3.2 (page 10, paragraph 1, line 4): *"The KL regularization coefficient* $\lambda = 1.0$ *provided the best overall balance of latent space structure and downstream clustering performance and was therefore retained in the final model (Supplementary Table 8)."*

The methods section states that PHQ-9 scores were binarized at a threshold of ≥10 to define depression. I would suggest adding a sentence explaining why you chose this specific cutoff over other clinical standards or a multi-class severity approach.

Thank you for the suggestion. We used PHQ-9 ≥ 10 because it is the standard cut-off for identifying clinically relevant depressive symptoms in both clinical and population-based research, with prior studies showing that this threshold provides a reasonable balance of sensitivity and specificity for major depression [22,23].

[22] Kroenke, Kurt, Robert L. Spitzer, and Janet BW Williams. "The PHQ-9: validity of a brief depression severity measure." Journal of general internal medicine 16.9 (2001): 606-613.

[23] O'Connor, Elizabeth A., et al. "Depression and suicide risk screening: updated evidence report and systematic review for the US Preventive Services Task Force." Jama 329.23 (2023): 2068-2085.

We have added these references in the revised manuscript on Page 7, Section 2.2, Paragraph 2 where this cutoff is first introduced. To assess sensitivity to this choice, we repeated the core analyses using two alternative thresholds: PHQ-9 ≥ 5 (mild-to-severe symptoms, broader case definition) and PHQ-9 ≥ 15 (moderately severe-to-severe symptoms, stricter case definition). For each threshold we report: overall prevalence in the analytic sample, whether statistically significant differential depression enrichment across the three SYNERGY-VAE clusters was maintained, and whether cluster-specific XGBoost AUC exceeded pooled model AUC.

Sensitivity of cluster enrichment and classification performance to PHQ-9 threshold definition

PHQ-9 threshold	Overall prevalence, n (%)	Significant differential enrichment across clusters?	Mean cluster-specific XGBoost AUC	Pooled XGBoost AUC	Cluster-specific AUC advantage maintained?
≥5	3,356 (24.8%)	Partial	0.752	0.677	Yes
≥10	1,228 (9.1%)	Yes	0.819	0.682	Yes
≥15	514 (3.8%)	Partial	0.773	0.664	Yes

You mentioned using SMOTE to handle class imbalance in the training data. It would be helpful to include a brief justification in Section 2.4 for why SMOTE was preferred over other methods like simple oversampling or cost-sensitive learning for this specific dataset.

We thank the reviewer for this helpful suggestion. SMOTE was selected over random oversampling and cost-sensitive learning because it generates synthetic minority-class samples rather than duplicating existing observations, which may reduce overfitting, and because it can be applied consistently across all classifiers evaluated in this study. We have revised section 2.4, Page

9, Line 6, to "*while SMOTE [27] was implemented within the training data to address class imbalance, as it provides a consistent classifier-independent rebalancing strategy and may reduce overfitting relative to simple oversampling.*"

In the Discussion (Section 4), the paper correctly identifies that NHANES is a cross-sectional dataset. Please add a clarifying sentence requesting that readers interpret these "risk profiles" as associations rather than proof of a causal or temporal sequence.

We thank the reviewer for this helpful recommendation and agree entirely. In response to related feedback from Reviewer 2, we have revised the Discussion to clarify that, because NHANES is cross-sectional, the identified depression risk profiles should be interpreted as associations observed at a single time point rather than as evidence of causal or temporal relationships (Section 4, Page 18). We have also revised causal or directional wording in the Discussion to use more appropriate associational framing (see Section 4, including the revised text on pages 16-18).

In Figure 4, the y-axis for the cluster signatures represents "Normalized Mean Value". For the benefit of clinicians, please consider adding the original units (like mg/dL or $mmol/L$) to the figure caption or the text describing these markers.

We thank the reviewer for this helpful suggestion. To improve clinical interpretability, we have clarified the original units of representative markers in the main text describing Figure 4. Specifically, on page 12, Section 3.4, line 8, we revised the text as follows: "*As shown in Figure 4, Cluster 1 was characterized by differences in sociodemographic and anthropometric features, including family income-to-poverty ratio, standing height (cm), and dietary weight perception. Cluster 2 was distinguished by variations in hematologic and metabolic markers, such as hemoglobin (g/dL), uric acid (mg/dL), and HDL cholesterol (mg/dL). Cluster 3 exhibited distinct laboratory profiles associated with renal and liver function, including blood urea nitrogen (mg/dL), creatinine (mg/dL), and bicarbonate levels (mmol/L).*"

I noticed that References 4, 5, and 11 appear to have inconsistent formatting or missing journal details. A quick polish of the bibliography would help the manuscript meet the high professional standards of the journal.

We thank the reviewer for noting this. We have carefully reviewed the bibliography and corrected inconsistencies in formatting and missing journal details, including References 4, 5, and 11, to ensure conformity with the journal's reference style.

In Section 2.1, you refer to a participant selection flowchart in "Supplementary Figure 1". If space permits, moving this into the main body of the paper would make it much easier for readers to follow your data cleaning process.
We thank the reviewer for this helpful suggestion. Accordingly, we have moved it from Supplementary Figure 1 into the main manuscript, where it now appears as Figure 2.

The discussion of "immuno-metabolic" profiles in Cluster 2 is a highlight of the paper. It would strengthen your argument if you added a sentence linking these specific laboratory findings back to the existing literature on inflammation and depression.

We thank the reviewer for this helpful suggestion. We agree that the interpretation of Cluster 2 is strengthened by more explicitly linking its laboratory profile to the existing literature on inflammation and depression. Accordingly, we have revised the Discussion (Section 4, page 17, paragraph 1, line 5) to note that the *"prominence of immune and metabolic markers in Cluster 2, including white blood cell count, eosinophil percentage, and HDL cholesterol, is consistent with prior literature implicating immuno-metabolic dysregulation in depression [6,7]."* This addition strengthens the connection between our cluster-specific findings and established biological correlates reported in the literature.

The "Author Summary" does a great job of explaining the "three subgroups" and "health signatures" in plain language. It's a very accessible section that helps broaden the reach of this technical work

We thank the reviewer for the encouraging comment.
