# Supplementary material for: SYNERGY-VAE: An explainable generative deep learning framework for discovering depression subgroups from multimodal population health data
Source: PLOS Digit Health. 2026 Sep 24;5(9):e0001719. doi: 10.1371/journal.pdig.0001719 (PMC13606994; doi:10.1371/journal.pdig.0001719)
Supplement: Supplementary file 3 [file pdig.0001719.s004.pdf]

To:
Editor in Chief, PLOS Digital Health

Dear Editor,

We thank the editors and reviewers for their constructive comments regarding our manuscript. We have addressed all comments point-by-point below (with reviewer's comment in bold). For ease of navigation, the table of contents below is hyperlinked to each reviewer response. All page and paragraph references correspond to the attached tracked version of the revised manuscript.

Table of Contents

Reviewer #1: **1**

Reviewer #2: **1**

Reviewer #4: **4**

Reviewer #1:

Excellent work from authors to address the reviewer comments with detail!

We thank the reviewer for their encouraging feedback.

Reviewer #2:

This manuscript tackles a genuinely difficult problem—depression is notoriously heterogeneous, and most large-scale epidemiological datasets were never designed with that heterogeneity in mind. The authors' decision to apply a variational autoencoder to NHANES data before clustering is sensible, and the two-stage pipeline (unsupervised representation learning followed by cluster-specific prediction) is well-conceived and cleanly executed. Importantly, the authors have not treated interpretability as an afterthought: the combination of standardized mean differences, permutation feature importance, and SHAP analysis gives the findings genuine explanatory depth, which compares favorably to many comparable studies that present clusters as outputs and call it a day.

The robustness work is also creditable. Sensitivity analyses covering KL divergence, cluster stability across initializations and bootstrap samples, and consistency under varying feature selection thresholds all add meaningful confidence. Code availability and reliance on a public dataset are further positives that should not go unmentioned.

We thank the reviewer for this positive and encouraging feedback.

That said, the manuscript's most significant weakness is not methodological—it is translational. The clusters are statistically coherent and biologically plausible, but the reader is never quite told what to do with them. How might a clinician use these subgroup profiles in practice? Do they suggest different screening approaches, different referral pathways, different targets for intervention? Right now the work reads as a technically accomplished clustering exercise, and that framing undersells what could be a more substantive contribution if the practical implications were spelled out.

We thank the reviewer for this important observation. While our previous submission had briefly noted potential practical applications of the cluster profiles in general terms, we recognized that the translational implications were not sufficiently elaborated with respect to specific screening approaches, referral pathways, and intervention targets for each subgroup. In the revised manuscript, we have substantially expanded this discussion (Section 4, page 14, paragraph 4, Lines 9-32) to address this gap directly. Specifically, we now map each cluster to concrete, cluster-specific translational implications: Cluster 1 is linked to screening frameworks embedded within safety-net and social service settings, co-located mental health referral pathways, and interventions targeting structural and socioeconomic determinants; Cluster 2 is connected to proactive depression screening triggered by abnormal metabolic and inflammatory profiles, bidirectional referral between primary care, endocrinology, and mental health services, and lifestyle-integrated intervention strategies; and Cluster 3 is associated with opportunistic screening during specialist follow-up for chronic organ disease, multidisciplinary team-based referral models, and interventions oriented toward disease burden management and psychosocial adjustment. Throughout, we have been careful to frame these implications as hypothesis-generating and contingent on prospective validation, rather than as clinical recommendations ready for immediate implementation.

There is also an interpretive issue that deserves sharper treatment. Despite appropriately softened causal language, characterizing findings as "risk profiles" and "subgroups" subtly implies stable disease phenotypes. These are associations observed at a single time point using a screening instrument—not clinically confirmed diagnoses, and not longitudinal trajectories. Precision psychiatry applications are increasingly prominent in the literature, and readers in that space may be inclined to overread what is fundamentally a cross-sectional, self-report-based analysis. The authors should be more explicit about this ceiling.

We thank the reviewer for this important clarification. We agree that the use of terms such as "risk profiles" and "subgroups," while methodologically appropriate, may inadvertently imply greater phenotypic stability than the data support. We have added explicit qualifying language to the Limitations section (Section 4, page 16, Paragraph 2, Lines 3-7) clarifying that the identified clusters reflect cross-sectional statistical associations derived from a screening instrument, do not constitute clinically confirmed diagnoses or longitudinal trajectories, and should not be overread as stable disease phenotypes. We specifically caution readers approaching this work from a precision psychiatry perspective that prospective longitudinal validation is required before any translational application can be considered.

On the epidemiological side, the decision to forgo NHANES survey weights in the primary analyses is acknowledged but not adequately interrogated. NHANES is a complex probability sample, and unweighted analyses can misrepresent population-level estimates in ways that are not always predictable in direction or magnitude. Similarly, restricting the analytic cohort to individuals with complete multimodal data is a practical necessity, but it almost certainly introduces selection pressures that affect both cluster structure and observed associations. Neither issue is fatal, but both deserve more prominent discussion than they currently receive.

We thank the reviewer for this important observation. We have expanded the Limitations section (Section 4, page 16, Paragraph 2, Lines 18–24) to more directly address the implications of not applying formal NHANES survey weights in the primary analyses, noting that NHANES oversampling of certain subgroups means that unweighted analyses may misrepresent population-level estimates in ways that are difficult to predict in direction or magnitude. We have also more explicitly addressed the selection pressures introduced by restricting the analysis to individuals with complete multimodal data across all five domains, acknowledging that individuals with missing data may differ systematically from those with complete records. We clarify that while our study demonstrates an explainable multimodal representation-learning framework within the available complete-case analytic cohort, both the omission of formal NHANES survey weights and the restriction to complete multimodal data limit generalizability beyond this analytic sample.

One smaller methodological note: encoder weight magnitudes are presented alongside SHAP values and permutation importance as evidence of feature relevance. This should be clearly labeled as a heuristic indicator. In nonlinear models with correlated inputs, weight magnitudes are a weak proxy for importance, and conflating them with more principled methods risks overstating the interpretability case.

We thank the reviewer for this note. We note that this caveat was already present in our Limitations section (Section 4, page 16, Paragraph 2, Line 14), where we stated that "encoder weight magnitudes provide a heuristic approximation of feature influence on the latent representation and should not be interpreted as robust global importance scores." However, we agree that this qualification needed to appear closer to the relevant results, and have therefore added explicit clarifying language directly in the revised manuscript's Section 3.3 (page 10) at the point where encoder weights are first presented, noting that these magnitudes serve as a heuristic indicator only and should not be treated as equivalent to the more principled PFI and SHAP analyses.

On balance, this is a solid piece of work. The technical execution is careful, the robustness analyses are genuinely strengthening, and the multimodal framing is appropriate for the scientific question. Bringing the translational discussion up to the level of the methods, and tightening the interpretive framing around the cross-sectional design, would make this a considerably stronger submission for PLOS Digital Health.

Thank you for the suggestions that helped improve the manuscript.

Reviewer #4:

I appreciate the inclusion of the detailed participant flowchart in the main text; it provides much-needed transparency regarding the exclusion criteria and the final composition of the analytic cohort.

The shift in language from "population-level" to "analytic sample" throughout the manuscript is a crucial correction that appropriately acknowledges the limitations of using unweighted survey data.

We thank the reviewer for the encouraging feedback.

While the concordance between SHAP and PFI rankings is impressively high, it might be beneficial to add a single sentence in the Discussion explaining why certain features like "Physical activity level" showed lower agreement across methods.

We thank the reviewer for this suggestion. We have added a sentence in the Discussion (Section 4, page 13, Paragraph 3, Line 6) acknowledging that features such as physical activity level showed lower cross-method agreement and explaining the methodological reasons for this pattern.

Specifically, we attribute this discordance to three interacting factors. First, self-reported physical activity measures in NHANES are known to exhibit substantial measurement error and non-linear relationships with health outcomes, which can produce differential sensitivity between permutation-based methods which assess global importance by disrupting the full marginal distribution of a feature and gradient-based methods such as SHAP which estimate local marginal contributions conditional on other features in the model [Fisher et al., 2019; Lundberg & Lee, 2017]. Second, physical activity in NHANES is documented to be collinear with BMI, dietary intake, and metabolic markers [Troiano et al., 2008], and permutation importance is known to be unstable for correlated features because permuting one correlated feature leaves its variance partially explained by its correlates, artificially deflating its measured importance relative to SHAP which conditions on the full feature set [Strobl et al., 2008]. Third, non-monotonic relationships, where moderate physical activity predicts different outcomes than either low or high activity are captured differently by tree-based SHAP values, which track local interactions, versus permutation importance, which averages over the global prediction surface [Molnar, 2020]. The combination of these factors makes physical activity a particularly unstable feature for cross-method concordance in this dataset, and we have framed it as such in the revised Discussion rather than treating the discordance as an anomaly requiring explanation by a single cause.

The references supporting this explanation are:

Fisher A, Rudin C, Dominici F. All models are wrong, but many are useful: learning a variable's importance by studying an entire class of prediction models simultaneously. Journal of Machine Learning Research. 2019;20(177):1-81.

Lundberg SM, Lee SI. A unified approach to interpreting model predictions. Advances in Neural Information Processing Systems. 2017;30:4765-4774.

Strobl C, Boulesteix AL, Kneib T, Augustin T, Zeileis A. Conditional variable importance for random forests. BMC Bioinformatics. 2008;9:307.

Troiano RP, Berrigan D, Dodd KW, Mâsse LC, Tilert T, McDowell M. Physical activity in the United States measured by accelerometer. Medicine and Science in Sports and Exercise. 2008;40(1):181-188.

Molnar C. Interpretable Machine Learning: A Guide for Making Black Box Models Explainable. 2nd ed. 2020. Available at: https://christophm.github.io/interpretable-ml-book/

The addition of the bootstrap stability analysis and Adjusted Rand Index (ARI) metrics provides strong evidence that the three-cluster solution is robust and not a result of random initialization

We thank the reviewer for this positive feedback. The bootstrap stability analysis was designed specifically to address concerns about K-means sensitivity to initialization, and the strong ARI values across 50 random seeds and 100 resamples provide the robustness evidence we consider essential for a clustering result intended to support clinical interpretation.
