# Supplementary material for: SYNERGY-VAE: An explainable generative deep learning framework for discovering depression subgroups from multimodal population health data
Source: PLOS Digit Health. 2026 Sep 24;5(9):e0001719. doi: 10.1371/journal.pdig.0001719 (PMC13606994; doi:10.1371/journal.pdig.0001719)
Supplement: Supplementary file 4 [file pdig.0001719.s005.pdf]

To:
Editor in Chief, PLOS Digital Health

Dear Editor,

We thank the editors and reviewers for their constructive comments regarding our manuscript. We have addressed all comments point-by-point below (with reviewer's comment in bold). All page and paragraph references correspond to the attached tracked version of the revised manuscript.

Reviewer #2:

This is a strong and carefully executed manuscript. The updated SYNERGY-VAE framework is a reasonable and interesting way to integrate multiple NHANES data domains, derive latent subgroups, and then examine cluster-specific predictors. I recommend minor revision. The technical work is generally convincing, and the manuscript appears much improved. The remaining issues are mostly about interpretation, transparency, and clinical framing.

We thank the reviewer for the constructive assessment of our revised manuscript. We have addressed the remaining points through targeted revisions focused on translational framing, statistical transparency, interpretability, and limitations, as detailed below.

Comment 1: The translational discussion should be strengthened. Each cluster should be linked to a concrete next step, including where screening would occur, what would trigger screening, what referral pathway would follow, and what prospective study would be needed. The clusters should not be presented as clinical phenotypes until prospectively validated.

We thank the reviewer for this important suggestion. We have revised the Discussion section (Page 14, Paragraph 1) to make the translational agenda more concrete and appropriately cautious. Specifically, we now link each latent subgroup to a potential screening setting, screening trigger, referral pathway, and prospective validation strategy. For Cluster 1, we discuss screening in primary care, community health, or social-service-linked settings, triggered by socio-behavioral and healthcare access signals, with referral pathways that integrate mental health assessment with social work or navigation support. For Cluster 2, we discuss screening in primary care or cardiometabolic/endocrinology settings, triggered by immuno-metabolic markers, with bidirectional referral between metabolic care and mental health services. For Cluster 3, we discuss opportunistic screening in nephrology, hepatology, or complex chronic disease clinics, with referral to multidisciplinary care models that include behavioral health support.

We have also softened the clinical language in the manuscript. The clusters are now framed as hypothesis-generating latent subgroups derived from cross-sectional NHANES data, rather than clinically validated depression phenotypes or subtypes. We explicitly state that prospective longitudinal validation would be required to test cluster stability, temporal association with depression outcomes, and clinical usefulness before translation into practice (Page 14, Paragraph 1, Lines 30-35).

Comment 2: The comparison between cluster-specific and pooled models needs more detail. The authors should clarify the feature set used for pooled models and test whether AUC differences are statistically meaningful using DeLong's test or bootstrap confidence intervals. If differences are small or not significant, language should be softened.

We thank the reviewer for this helpful suggestion. We now clarify that the pooled XGBoost model was trained once on the full unclustered training cohort using the same preprocessing, SMOTE-based class rebalancing, classifier type, and PFI-based feature-selection workflow as the cluster-specific models. The pooled model used the top 30 PFI-ranked features selected from the full training cohort, whereas the cluster-specific models used the top 30 PFI-ranked features selected within each latent cluster.

In addition, we have now performed a formal bootstrap comparison of AUC differences between the cluster-specific XGBoost models and the pooled XGBoost model. To ensure a fair comparison, the pooled model was evaluated within the same held-out test participants assigned to each latent cluster, and ΔAUC was calculated as AUC_cluster-specific − AUC_pooled. Bootstrap 95% confidence intervals were estimated using 1,000 resamples of paired held-out predictions within each cluster. These results are now reported in Supplementary Table 18 (referenced in the updated manuscript in Section 2.4, page 8).

Supplementary Table 18. Bootstrap comparison of cluster-specific XGBoost models versus pooled XGBoost model

Cluster	Cluster-specific model AUC	Pooled model AUC evaluated in same cluster test subset	ΔAUC	Bootstrap 95% CI for ΔAUC	Statistically significant?
Cluster 1	0.794 (0.720 - 0.868)	0.705 (0.683 - 0.719)	+0.089	(0.021, 0.154)	Yes
Cluster 2	0.839 (0.804 - 0.874)	0.774 (0.735 - 0.798)	+0.065	(0.024, 0.108)	Yes
Cluster 3	0.823 (0.776 - 0.869)	0.761 (0.724 - 0.787)	+0.062	(0.013, 0.111)	Yes

The cluster-specific models showed higher AUCs than the pooled model across all three clusters, with ΔAUCs of +0.089, +0.065, and +0.062 for Clusters 1-3, respectively. The corresponding bootstrap confidence intervals excluded zero, supporting statistically meaningful improvement with cluster-specific modeling.

Comment 3: The SMD results should include Benjamini-Hochberg q-values for the top features, at least in the supplement. The authors should state the actual criterion used to decide that performance had plateaued for the top 30 PFI features. The encoder-weight analysis should be clearly labelled as heuristic, including in the figure caption.

We thank the reviewer for these helpful suggestions. We have updated the supplementary material to include Benjamini-Hochberg adjusted q-values for the top 10 SMD-ranked features in each cluster (Supplementary Table 17).

We have also clarified the criterion used to select the top 30 PFI features. Specifically, we evaluated classifier performance across increasing numbers of PFI-ranked features (Top 20, Top 25, Top 30(selected), Top 35, Top 40) (please refer to Supplementary Table 11) and selected the top 30 features because performance gains beyond this point were minimal, while the model remained more interpretable than larger feature sets.

Supplementary Table 11: Sensitivity of XGBoost classification performance to PFI feature selection threshold across clusters

**Number of Features**	**Cluster 1 AUC (95% CI)**	**Cluster 2 AUC (95% CI)**	**Cluster 3 AUC (95% CI)**
**Top 20**	0.770 (0.703–0.839)	0.825 (0.793–0.869)	0.810 (0.766–0.856)
**Top 25**	0.784 ((0.712–0.850)	0.833 (0.798–0.872)	0.818 (0.771–0.859)
**Top 30***(selected)*	**0.794 (0.720–0.868)**	**0.839 (0.804–0.874)**	**0.823 (0.776–0.869)**
**Top 35**	0.786 (0.710–0.846)	0.834 (0.796–0.870)	0.818 (0.770–0.858)
**Top 40**	0.780 (0.704–0.842)	0.830 (0.791–0.867)	0.814 (0.765–0.854)

This Supplementary table is referenced in Page 10, Section 3.6, Paragraph 1 of the manuscript.

Finally, we have revised the encoder-weight analysis text (Page 10, Section 3.3) and Figure 4 caption to explicitly label "*To note, encoder weight magnitudes should be interpreted as a heuristic representation-level diagnostic, not as formal feature-importance estimates or evidence of causal relevance.*"

Comment 4: The SHAP analysis deserves slightly more attention in the main text. A short summary of whether it supports the PFI findings would help.

We agree and have expanded the main-text discussion of SHAP. We now briefly summarize how SHAP supports and complements the PFI findings in two places: Section 3.6, Page 13, Paragraph 4 and Section 4, Page 14, Paragraph 2. In particular, we note that several features identified by PFI also showed high SHAP contribution, supporting the robustness of key predictors across interpretability methods. At the same time, SHAP provided additional insight into the direction and heterogeneity of feature effects within cluster-specific XGBoost models.

Comment 5: The limitations should be stated more plainly. The unweighted NHANES analysis may affect population-level interpretation, and the direction of bias is not obvious. Complete-case analysis may introduce selection bias; if possible, included and excluded participants should be compared. The manuscript should be explicit that the framework would need to be retrained or adapted before use in other survey or clinical datasets.

We thank the reviewer for this important point. We have revised the limitations section to state these issues more plainly (Section 4, Page 16, Paragraph 3). Specifically, we now clarify that the unweighted NHANES analysis limits population-level interpretation and that the direction of bias is not necessarily predictable. We also emphasize that complete-case inclusion may introduce selection bias and note that future work should compare included and excluded participants to better quantify this issue. Finally, we have added an explicit statement that SYNERGY-VAE was trained and evaluated within the harmonized NHANES 2005–2018 analytic cohort and would

require dataset-specific feature mapping, preprocessing, retraining or recalibration, and independent validation before being applied to other survey or clinical datasets.
