# Supplementary material for: SYNERGY-VAE: An explainable generative deep learning framework for discovering depression subgroups from multimodal population health data
Source: PLOS Digit Health. 2026 Sep 24;5(9):e0001719. doi: 10.1371/journal.pdig.0001719 (PMC13606994; doi:10.1371/journal.pdig.0001719)
Supplement: Peer review history

# Peer review: Decision Letter 0

**DOI:** 10.1371/journal.pdig.0001719.r001

**Authors:** Nadav Rappoport, Jing Tian

4 Feb 2026

Response to Reviewers Revised Manuscript with Track Changes Manuscript **Journal Requirements:**

1. We ask that a manuscript source file is provided at Revision. Please upload your manuscript file as a .doc, .docx, .rtf or .tex.

2. We have noticed that you have uploaded Supporting Information files, but you have not included a list of legends. Please add a full list of legends for your Supporting Information files after the references list.

**Additional Editor Comments (if provided):** **Reviewers' Comments:**

**Comments to the Author**

1. Does this manuscript meet PLOS Digital Health’s publication criteria?

Reviewer #1: Yes

Reviewer #2: Partly

Reviewer #3: Partly

Reviewer #4: Yes

**********

2. Has the statistical analysis been performed appropriately and rigorously?

Reviewer #1: Yes

Reviewer #2: Yes

Reviewer #3: No

Reviewer #4: Yes

**********

3. Have the authors made all data underlying the findings in their manuscript fully available (please refer to the Data Availability Statement at the start of the manuscript PDF file)?

The PLOS Data policy

Reviewer #1: Yes

Reviewer #2: Yes

Reviewer #3: Yes

Reviewer #4: Yes

**********

4. Is the manuscript presented in an intelligible fashion and written in standard English?

Reviewer #1: Yes

Reviewer #2: Yes

Reviewer #3: Yes

Reviewer #4: Yes

**********

Reviewer #1: This study presents SYNERGY-VAE, a multimodal variational autoencoder integrating demographic, dietary, laboratory, examination, and questionnaire data from NHANES to identify latent subgroups associated with depression risk. The authors demonstrate that their framework uncovers clinically meaningful clusters with distinct biopsychosocial profiles and varying depression prevalence. Feature interpretability is enhanced through encoder weights, standardized mean differences, and permutation feature importance, highlighting relevant socioeconomic, physiological, and behavioral factors.

This is a well-conducted and innovative study. The manuscript demonstrates thoughtful methodology, interpretable analyses, and clear clinical relevance. However, a few methodological clarifications can strengthen the findings:

1.Clustering Methodology: Please clarify the rationale for using K-means rather than alternative clustering methods such as Gaussian Mixture Models, hierarchical clustering, or density-based approaches (e.g., HDBSCAN), which may capture different structures in the latent space.

2.PHQ-9 Inclusion in VAE: It is unclear whether depression-related questionnaire items (e.g., PHQ-9 components) were included as inputs to the VAE. This clarification is essential to support claims that the subgroup discovery is unbiased with respect to depression status.

3.Statistical Correction: Fisher’s exact tests were used to assess enrichment of depression across clusters, but it is not stated whether multiple testing corrections were applied. The authors should clarify this to ensure appropriate statistical interpretation.

4.KL Regularization (λ): The manuscript does not specify how the KL regularization coefficient (λ) was selected or tuned. Further explanation will help, as λ affects latent space regularization, cluster separability, and downstream interpretability.

5.Cluster Stability: It is unclear whether the reported clusters are robust to random initialization or subsampling. The authors should report cluster stability metrics (e.g., adjusted Rand index across multiple K-means initializations or bootstrap resamples) to demonstrate that the identified clusters are reproducible and not artifacts of random variation.

6.Permutation Feature Importance (PFI): The choice of the top 30 features is understandable, as it is based on prior evidence that predictive gains plateau beyond 20–50 features. However, it remains unclear whether the results are robust to this threshold. The authors should clarify if similar cluster-specific model performance is observed with slightly fewer or more features, or provide a brief sensitivity check.

Reviewer #2: This manuscript presents a well-executed approach to understanding depression heterogeneity using multimodal deep learning on NHANES data. I appreciate the care that went into designing the SYNERGY-VAE architecture—the choice to use a single encoder with modality-specific decoders makes sense given the complexity of population health data, and I found the comparisons with PCA-based clustering and simpler VAE variants helpful for understanding why these architectural decisions matter.

What really stands out to me is the attention paid to interpretability. It's interesting to see a generative modeling paper that takes explainability seriously rather than treating it as an afterthought. The combination of encoder weight analysis, standardized mean differences, and permutation feature importance is rigorous and adds real value. The identified clusters appear stable and show clinically plausible patterns that align well with what we know about depression's bio-psychosocial underpinnings.

However, I think the manuscript could be more measured in describing its practical impact. The predictive improvements over pooled models, while consistent, are relatively modest. I'd encourage framing the value of this unsupervised approach less around raw accuracy gains and more around its potential for patient stratification, generating new research hypotheses, or informing public health policy. I also noticed some places where the discussion of feature importance edges toward causal language that isn't really supported by the cross-sectional design—toning that down would strengthen the work.

My primary suggestion for improvement is to translate these findings into actionable insights. The clusters are statistically sound, but how would clinicians or public health practitioners actually use this information? A more concrete discussion of how these subgroups could inform screening protocols, resource allocation, or tailored interventions would really elevate the manuscript. It would also help to be more explicit about the limitations of relying on self-reported depression measures and the absence of longitudinal follow-up data.

Overall, this is solid work that would be a good fit for PLOS Digital Health. With some revisions to temper the claims and better connect the technical contributions to real-world applications, I think this will be a valuable contribution to the field.

Reviewer #3: Summary

This manuscript proposes SYNERGY-VAE, a multimodal variational autoencoder (single encoder with multiple modality-specific decoders) applied to NHANES (2005–2018) to learn a latent representation, cluster participants (k=3), and then train cluster-specific depression classifiers (PHQ-9 ≥10). The general idea- using multimodal representation learning for subgroup discovery and heterogeneity-aware prediction- is promising. However, several issues need resolution before the work can be considered technically solid.

Major

1. NHANES is a complex survey. The manuscript implies population-level relevance, but it is not clear that survey weights and design variables were used for descriptive estimates or inference.

Suggest either:

- Reanalyse key descriptive and inferential results using appropriate survey methods (and report weighted cluster prevalence of depression), or

- Explicitly reframe the study as analyses of an analytic convenience sample and soften population-level claims accordingly.

2. The analytic cohort is described as individuals with “complete multimodal data,” which can be highly selected in NHANES due to domain-specific missingness and sub-sampling. This may change the clustering structure and depression associations.

Suggest either add:

- A clear flow diagram (e.g. mermaid) with exclusions by domain and by cycle, and

- A comparison of included vs excluded participants on key demographics and depression outcome (weighted if survey methods are used).

Also consider sensitivity analyses (e.g., cycle-restricted robustness checks and/or alternative missingness handling).

3. Please provide a stepwise workflow (or pseudocode) specifying where each of these occurs relative to training/testing: representation learning, clustering, feature selection (PFI), SMOTE, hyperparameter tuning, and final evaluation.

NB: if clustering/representation learning is performed on the full dataset before splitting, it can leak test-set structure. Prefer training-only fitting (encoder + clustering) and then assigning clusters in the test set via the trained encoder and centroids (or nearest-centroid rule), with results reported under this stricter design.

4. Cluster stability/uncertainty is insufficiently assessed. The choice of k=3 is justified via elbow/silhouette figures, but stability across seeds/bootstraps (and sensitivity to k) is not clearly demonstrated.

Suggest report:

- Silhouette values (numerically), and

- Stability metrics (e.g., adjusted Rand index across runs/bootstraps), plus sensitivity to k (e.g., k=2–6) showing whether depression enrichment and AUC uplift persist.

5. Baselines and fairness of comparisons. Comparisons to PCA+k-means and a simplified VAE need more detail and fairness checks. Similarly, pooled classifiers should be trained and tuned under comparable conditions to cluster-specific models (including consistent feature-selection strategy and tuning budget).

Suggest:

- Clear preprocessing and tuning details for each baseline, and

- Where possible, statistical comparison of performance differences (e.g., bootstrap CI for ΔAUC).

6. Interpretability claims should be tempered; encoder weights are not reliable feature importance. Encoder-weight magnitude is not a robust global importance measure in nonlinear networks and can be misleading under correlated inputs and scaling differences.

Suggest:

- Treat weight-based rankings as heuristic and add explicit caveats, or

- Provide an additional, more standard explainability method for the classifiers (e.g., SHAP for tree-based models) and show concordance with PFI.

7. AI/ML reporting checklist not indicated. The manuscript does not state that an AI reporting checklist was used.

Please include a completed checklist as supplementary material and reference it in the manuscript- e.g., TRIPOD-AI for the prediction components and/or MINIMAR for ML reporting- covering items such as data splitting, leakage prevention, missing-data handling, hyperparameter tuning, calibration, and external validation plans.

Minor

8. Consider tempering “precision psychiatry” / “screening strategy” language given cross-sectional PHQ-9 screening outcome (not a clinical diagnosis) and lack of external validation. It's useful as is, but the overreach is a bit of a clinical 'turn-off'.

9. Provide brief justification for PHQ-9 ≥10 and consider sensitivity to alternative thresholds.

10. Clarify cross-cycle variable harmonisation and confirm comparability of measures across 2005–2018 (a variable table by domain would help).

11. Audit references for duplicates/inconsistencies.

12. Tighten wording and remove repetition, particularly in Methods and Discussion.

Reviewer #4: In Section 2.2, the formula for the loss function $L_{VAE}$ includes a regularization coefficient $\lambda$, but the specific value used during your training runs isn't listed. Please specify this value and briefly mention how it was tuned to balance reconstruction versus regularization

The methods section states that PHQ-9 scores were binarized at a threshold of $\ge10$ to define depression. I would suggest adding a sentence explaining why you chose this specific cutoff over other clinical standards or a multi-class severity approach.

You mentioned using SMOTE to handle class imbalance in the training data. It would be helpful to include a brief justification in Section 2.4 for why SMOTE was preferred over other methods like simple oversampling or cost-sensitive learning for this specific dataset.

In the Discussion (Section 4), the paper correctly identifies that NHANES is a cross-sectional dataset. Please add a clarifying sentence requesting that readers interpret these "risk profiles" as associations rather than proof of a causal or temporal sequence.

In Figure 4, the y-axis for the cluster signatures represents "Normalized Mean Value". For the benefit of clinicians, please consider adding the original units (like $mg/dL$ or $mmol/L$) to the figure caption or the text describing these markers.

I noticed that References 4, 5, and 11 appear to have inconsistent formatting or missing journal details. A quick polish of the bibliography would help the manuscript meet the high professional standards of the journal.

In Section 2.1, you refer to a participant selection flowchart in "Supplementary Figure 1". If space permits, moving this into the main body of the paper would make it much easier for readers to follow your data cleaning process.

The discussion of "immuno-metabolic" profiles in Cluster 2 is a highlight of the paper. It would strengthen your argument if you added a sentence linking these specific laboratory findings back to the existing literature on inflammation and depression.

The "Author Summary" does a great job of explaining the "three subgroups" and "health signatures" in plain language. It's a very accessible section that helps broaden the reach of this technical work

**********

what does this mean?). If published, this will include your full peer review and any attached files.

**Do you want your identity to be public for this peer review?**  If you choose “no”, your identity will remain anonymous but your review may still be made public.

For information about this choice, including consent withdrawal, please see our Privacy Policy

Reviewer #1: **Yes:**  Parvati Naliyatthaliyazchayil

Reviewer #2: **Yes:**  aasim ayaz wani

Reviewer #3: **Yes:**  Dylan A Mordaunt

Reviewer #4: **Yes:**  Bala Nimmana

**********

**Figure resubmission:**

**Reproducibility:**

To enhance the reproducibility of your results, we recommend that authors of applicable studies deposit laboratory protocols in protocols.io, where a protocol can be assigned its own identifier (DOI) such that it can be cited independently in the future. Additionally, PLOS ONE offers an option to publish peer-reviewed clinical study protocols. Read more information on sharing protocols at https://plos.org/protocols?utm_medium=editorial-email&utm_source=authorletters&utm_campaign=protocols

# Author comment: Author response to Decision Letter 1

**DOI:** 10.1371/journal.pdig.0001719.r002

6 Apr 2026

Attachment Submitted filename: Response to Reviewers.pdf

# Peer review: Decision Letter 1

**DOI:** 10.1371/journal.pdig.0001719.r003

**Authors:** Nadav Rappoport, Jing Tian

29 May 2026

Response to ReviewersRevised Manuscript with Track ChangesManuscript**Journal Requirements:** **Additional Editor Comments (if provided):** **Reviewers' Comments:**

**Comments to the Author**

Reviewer #1: All comments have been addressed

Reviewer #2: All comments have been addressed

Reviewer #4: All comments have been addressed

**********

publication criteria?

Reviewer #1: Yes

Reviewer #2: Partly

Reviewer #4: Yes

**********

3. Has the statistical analysis been performed appropriately and rigorously?

Reviewer #1: Yes

Reviewer #2: No

Reviewer #4: Yes

**********

4. Have the authors made all data underlying the findings in their manuscript fully available (please refer to the Data Availability Statement at the start of the manuscript PDF file)?

The PLOS Data policy

Reviewer #1: Yes

Reviewer #2: Yes

Reviewer #4: Yes

**********

5. Is the manuscript presented in an intelligible fashion and written in standard English?

Reviewer #1: Yes

Reviewer #2: Yes

Reviewer #4: Yes

**********

Reviewer #1: Excellent work from authors to address the reviewer comments with detail!

Reviewer #2: This manuscript tackles a genuinely difficult problem—depression is notoriously heterogeneous, and most large-scale epidemiological datasets were never designed with that heterogeneity in mind. The authors' decision to apply a variational autoencoder to NHANES data before clustering is sensible, and the two-stage pipeline (unsupervised representation learning followed by cluster-specific prediction) is well-conceived and cleanly executed. Importantly, the authors have not treated interpretability as an afterthought: the combination of standardized mean differences, permutation feature importance, and SHAP analysis gives the findings genuine explanatory depth, which compares favorably to many comparable studies that present clusters as outputs and call it a day.

The robustness work is also creditable. Sensitivity analyses covering KL divergence, cluster stability across initializations and bootstrap samples, and consistency under varying feature selection thresholds all add meaningful confidence. Code availability and reliance on a public dataset are further positives that should not go unmentioned.

That said, the manuscript's most significant weakness is not methodological—it is translational. The clusters are statistically coherent and biologically plausible, but the reader is never quite told what to do with them. How might a clinician use these subgroup profiles in practice? Do they suggest different screening approaches, different referral pathways, different targets for intervention? Right now the work reads as a technically accomplished clustering exercise, and that framing undersells what could be a more substantive contribution if the practical implications were spelled out.

There is also an interpretive issue that deserves sharper treatment. Despite appropriately softened causal language, characterizing findings as "risk profiles" and "subgroups" subtly implies stable disease phenotypes. These are associations observed at a single time point using a screening instrument—not clinically confirmed diagnoses, and not longitudinal trajectories. Precision psychiatry applications are increasingly prominent in the literature, and readers in that space may be inclined to overread what is fundamentally a cross-sectional, self-report-based analysis. The authors should be more explicit about this ceiling.

On the epidemiological side, the decision to forgo NHANES survey weights in the primary analyses is acknowledged but not adequately interrogated. NHANES is a complex probability sample, and unweighted analyses can misrepresent population-level estimates in ways that are not always predictable in direction or magnitude. Similarly, restricting the analytic cohort to individuals with complete multimodal data is a practical necessity, but it almost certainly introduces selection pressures that affect both cluster structure and observed associations. Neither issue is fatal, but both deserve more prominent discussion than they currently receive.

One smaller methodological note: encoder weight magnitudes are presented alongside SHAP values and permutation importance as evidence of feature relevance. This should be clearly labeled as a heuristic indicator. In nonlinear models with correlated inputs, weight magnitudes are a weak proxy for importance, and conflating them with more principled methods risks overstating the interpretability case.

On balance, this is a solid piece of work. The technical execution is careful, the robustness analyses are genuinely strengthening, and the multimodal framing is appropriate for the scientific question. Bringing the translational discussion up to the level of the methods, and tightening the interpretive framing around the cross-sectional design, would make this a considerably stronger submission for PLOS Digital Health.

Reviewer #4: I appreciate the inclusion of the detailed participant flowchart in the main text; it provides much-needed transparency regarding the exclusion criteria and the final composition of the analytic cohort.

The shift in language from "population-level" to "analytic sample" throughout the manuscript is a crucial correction that appropriately acknowledges the limitations of using unweighted survey data.

While the concordance between SHAP and PFI rankings is impressively high, it might be beneficial to add a single sentence in the Discussion explaining why certain features like "Physical activity level" showed lower agreement across methods.

The addition of the bootstrap stability analysis and Adjusted Rand Index (ARI) metrics provides strong evidence that the three-cluster solution is robust and not a result of random initialization

**********

what does this mean?). If published, this will include your full peer review and any attached files.

**Do you want your identity to be public for this peer review?**  If you choose “no”, your identity will remain anonymous but your review may still be made public.

For information about this choice, including consent withdrawal, please see our Privacy Policy

Reviewer #1: **Yes:**  Parvati Naliyatthaliyazchayil

Reviewer #2: **Yes:**  Aasim Ayaz Wani

Reviewer #4: **Yes:**  Bala Nimmana

**********

**Figure resubmission:**

**Reproducibility:**

To enhance the reproducibility of your results, we recommend that authors of applicable studies deposit laboratory protocols in protocols.io, where a protocol can be assigned its own identifier (DOI) such that it can be cited independently in the future. Additionally, PLOS ONE offers an option to publish peer-reviewed clinical study protocols. Read more information on sharing protocols at https://plos.org/protocols?utm_medium=editorial-email&utm_source=authorletters&utm_campaign=protocols

# Author comment: Author response to Decision Letter 2

**DOI:** 10.1371/journal.pdig.0001719.r004

18 Jun 2026

Attachment Submitted filename: Reviewer_Response2.pdf

# Peer review: Decision Letter 2

**DOI:** 10.1371/journal.pdig.0001719.r005

**Authors:** Nadav Rappoport, Jing Tian

7 Jul 2026

Response to Reviewers Revised Manuscript with Track Changes Manuscript **Journal Requirements:**

**Additional Editor Comments (if provided):** **Reviewers' Comments:**

**Comments to the Author**

Reviewer #2: All comments have been addressed

**********

publication criteria?

Reviewer #2: Yes

**********

3. Has the statistical analysis been performed appropriately and rigorously?

Reviewer #2: No

**********

4. Have the authors made all data underlying the findings in their manuscript fully available (please refer to the Data Availability Statement at the start of the manuscript PDF file)?

The PLOS Data policy

Reviewer #2: Yes

**********

5. Is the manuscript presented in an intelligible fashion and written in standard English?

Reviewer #2: Yes

**********

**Reviewer #2:**  This is a strong and carefully executed manuscript. The updated SYNERGY-VAE framework is a reasonable and interesting way to integrate multiple NHANES data domains, derive latent subgroups, and then examine cluster-specific predictors. I recommend minor revision. The technical work is generally convincing, and the manuscript appears much improved. The remaining issues are mostly about interpretation, transparency, and clinical framing.

The main point I would ask the authors to strengthen is the translational discussion. The current version gives plausible clinical scenarios for the clusters, but these still read more like possibilities than a research plan. The paper would be stronger if each cluster were linked to a concrete next step: where screening would occur, what would trigger screening, what referral pathway would follow, and what kind of prospective study would be needed to test clinical usefulness. This is especially important because the data are cross-sectional. The clusters should not be presented as clinical phenotypes until they have been prospectively validated.

The comparison between cluster-specific and pooled models also needs a little more detail. The authors should clarify exactly what feature set was used for the pooled models and should test whether the reported AUC differences are statistically meaningful, using DeLong’s test or bootstrap confidence intervals. If the differences are small or not significant, the language around the advantage of clustering should be softened.

The interpretability analysis is a strength of the paper, but a few details should be made clearer. The SMD results should include the Benjamini-Hochberg q-values for the top features, at least in the supplement. The choice of the top 30 PFI features is reasonable, but the authors should state the actual criterion used to decide that performance had plateaued. The encoder-weight analysis should also be clearly labelled as heuristic, including in the figure caption, since readers may otherwise treat it as a formal importance measure.

The SHAP analysis deserves slightly more attention in the main text. Since SHAP is familiar to many readers, a short summary of whether it supports the PFI findings would help. The authors do not need to over-expand this section, but they should tell the reader what the SHAP analysis adds.

A few limitations should be stated more plainly. The unweighted NHANES analysis may affect population-level interpretation, and the direction of that bias is not obvious. Complete-case analysis may also introduce selection bias; if possible, the authors should briefly compare included and excluded participants. Finally, the manuscript should be explicit that the framework would need to be retrained or adapted before use in other survey or clinical datasets.

Overall, this is a well-done study and a useful contribution to multimodal mental health analytics. The manuscript does not need major restructuring. It needs a more concrete translational agenda, clearer statistical comparison with pooled models, and a few additional details around interpretability and limitations. Once those points are addressed, the paper should be suitable for publication.

**********

what does this mean?). If published, this will include your full peer review and any attached files.

**Do you want your identity to be public for this peer review?**  If you choose “no”, your identity will remain anonymous but your review may still be made public.

For information about this choice, including consent withdrawal, please see our Privacy Policy

Reviewer #2: **Yes:**  Aasim Ayaz Wani

**********

**Figure resubmission:**

**Reproducibility:**

To enhance the reproducibility of your results, we recommend that authors of applicable studies deposit laboratory protocols in protocols.io, where a protocol can be assigned its own identifier (DOI) such that it can be cited independently in the future. Additionally, PLOS ONE offers an option to publish peer-reviewed clinical study protocols. Read more information on sharing protocols at https://plos.org/protocols?utm_medium=editorial-email&utm_source=authorletters&utm_campaign=protocols

# Author comment: Author response to Decision Letter 3

**DOI:** 10.1371/journal.pdig.0001719.r006

5 Aug 2026

Attachment Submitted filename: Response_to_Reviewers_auresp_3.pdf

# Peer review: Decision Letter 3

**DOI:** 10.1371/journal.pdig.0001719.r007

**Authors:** Jing Tian, Tim M.H. Li

25 Aug 2026

SYNERGY-VAE: An Explainable Generative Deep Learning Framework for Discovering Depression Subgroups from Multimodal Population Health Data

PDIG-D-25-01002R3

Dear Dr Sharma,

We are pleased to inform you that your manuscript 'SYNERGY-VAE: An Explainable Generative Deep Learning Framework for Discovering Depression Subgroups from Multimodal Population Health Data' has been provisionally accepted for publication in PLOS Digital Health.

Before your manuscript can be formally accepted you will need to complete some formatting changes, which you will receive in a follow-up email from a member of our team.

Please note that your manuscript will not be scheduled for publication until you have made the required changes, so a swift response is appreciated.

IMPORTANT: The editorial review process is now complete. PLOS will only permit corrections to spelling, formatting or significant scientific errors from this point onwards. Requests for major changes, or any which affect the scientific understanding of your work, will cause delays to the publication date of your manuscript.

If your institution or institutions have a press office, please notify them about your upcoming paper to help maximize its impact. If they'll be preparing press materials, please inform our press team as soon as possible -- no later than 48 hours after receiving the formal acceptance. Your manuscript will remain under strict press embargo until 2 pm Eastern Time on the date of publication. For more information, please contact digitalhealth@plos.org.

Thank you again for supporting Open Access publishing; we are looking forward to publishing your work in PLOS Digital Health.

Best regards,

Jing Tian, PhD

Academic Editor

PLOS Digital Health

***********************************************************

**Additional Editor Comments (if provided):**

Please check the entire manuscript for any inconsistencies as identified by the reviewers.

**Reviewer Comments (if any, and for reference):**

Reviewer's Responses to Questions

**Comments to the Author**

Reviewer #2: All comments have been addressed

**********

publication criteria?

Reviewer #2: Yes

**********

3. Has the statistical analysis been performed appropriately and rigorously?

Reviewer #2: No

**********

4. Have the authors made all data underlying the findings in their manuscript fully available (please refer to the Data Availability Statement at the start of the manuscript PDF file)?

The PLOS Data policy

Reviewer #2: Yes

**********

5. Is the manuscript presented in an intelligible fashion and written in standard English?

Reviewer #2: Yes

**********

Reviewer #2: The authors have responded carefully to the previous review. The paired bootstrap comparison with the pooled model is particularly helpful, as are the added SHAP results, q-values, feature-selection sensitivity analysis, and revised limitations. The discussion now makes a much clearer distinction between possible future screening applications and what can be concluded from the present cross-sectional analysis.

I have one remaining concern that should be corrected before publication.

1. Figure 7 does not appear consistent with the revised manuscript. Table 2 reports XGBoost AUCs of 0.794, 0.839, and 0.823 for Clusters 1–3, whereas the Figure 7 caption reports 0.785, 0.830, and 0.819. More importantly, the features shown in panels b–d do not match the cluster descriptions in the Results. For example, the text describes interpreter use, routine care location, sleep, and stress as important for Cluster 1, while the figure shows hepatitis B core antibody, occupation, survey-design variables, and blood pressure among the leading features. Similar discrepancies appear for Clusters 2 and 3. The authors should verify whether Figure 7 is from an earlier analysis and reconcile the figure, caption, and text.

The clinical wording could also be softened in two places. The Discussion still refers to “three clinically significant clusters,” and the Conclusion states that “latent depression-related phenotypes can be discerned.” Given the cross-sectional design and use of PHQ-9 screening data, “latent subgroups associated with differing depression prevalence” would be more accurate. This is consistent with the more cautious framing already added elsewhere.

Subject to these corrections, I am satisfied that the substantive concerns raised in the previous review have been addressed.

The earlier methodological and interpretive concerns have been addressed satisfactorily. I would support acceptance once the apparent inconsistencies involving Figure 7 are resolved and the remaining clinical-phenotype language is softened.

**********

what does this mean?). If published, this will include your full peer review and any attached files.

**Do you want your identity to be public for this peer review?**  If you choose “no”, your identity will remain anonymous but your review may still be made public.

For information about this choice, including consent withdrawal, please see our Privacy Policy

Reviewer #2: No

**********
